# Exploring Border Conditions for Spontaneous Emergence of Chirality in Allylboration of 1,2,3-Triazolic Aldehydes

**DOI:** 10.3390/ijms252011273

**Published:** 2024-10-20

**Authors:** Oleg A. Mikhailov, Mikhail E. Gurskii, Almira R. Kurbangalieva, Ilya D. Gridnev

**Affiliations:** 1 N. D. Zelinsky Institute of Organic Chemistry, Leninsky Prosp. 47, 119991 Moscow, Russia; mikhailov.o@ioc.ac.ru (O.A.M.); gurs@mail.ru (M.E.G.); 2 Biofunctional Chemistry Laboratory, A. Butlerov Institute of Chemistry, Kazan Federal University, 18 Kremlyovskaya Street, 420008 Kazan, Russia

**Keywords:** spontaneous emergence of chirality, stereoselective reactions, 1,2,3-triazole derivatives, allylboration, DFT (Density Functional Theory) computations, disperse interactions

## Abstract

A case of spontaneous chirality generation was observed during a synthetic project studying the allylboration of 1,2,3-triazolic aldehydes. Here, we present computational studies supported by experimental findings targeting the elucidation of border conditions required for the observation of spontaneous chirality generation in the reaction of 1-Ar-1*H*-1,2,3-triazole-4-carbaldehydes **1a,b** with triallylborane. Three possible sources of symmetry breaking were found computationally. Thus, dimerization of the initial reaction products, alcoholates **4a,b**, gives dimers **5a,b** (homochiral) and **6a,b** (heterochiral). The latter were computed to be more stable thermodynamically, which can lead to amplification of the initial stochastic imbalance of the enantiomers of **4a,b** via the reservoir mechanism. Furthermore, enantiomeric excess can be increased during the transfer of the second allylic group in the reaction of optically active boronates **4a,b** with **1a,b**, which was computed to be enantioselective due to the strong activating and stereoregulating properties of the 1,2,3-triazole group. In addition, reactions of borinic esters **8a,b**, products of the previous reaction with triallylborane, recovered in each case two molecules of **4a,b** of the same handedness, which can lead to additional chirality amplification. Experimentally, reactions of optically active alcohols (+)-*R*-**2a,b** with triallylborane provided chiral alcoholates **4a,b,** which were reacted with equivalent amounts of corresponding aldehydes **1a,b**. Unexpectedly, in two series of 10 experiments each, preferential formation of both enantiomers of the newly formed product was observed: seven times *S* and three times *R* in the case of **1a** and six times *S* and four times *R* in the case of **1b**.

## 1. Introduction

The discovery of the Soai reaction in 1995 [1] induced extensive studies in the fields of asymmetric autocatalysis and related areas. Together with mechanistic studies of asymmetric amplifying autocatalysis [2,3,4,5,6,7,8,9,10], other important features, e.g., the evident relevance of the Soai reaction to the emergency of chiral life on the Earth [11,12,13,14,15,16,17], are actively investigated and discussed.

The observation of the spontaneous emergency of chirality is based on two phenomena: stochastic mirror symmetry breaking leading to a scalemic product at the microscopic level, and a strong amplification mechanism bringing the ee of a macroscopic sample to detectable values. In addition, spontaneous mirror symmetry breaking in closed systems has significant kinetic restrictions [18,19,20] (vide infra in Section 3).

A microscopic imbalance inevitably emerges in any reaction leading to the creation of an asymmetric atom. In the vast majority of cases, it is lost in numerous simultaneously running processes of multi-stage reactions. Even if a microscopic imbalance remains untouched until the reaction is complete, its value is usually too small to be detected [13].

In view of these restrictions, it is no wonder that the Soai reaction so far remains unique in demonstrating actual experimental observations of the spontaneous emergence of chirality.

Here, we report a computational study seeking reaction pathways that could result in the realization of spontaneous chirality generation in the allylboration [21,22,23] of 1,2,3-triazolic aldehydes, as well as experimental observation of their enantioselective allylboration with chiral boronates ROB(Allyl)_2_ (R = *p-*ClC_6_H_4_, 2,4,6-Cl_3_C_6_H_2_).

## 2. Results and Discussion

### 2.1. Observation of Spontaneous Generation of Chirality in Allylboration

**Scheme 1:**
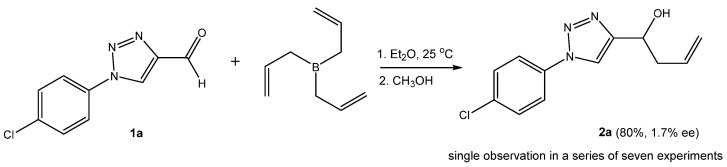
Spontaneous emergence of chirality during allylboration of aldehyde 1**a**.

**Scheme 2:**
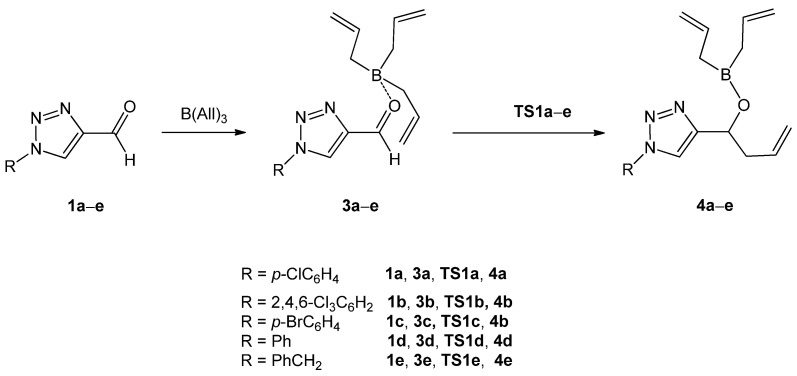
Allylboration of aldehydes **1a**–**e**.

In the course of our experiments studying non-catalyzed allylboration [24], we detected once the formation of a non-racemic product **2a(*S*)** (1.7% ee) from 1-(4-chlorophenyl)-1*H*-1,2,3-triazole-4-carboxaldehyde (**1a**) in a series of seven experiments; in another six cases racemic products were observed (Scheme 1). No optically active products were observed in the allylboration of a series of structurally similar aldehydes **1b**–**e** (Scheme 2).

Although we realized that the observed ee value was close to the detection level of HPLC analysis, being interested in the possibility of the spontaneous emergence of chirality in various chemical transformations, we undertook a computational study of the case, checking some conclusions experimentally.

### 2.2. DFT Study of the Mechanism of Allylboration

First, we computed the allylboration of aldehydes **1a**–**e** with triallylborane in order to check the dependence of reactivity on the nature of the substituent in a 1,2,3-triazole ring. Results are presented in Table 1.

**Figure 1:**
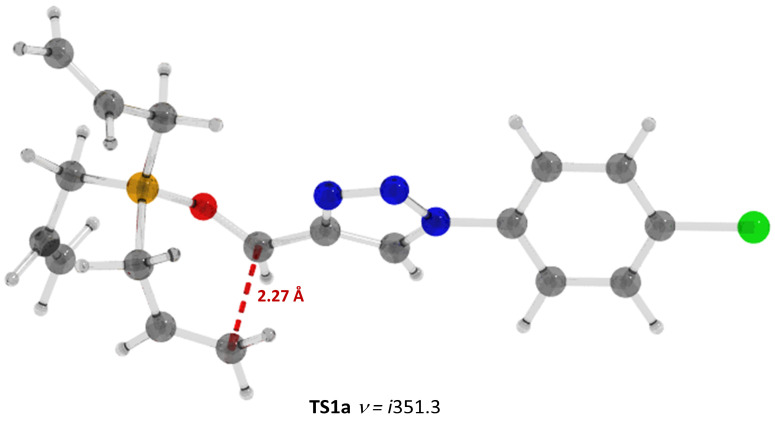
Computed structure of **TS1a**. Carbon: grey, hydrogen: white, oxygen: red, nitrogen: blue, chlorine: green, boron: yellow. The dashed red line represents the forming bond.

Initially, triallylborane reacted with aldehydes **1a**–**e** yielding adducts **3a**–**e**. They further underwent strongly exogonic allylation with a low activation barrier (Scheme 2). The reaction proceeded through a six-membered transition state (Figure 1) and yielded boronic esters **4a**–**e**.

When inspecting Table 1, one can notice significant differences in effective Gibbs free activation energies of allylboration for aldehydes **1a**–**e**. Apparently, electron withdrawing substituents in the 1,2,3-triazole ring increased the effective barrier of allylboration 2–3 times (compared to **1e**). As both reagents were non-chiral, this reaction was capable of producing only a microscopic difference in concentrations of *R* and *S* products. Accordingly, this reaction would inevitably compete with any further enantioselective stage. We decided to restrict our further analysis, with aldehydes **1a,b** exhibiting sufficiently high effective activation barriers in allylboration and having aryl substituents with different electronic properties. Importantly, the case of the spontaneous emergence of chirality was observed for **1a**.

### 2.3. DFT Study of the Dimerization of Boronates

**Scheme 3:**
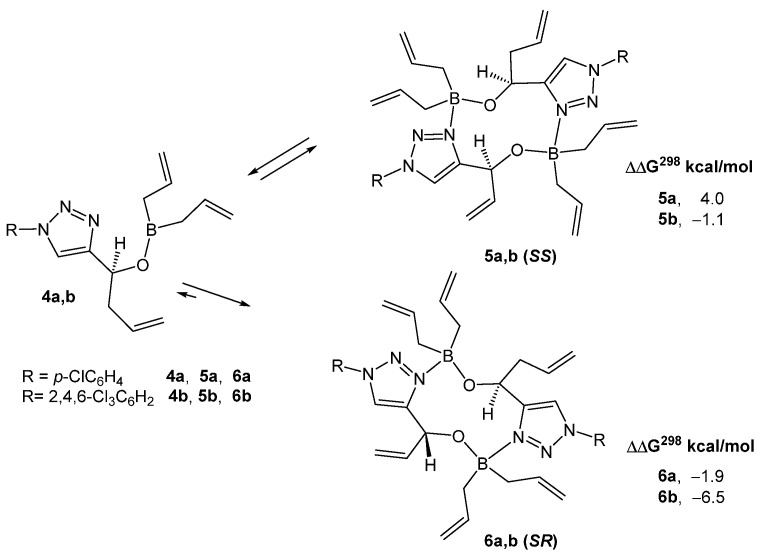
Dimerization of boronates **4a,b**.

Products of the amplifying Soai reaction dimerized, giving square Zn-O-Zn-O dimers, which were stable and embodied the resting state of the reaction [25]. In our case, attempts to compute similar homochiral and heterochiral B-O-B-O dimers resulted in their spontaneous isomerization to more stable macrocyclic dimers **5a,b** (homochiral) and **6a,b** (heterochiral) (Scheme 3).

**Figure 2:**
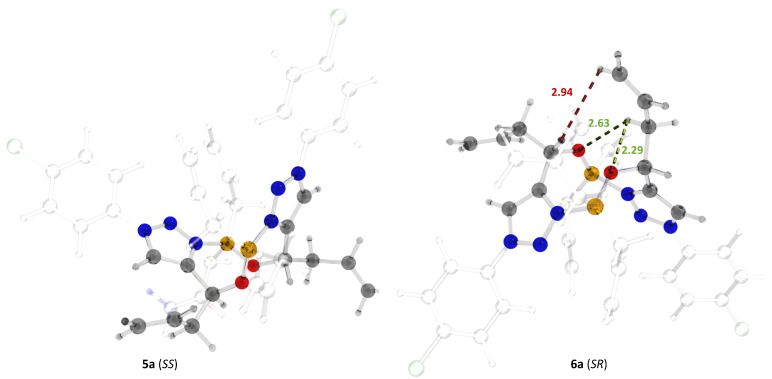
Optimized structures of homochiral and heterochiral dimers **5a** and **6a**. Carbon: grey, hydrogen: white, oxygen: red, nitrogen: blue, boron: yellow. Dashed lines and numbers (interatomic distances in Å) show weak intramolecular interactions responsible for the greater thermodynamic stability of **6a**: CH^…^HC: red, CH^…^O: green.

Each being bound to both oxygen and nitrogen atoms, boron atoms were significantly deactivated and could not bind triallylborane or additional molecules of **4**; i.e., they could not be catalysts for allylboration. On the other hand, both heterochiral dimers **6a**,**b** were notably more stable than the corresponding homochiral dimers **5a**,**b** (Scheme 3). The reason for this difference in stabilities was the dissimilar orientations of the allylic groups connected to the chiral centers; e.g., in **5a** both were equatorial and avoided any stabilizing contacts with the macrocycle, whereas in **6a** one of the allylic groups was axial and enjoyed three stabilizing disperse interactions: two CH⋯O and one CH⋯H (Figure 2).

The greater stability of heterochiral dimers opened the door for the realization of the reservoir mechanism [26,27,28] for the amplification of an initial microscopic imbalance in the concentration of *S*- and *R*-enantiomers of **4**, which could be further amplified in following transformations. In this model, the more thermodynamically stable heterochiral dimers **6a**,**b** served as non-reactive aggregates, whereas the solution became enriched with **4**(*S*).

### 2.4. Enantioselective Allylboration via Boronate Esters ***4***

**Scheme 4:**
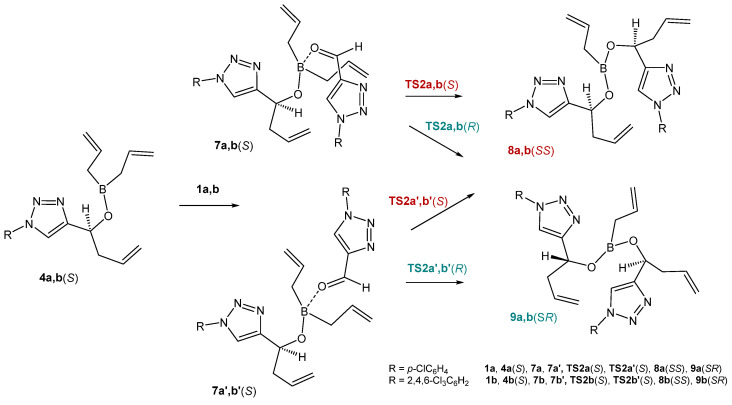
Computational results for enantioselective allylboration with boronic esters **4a**,**b**. Relative stabilities (free Gibbs energies) of transition states computed in diethyl ether, kcal/mol. Transition states and compound numbers for *S* and *R* pathways are shown in red and blue, respectively. Computations of these transition states in THF demonstrated essentially the same results.

**Figure 3:**
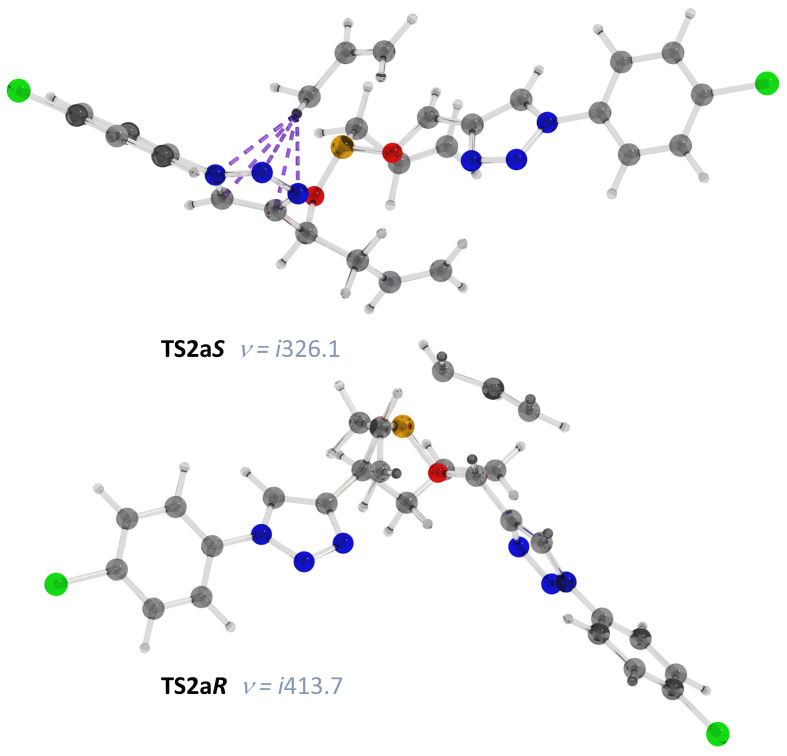
Optimized structures of transition states **TS2a**(*S*) and **TS2a**(*R*). Carbon: grey, hydrogen: white, oxygen: red, nitrogen: blue, chlorine: green, boron: yellow. Dashed violet lines illustrate strong CH^…^π interactions of the migrating allylic group with the triazolic ring in **TS2a**(*S*), which made it more stable than **TS2a**(*R*) for 5.4 kcal/mol.

Further, we analyzed the possibility of the involvement of the second allylic group in the boronate esters **4** in allylboration. Aldehyde **1a** coordinated to the *S*-enantiomer of boronate **4a**(*S*) yielding adduct **7a**(*S*) with two allylic groups symmetrically positioned at both sides of the coordinated carbonyl group. Nevertheless, the reactivity of these two groups can be significantly different. For example, **TS2a**(*S*) is 5.4 kcal/mol more stable than **TS2a**(*R*) (Scheme 4, Figure 3). Such a significant difference in the stabilities of transition states emerged due to the strong CH⋯π interactions between one of the protons of the allylic group involved in the migration process, which was possible only in **TS2a**(*S*) (Scheme 4). This finding demonstrates the effectiveness of the triazolic group as a stereoregulating group, even in relatively small molecules.

The numerous conformations possible in these molecules and transition states certainly affect their relative stability, which in turn affects the enantioselectivity of the reaction. Thus, the opposite orientation of the coordinated aldehyde in the **7a’**(*S*) difference in the stabilities of **TS2a’**(*S*) and **TS2a’**(*R*) was computed (5.4 and 3.0, respectively). A similar situation was observed with the difference in stabilities of **TS2b**(*S*) and **TS2b**(*R*) (5.0 kcal/mol) and **TS2b’**(*S*) and **TS2b’**(*R*) (3.7 kcal/mol) (Table 2).

The low activation barrier of this reaction is also of interest. Normally, boronates **4a**,**b** should be considered as less reactive allyboration reagents compared to triallylborane, as in **4a**,**b** the unoccupied 2*p* orbital of the boron atom is deactivated by the lone pair of the adjacent oxygen atom. Thus, our computations suggested that the strong CH⋯π interaction observed, e.g., in the **TS2**(*S*), could not only be a stereoregulating factor, but also facilitate the allylboration of the relatively unreactive boronate group.

### 2.5. Recovery of the Boronate Esters ***4***; Possibility of Autocatalytic Chirality Amplification

**Scheme 5:**
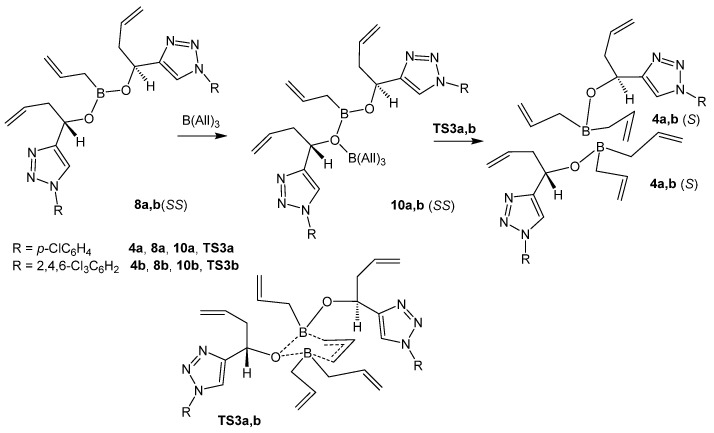
Recovery and amplification of boronate **4**(*S*) via reaction of **8**(*SS*) with triallylborane.

Compound **8**(*SS*) demonstrated no further allylboration potential in our computations. Evidently, the boron atom was sufficiently deactivated by two B-O bonds. On the other hand, we found that in reaction with triallylborane it can recover two molecules of **4**(*S*) (Scheme 5). Thus, the reaction sequence **4**(*S*) -> **8**(*SS*) -> 2 **4**(*S*) could lead to an amplification of chirality if any were accumulated in the earlier stages of the reactions sequence and the activation barriers were sufficiently low. In that case, **4** could be considered as an amplifying autocatalysis. Unfortunately, the effective activation barriers for this transformation (Table 3) were computed to be higher than corresponding effective activation barriers for initial allylboration (Table 1). Moreover, the reaction of **8a** was endogonic. Therefore, in these particular cases the amplification stage is unlikely to contribute to the overall outcome. Nevertheless, it was interesting to locate such a possibility computationally.

### 2.6. Experimental Simulation of Enantioselective Allylboration of ***1a***,***b*** with Optically Active Boronate Esters ***4a***,***b***

**Scheme 6:**
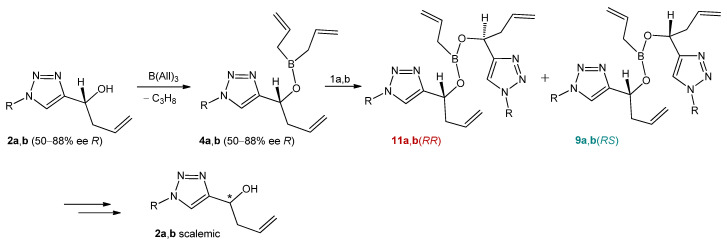
Reactions of optically active boronates **4a**,**b**(*R*) with one equivalent of corresponding aldehydes. Sign * indicates asymmetric carbon atom.

Recently, we prepared optically active homoallylic alcohols (*R*)-**4a** and (*R*)-**4b** by allylboration of **1a**,**b** with (*R*)-diisopinocamphenyl(allyl)borane [24]. This allowed us to design a series of experiments probing our conclusions derived from the results of the computational studies (Scheme 6).

Scalemic boronates **4a**,**b** were generated by reacting optically active homoallylic alcohols **2a**,**b** with triallylborane. Next, they were reacted with corresponding aldehyde, and the reaction mixture was worked up using the usual method. The ee’s of the resulting homoallylic alcohols were determined by HPLC analysis. Complete results are shown in Table S1 (see Supplementary Materials). HPLC traces and NMR spectra of the products are shown in Figures S1–S72.

**Figure 4:**
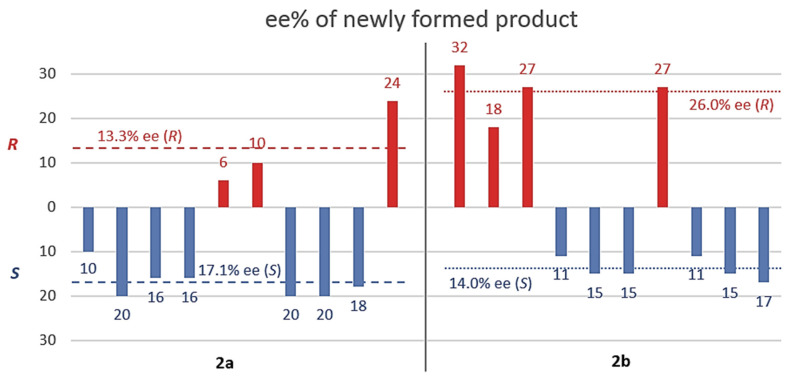
Results of two series of experiments, 10 experiments each for allylboration of **1a** and **1b** with optically active boronates **4a** and **4b**, respectively. Medium values are shown in dashed lines.

After exploring various reaction conditions (see Table S1) we chose the reaction in THF with generating boronates **4a**,**b** at 25 °C and their reactions with **1a**,**b** at 50 °C for more detailed investigation (Figure 4).

The most expected result would be the observation of halved ee values (non-selective reactions) or the approximate conservation of the optical yield of the starting boronate (stereoselective reactions: *R*-boronate gives *R*-product, and *S*-boronate gives *S*-product).

Actually, the formation of **2a** and **2b** with notable ee’s of both handedness was observed for the newly formed products. Although this result cannot be rationalized explicitly now, we considered the possibility of the conformational equilibria in **4a**,**b** being involved. The computed differences in free energies of the transition states leading to *R* and *S* enantiomers are dependent on the particular conformation acquired by the molecule during reaction (and there are numerous possibilities). Activation barriers can be low even for the reaction leading to the formation of the opposite enantiomer of the newly formed product (for example TS2**a**’(*R*) in Table 2). Additionally, one cannot exclude the involvement of dynamic thermodynamic resolution in these reactions [29].

Nevertheless, some conclusions can be drawn even now:

(a) The reaction of an optically active **4a**,**b** with **1a**,**b** can be an enantioselective stage of the allylboration of **1a**,**b** with triallylborane.
(b) In these two cases, it is unlikely to result in macroscopic ee values due to low optical yields.
(c) It might be possible to regulate the selectivity of this reaction by changing its conditions.

## 3. Discussion

Although we cannot at the moment give a clear mechanistic explanation of the experimental results, especially the formation of mainly *S*-enantiomer of the newly formed product in the reaction of **4a** and **4b** enriched with *R*-enantiomer, we discuss the computational results from the viewpoint of the border conditions that might theoretically result in the possibility of amplifying autocatalytic reactions.

**Figure 5:**
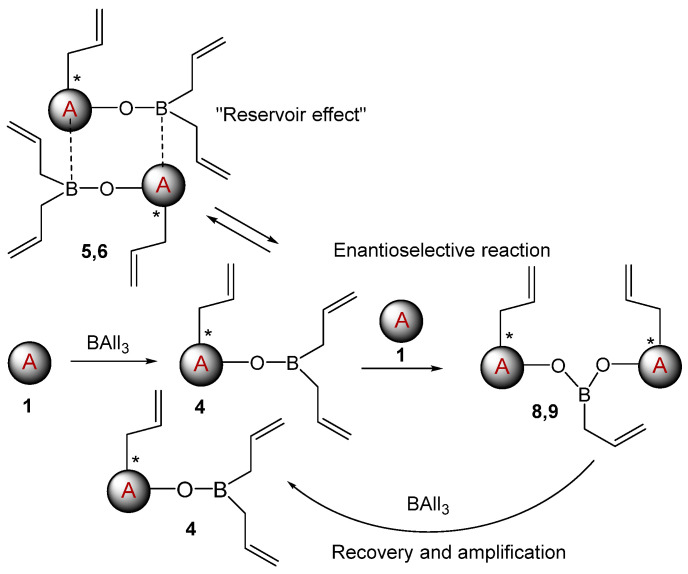
Computed reactions sequence that may result in asymmetric amplification in the allylboration of, e.g., triazolic aldehyde **1a**. Sign * indicates the presence of asymmetric carbon atom in the structure.

The outline of the computed reactions sequence that might result in asymmetric amplification is shown in Figure 5.

The initial allylboration of **1** creates an asymmetric center, yielding racemic boronate **4**. As any other racemate it inevitably contains a microscopic excess of one of the enantiomers, the handedness of which is determined stochastically [13].

The dimerization of **4** can result in the reservoir effect, as heterochiral dimers **6** are more stable than homochiral dimers **5** (Scheme 3). This means that the microscopic excess of one of the enantiomers of **4** present in solution can be increased at this stage due to the binding of the opposite enantiomer of **4** in a more stable heterochiral dimer **6**.

The second molecule of **1** can be enantioselectively allylborated with **4** (Scheme 4); this reaction was computed to be faster than the initial allylboration with triallylborane due to the activation effect of the 1,2,3-triazole ring. If the level of enantioselection is high enough, this could lead to a linear increase in the enantiomeric excess acquired during the previous stage.

The chirality could be further amplified in the reaction of **8** with triallylborane, which has the capability of recovering two molecules of **4**. Interestingly, this gives reasons to consider the whole process as autocatalytic, with **4** working as an autocatalyst.

However, reality is often quite far from idealized speculations. To begin with, the formation of dimers **5a**(*SS*) and **6a**(S*R*) was computed to be endogonic for 4.0 and 1.1 kcal/mol, respectively, meaning that only about 16% **6a**(S*R*) is present in the reaction mixture. These numbers suggest that the reservoir effect (if any) would be weak in this case.

On the other hand, the dimerizations of **4b** yielding both the homochiral dimer **5b**(*SS*) and the heterochiral dimer **6b**(*SR*) were computed to be exogonic for 6.0 and 1.9 kcal/mol, respectively (Scheme 3). These values seem to be much more favorable for the realization of the reservoir mechanism, implying strong binding in the heterochiral dimer and a reasonably accessible monomer **4b** via the dissociation of **5b**(*SS*).

The computational results for the dimerization of **4a**,**b** also show that, depending on the substituent in the 1,2,3-triazole ring, the values of thermodynamic and kinetic parameters can vary significantly. Apparently, the same is true for the kinetic parameters of the next two stages (e.g., see Table 2), so a clever and diligent variation of the structures of the substrates might ultimately result in a spontaneous emergence of chirality in a formally non-catalytic reaction, according to the Frank’s scheme [30].

The main obstacle for realization of such a scenario is the rapid reaction of triallylborane with triazolic aldehydes (even at −100 °C). On the other hand, it is known that the observation of the spontaneous generation of macroscopic chirality can only be expected in relatively slow reactions, allowing the initially formed racemic product to accumulate in the reaction vessel for a certain characteristic time before the racemic state becomes unstable and comes to a bifurcation point [18], leading to the appearance of significant disbalance in the concentrations of enantiomers in the resulting product [19,20].

Fortunately, the reactivity of allylboration reagents can be significantly varied in a broad range via introducing electron-donating substituents (RO or RN) instead of one or two allylic groups. We plan to use the results of this work in further studies with less reactive allylboration reagents.

In addition, computational studies demonstrated a feature of the 1,2,3-triazolic group that was probably previously unnoticed among its numerous specific properties [31,32,33], i.e., its remarkable ability to participate in intramolecular disperse interactions that result in, e.g., the stereoselective transformation of **4a**(*S*) to **8a**(*SS*).

We plan to develop further this important property of the triazolic ring for the design and synthesis of new chiral ligands and catalysts. For example, it may serve as a still more potent stereoregulating group than *t*-Bu [34].

## 4. Materials and Methods

### 4.1. Experimental Details

All reactions were carried out using standard Schlenk techniques under an argon atmosphere in oven-dried glassware with magnetic stirring. All solvents were purified and distilled using standard procedures. Triallylborane was prepared by a known procedure [35]. Solvents were additionally degassed by three pump–freeze–thaw cycles. Analytical thin layer chromatography (TLC) was carried out on Merck TLC plates (silica gel 60 F_254_, 0.25 mm) using UV light (254 nm) as the visualizing agent. Silica gel 60A (Acros Organics, Geel, Belgium, 400–230 mesh, 0.040–0.063 mm) was used for open-column chromatography. The ee measurements were performed via HPLC analysis on an HPLC system equipped with chiral stationary phase columns (AD-H, AS-H, OD-H, and OJ-H), with detection at 220 or 254 nm.

### 4.2. Computational Details

Geometry optimizations were performed without any symmetry constraints (C1 symmetry) using the ωB97XD functional [36] as implemented in the Gaussian09 software package [37]. Frequency calculations were undertaken to confirm the nature of the stationary points, yielding one imaginary frequency for all transition states (TSs) and zero for all minima. Constrained energy hypersurface scans were conducted to investigate molecular reactivity and to locate viable reaction channels. Where low-lying barriers were estimated, frequency calculations were performed at the crude saddle points, and the obtained force constants were used to optimize the transition state structures employing the Berny algorithm [38]. All atoms were described with the 6–31G(d,p) basis set in the geometry optimization and frequency calculation [39,40,41,42,43,44]. Non-specific solvation was introduced using the SMD continuum model (diethyl ether) [45].

## 5. Conclusions

Strictly speaking, we were unable to confirm reliably the authenticity of our initial observation or to exclude entirely the possibility of its legitimacy. Nevertheless, the combined results of experimental and computational studies suggest that it was not entirely impossible. These results imply that with the lucky combination of kinetic and thermodynamic parameters of a multi-stage reaction, spontaneous emergence of chirality may become a reality in, e.g., the allylboration of 1,2,3-triazolic aldehydes in which two reactive groups remain untouched in the primary product of the reaction with an asymmetric carbon atom emerged after initial allylboration.

## Figures and Tables

**Table 1:** Computed parameters of allylboration (Scheme 2).

Starting Compound	Gibbs Free Energy for Formation of 3, kcal/mol	Gibbs Free Activation Energy, kcal/mol	Effective Gibbs Free Activation Energy, kcal/mol	Imaginary Frequency *ν*, cm^–1^	Gibbs Free Energy of the Reaction, kcal/mol
**1a**	10.6	7.4	18.0	*i*351.3	–26.3
**1b**	8.8	11.2	19.9	*i*334.6	–28.4
**1c**	5.1	16.7	21.8	*i*381.1	–28.0
**1d**	3.8	15.8	19.6	*i*356.7	–26.5
**1e**	6.6	7.4	14.1	*i*353.1	–27.1

**Table 2:** Computed parameters of stereoselective allylboration in diethyl ether ^a,b^ (Scheme 4).

Starting Compound	Gibbs Free Energy for Formation of 7a,b	Gibbs Free Activation Energy, kcal/mol		Effective Free Activation Energy, kcal/mol	Imaginary Frequency *ν*, cm^–1^	Gibbs Free Energy of the Reaction, kcal/mol
4a	9.9	TS2a(*S*)	4.3	14.2	*i*326.1	–30.4
		TS2a’(*S*)	–1.5	8.4	*i*346.9	
		TS2a(*R*)	9.7	19.6	*i*413.7	–40.6
		TS2a’(*R*)	1.5	11.5	*i*233.3	
4b	14.1	TS2b(*S*)	8.1	22.2	*i*328.5	–21.5
		TS2b’(*S*)	4.7	18.7	*i*352.9	
		TS2b(*R*)	13.1	27.1	*i*370.1	–20.6
		TS2b’(*R*)	8.4	22.5	*i*398.7	

^a^ Computations in THF demonstrated essentially the same results. ^b^ Transition states for *S* and *R* pathways are shown in red and blue, respectively.

**Table 3:** Computed parameters of the reaction of **8**(*SS*) with triallylborane (Scheme 5).

Starting Compound	Gibbs Free Energy for Formation of 10	Gibbs Free Activation Energy, kcal/mol	Effective Gibbs Free Activation Energy, kcal/mol	Imaginary Frequency *ν*, cm^–1^	Gibbs Free Energy of the Reaction, kcal/mol
**8a**	4.7	18.9	23.5	*i*78.20	4.1
**8b**	11.3	20.2	31.5	*i*116.05	–6.8

## Data Availability

All data are included in the manuscript and the Supplementary Materials.

## Supplementary Materials

The following supporting information can be downloaded at: https://www.mdpi.com/article/10.3390/ijms252011273/s1.
