# Supplementary material for: Exploring Border Conditions for Spontaneous Emergence of Chirality in Allylboration of 1,2,3-Triazolic Aldehydes
Source: Int J Mol Sci. 2024 Oct 20;25(20):11273. doi: 10.3390/ijms252011273 (PMC11509031; doi:10.3390/ijms252011273)
Supplement: Supplementary file 1 [file ijms-25-11273-s001.zip › ijms-3213963-supplementary.pdf]

Supplementary Materials

Exploring Border Conditions for Spontaneous Emergence of Chirality in Allylboration of 1,2,3-Triazolic Aldehydes

Oleg A. Mikhailov,[1] Mikhail E. Gurskii,[1] Almira R. Kurbangalieva,[2]* and Ilya D. Gridnev[1]*

[1] N. D. Zelinsky Institute of Organic Chemistry, Leninsky prosp. 47, 119991 Moscow, Russia

[2] Biofunctional Chemistry Laboratory, A. Butlerov Institute of Chemistry, Kazan Federal University, 18 Kremlyovskaya Street, 420008 Kazan, Russia

* Correspondence: IDG ilyaiochem@gmail.com; ARK akurbang@kpfu.ru

Content

1. Table S1. Reactions of optically active boronates (*R*)-4a,b with one equivalent of 1a,b (Scheme 6)* 3

2. General procedure for conducting reactions with optically active alcohols (Table S1) 4

3. HPLS data for alcohols 2a–2e 5

4. NMR spectra of newly formed alcohols 2a, 2b (Table 4) 21

5. Cartesian coordinates of optimized structures 36

1. Table S1. Reactions of optically active boronates (*R*)-4a,b with one equivalent of 1a,b (Scheme 6)*

<table>
<tr><th>Entry</th><th>Starting Compounds</th><th>ee(%) of starting 2</th><th>Solvent</th><th>Temperature for generating 4, °C</th><th>Temperature for addition of 1, °C</th><th>ee(%)</th><th>ee(%) of newly formed 2</th></tr>
<tr><td>1</td><td rowspan="13">1a, (R)-4a</td><td rowspan="4">50</td><td rowspan="10">THF</td><td rowspan="10">25</td><td rowspan="10">50</td><td>20</td><td>10(S)</td></tr>
<tr><td>2</td><td>15</td><td>20(S)</td></tr>
<tr><td>3</td><td>17</td><td>16(S)</td></tr>
<tr><td>4</td><td>17</td><td>16(S)</td></tr>
<tr><td>5</td><td rowspan="6">70</td><td>38</td><td>6(R)</td></tr>
<tr><td>6</td><td>40</td><td>10(R)</td></tr>
<tr><td>7</td><td>25</td><td>20(S)</td></tr>
<tr><td>8</td><td>25</td><td>20(S)</td></tr>
<tr><td>9</td><td>26</td><td>18(S)</td></tr>
<tr><td>10</td><td>47</td><td>24(R)</td></tr>
<tr><td>11</td><td rowspan="2">81</td><td rowspan="3">Ether</td><td>25</td><td>25</td><td>31</td><td>19(S)</td></tr>
<tr><td>12</td><td>–100</td><td>–100</td><td>16</td><td>49(S)</td></tr>
<tr><td>13</td><td>73</td><td>25</td><td>–100</td><td>37</td><td>0</td></tr>
<tr><td>14</td><td rowspan="16">1b, (R)-4b</td><td>88</td><td rowspan="2">THF</td><td rowspan="2">25</td><td rowspan="2">25</td><td>45</td><td>2(R)</td></tr>
<tr><td>15</td><td>78</td><td>42</td><td>6(R)</td></tr>
<tr><td>16</td><td>78</td><td rowspan="10">THF</td><td rowspan="10">25</td><td rowspan="10">50</td><td>55</td><td>32(R)</td></tr>
<tr><td>17</td><td>88</td><td>53</td><td>18(R)</td></tr>
<tr><td>18</td><td rowspan="8">67</td><td>47</td><td>27(R)</td></tr>
<tr><td>19</td><td>28</td><td>11(S)</td></tr>
<tr><td>20</td><td>26</td><td>15(S)</td></tr>
<tr><td>21</td><td>26</td><td>15(S)</td></tr>
<tr><td>22</td><td>47</td><td>27(R)</td></tr>
<tr><td>23</td><td>28</td><td>11(S)</td></tr>
<tr><td>24</td><td>26</td><td>15(S)</td></tr>
<tr><td>25</td><td>25</td><td>17(S)</td></tr>
<tr><td>26</td><td>88</td><td rowspan="3">THF</td><td rowspan="3">25</td><td>65</td><td>49</td><td>10(R)</td></tr>
<tr><td>27</td><td>78</td><td>0</td><td>42</td><td>6(R)</td></tr>
<tr><td>28</td><td>78</td><td>–50</td><td>39</td><td>0</td></tr>
<tr><td>29</td><td>39</td><td>Ether</td><td>–100</td><td>–100</td><td>25</td><td>11(R)</td></tr>
</table>

[a]Please note that in the experiments **4a**(*R*) and **4b**(*R*) were used, whereas the computations were done for **4a**(*S*) and **4b**(*S*)

2. General procedure for conducting reactions with optically active alcohols (Table S1)

*for **2a***

Chiral alcohol **2a** (27 mg, 0.11 mmol, 1 eq.) was placed in a 25 mL Schlenk flask equipped with a magnetic stirrer and a septum and filled with argon, repeating the argon-vacuum cycle 3 times. Then dry diethyl ether (10 mL) or THF (5 mL) was added, stirred until the substance was completely dissolved and triallylborane (0.015 mL, 0.11 mmol, 1 eq.) was added. Aldehyde **1a** (23 mg, 0.11 mmol, 1 eq.) was dissolved in ether (10 mL) or THF (5 mL) and, after 5 minutes of stirring, was dipped into the reaction mixture. The reaction was monitored using TLC. In each case, the aldehyde conversion was complete. After 5 minutes, 5 mL of methanol was added. After stirring for 1 minute, all volatile components were removed in vacuum, the resulting residue was subjected to additional purification by column chromatography, eluting with a mixture of ethyl acetate with petroleum ether (1 : 1).

*for **2b***

Chiral alcohol **2b** (24 mg, 0.08 mmol, 1 eq.) was placed in a 25 mL Schlenk flask equipped with a magnetic stirrer and a septum and filled with argon, repeating the argon-vacuum cycle 3 times. Then dry diethyl ether (10 mL) or THF (5 mL) was added, stirred until the substance was completely dissolved and triallylborane (0.013 mL, 0.08 mmol, 1 eq.) was added. Aldehyde **1b** (21 mg, 0.08 mmol, 1 eq.) was dissolved in ether (10 mL) or THF (5 mL) and, after 5 minutes of stirring, was dipped into the reaction mixture. The reaction was monitored using TLC. In each case, the aldehyde conversion was complete. After 5 minutes, 5 mL of methanol was added. After stirring for 1 minute, all volatile components were removed in vacuum, the resulting residue was subjected to additional purification by column chromatography, eluting with a mixture of ethyl acetate with petroleum ether (1 : 1).

In entries 12, 13, 27– 29 – The corresponding stages were carried out with a cooling bath made of a mixture of methanol and liquid nitrogen (–100°C), a mixture of *m*-xylene and liquid nitrogen (–50°C) or ice (0°C). It was then warmed up to room temperature before adding methanol.

In entries 1–10, 16–26 – The reaction mixture was warmed up to the predetermined temperature before adding the aldehyde.

3. HPLS data for alcohols 2a–2e

Previously, we synthesized optically active alcohols **2a**–**2e** by reacting the corresponding aldehydes with (*R*)-diisopinocamphenyl(allyl)borane [24]. The rotation angles [α] of the products were measured and all of them showed a positive value, ranging from +8.7 to +20.8 (c 1.00 $CHCl_3$), indicating the same handedness for **2a**–**2e**. Additionally, crystals were obtained for two of the compounds (**4c** and **4d**), which were analyzed by X-ray diffraction. The results showed that the predominant enantiomer was *R*, consistent with computational studies. Based on these findings, the peaks in HPLC were assigned: the first peak corresponds to the *R* isomer and the second peak corresponds to *S*.

Conditions: hexane : 2-propanol = 90:10,
Flow rate = 1.0 mL/min, λ = 220 nm, Chiralpak AD.

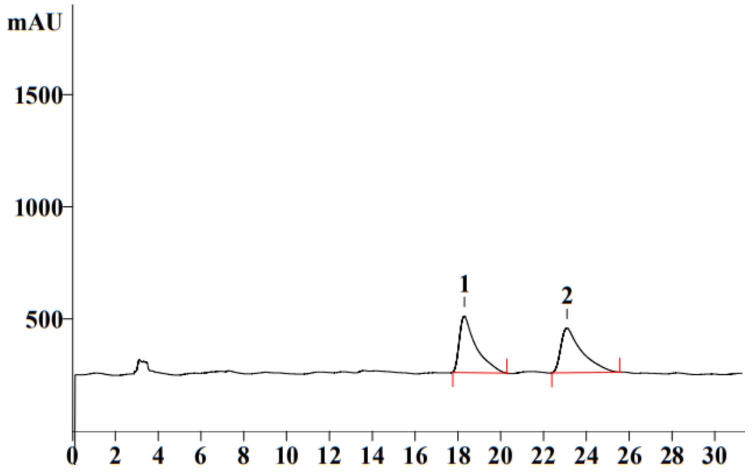

	Peak 1	Peak 2
Retention Time (min)	18.30	23.09
Relative Area (%)	50.17	49.83

Figure S1. HPLC chromatogram for compound **2a** (racemic).

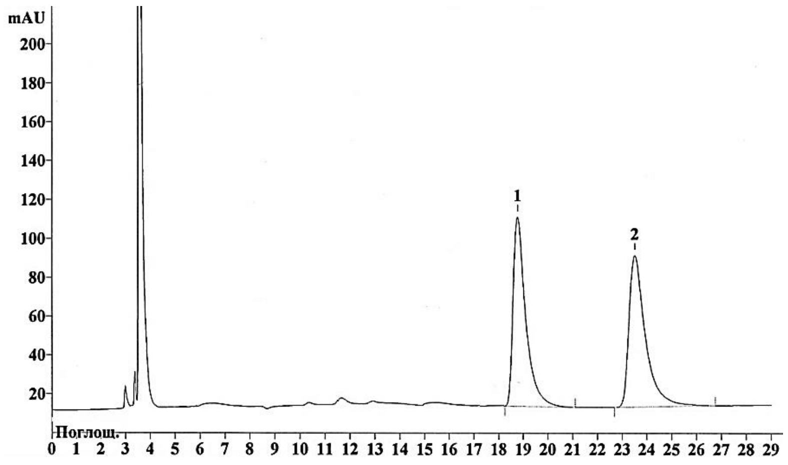

	Peak 1	Peak 2
Retention Time (min)	18.78	23.52
Relative Area (%)	49.14	50.86

Figure S2. HPLC chromatogram for compound **2a** (1.7% ee).

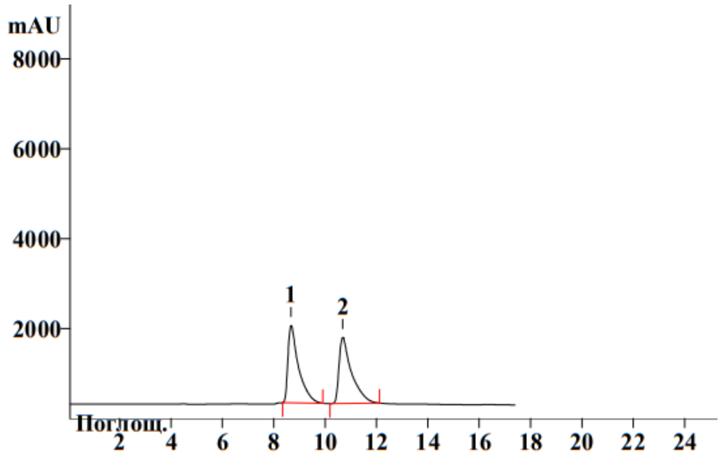

	Peak 1	Peak 2
Retention Time (min)	8.67	10.68
Relative Area (%)	49.74	50.26

Figure S3. HPLC chromatogram for compound **2b** (racemic).

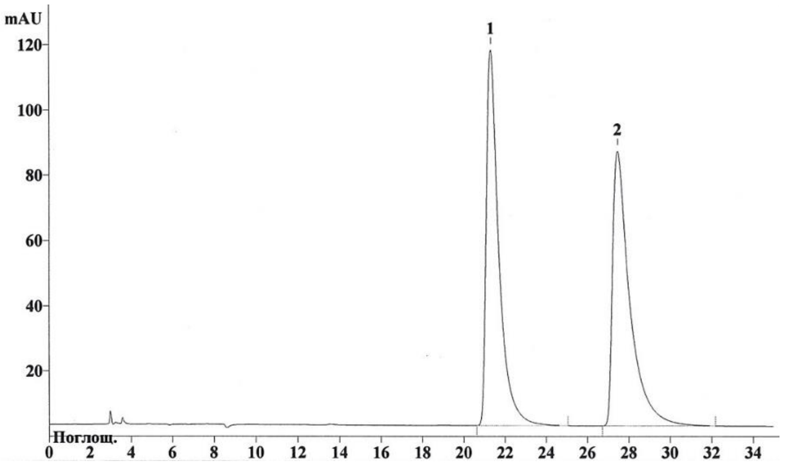

	Peak 1	Peak 2
Retention Time (min)	21.31	27.47
Relative Area (%)	50.03	49.97

Figure S4. HPLC chromatogram for compound **2c** (racemic).

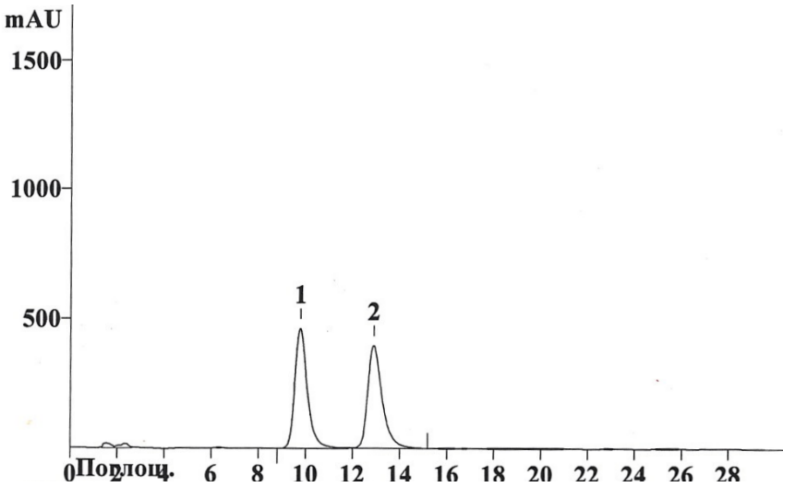

	Peak 1	Peak 2
Retention Time (min)	9.77	12.89
Relative Area (%)	50.22	49.78

Figure S5. HPLC chromatogram for compound **2d** (racemic).

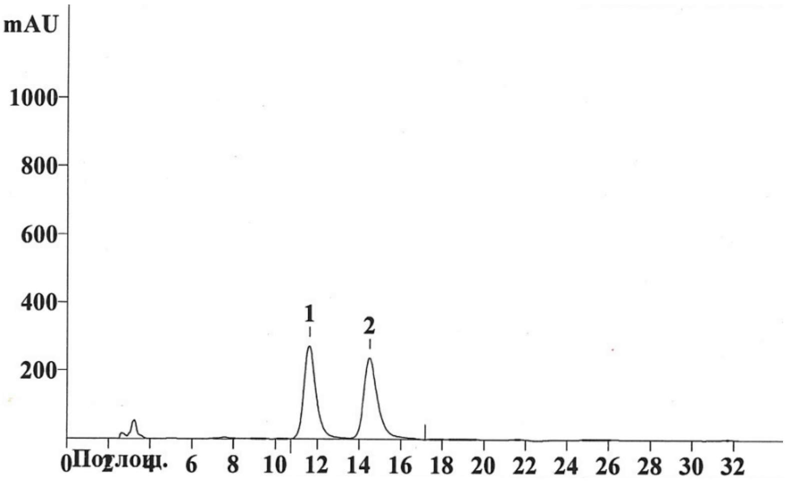

	Peak 1	Peak 2
Retention Time (min)	11.61	14.50
Relative Area (%)	49.84	50.16

Figure S6. HPLC chromatogram for compound **2e** (racemic).

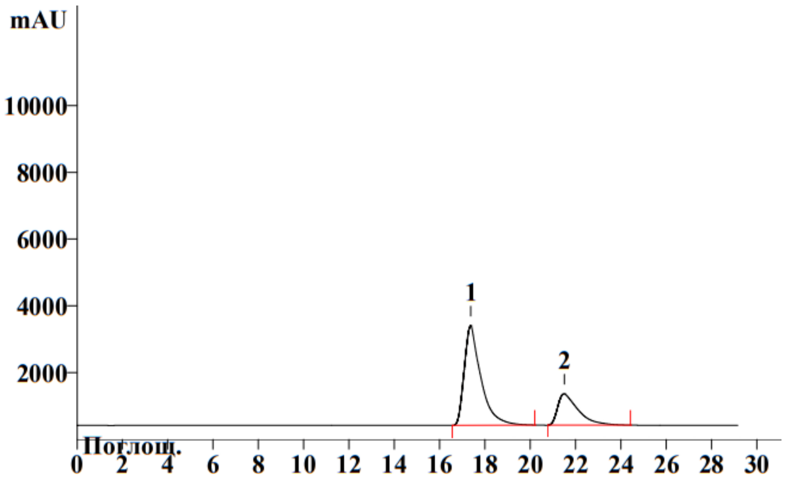

	Peak 1	Peak 2
Retention Time (min)	17.56	21.73
Relative Area (%)	74.93	25.07

Figure S7. HPLC chromatogram of starting **2a** (Entries 1–4).

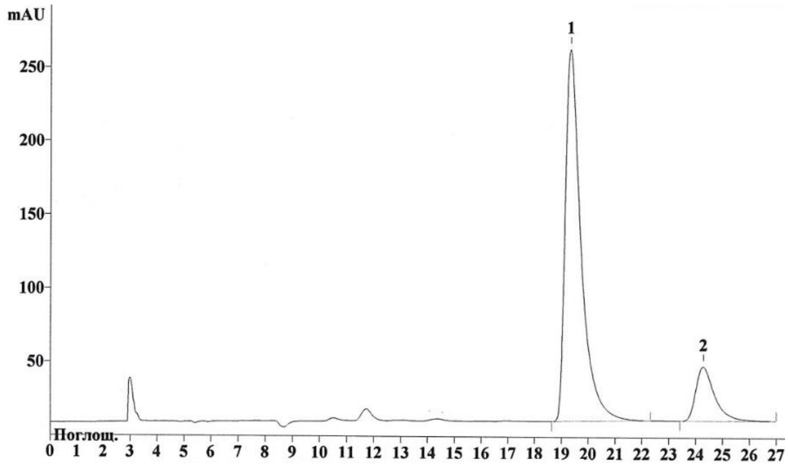

	Peak 1	Peak 2
Retention Time (min)	19.33	24.28
Relative Area (%)	85.17	14.83

Figure S8. HPLC chromatogram of starting **2a** (Entries 6–10).

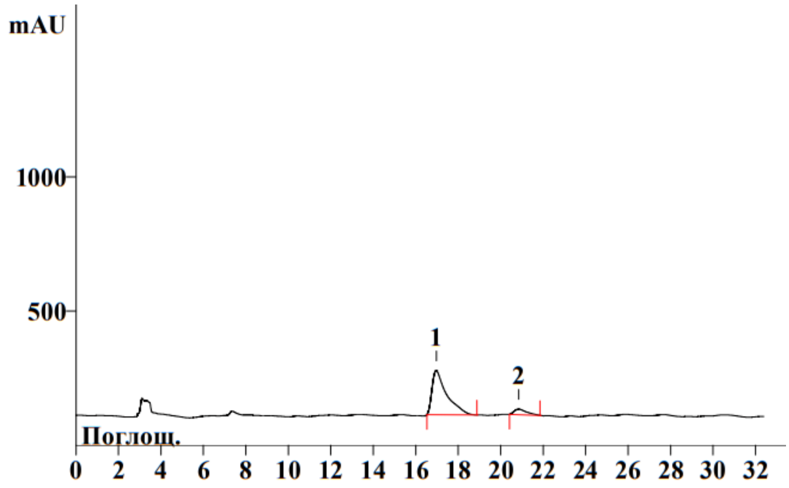

	Peak 1	Peak 2
Retention Time (min)	17.32	21.26
Relative Area (%)	90.39	9.61

Figure S9. HPLC chromatogram of starting **2a** (Entries 11, 12).

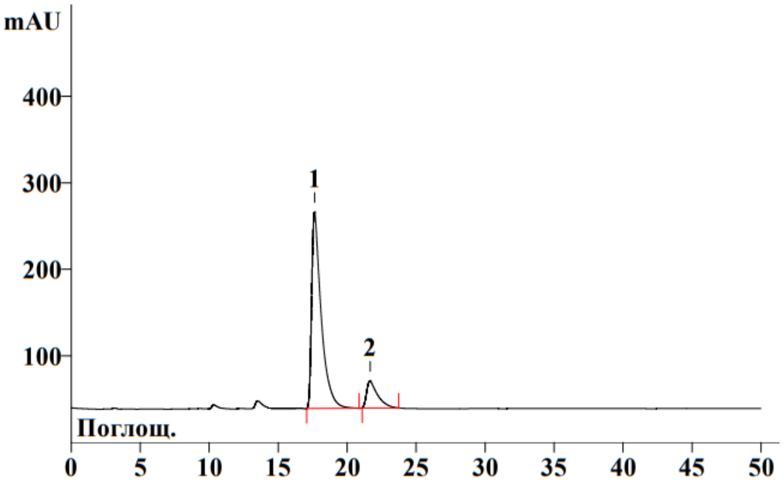

	Peak 1	Peak 2
Retention Time (min)	18.15	21.97
Relative Area (%)	86.41	13.59

Figure S10. HPLC chromatogram of starting **2a** (Entry 13).

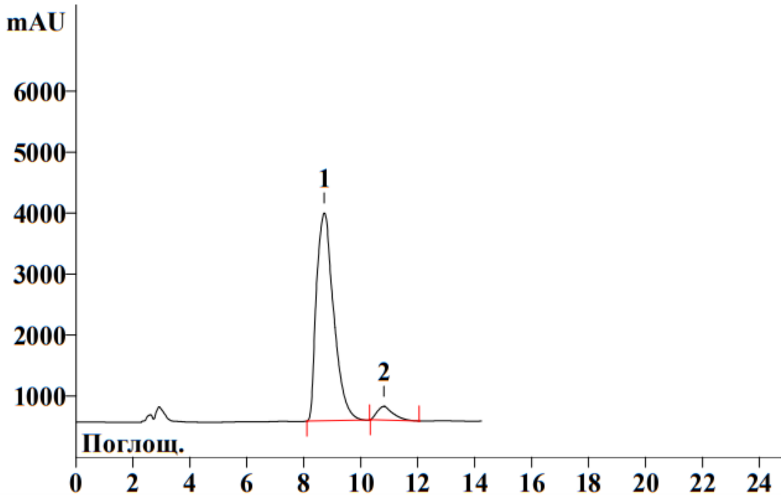

	Peak 1	Peak 2
Retention Time (min)	8.72	10.81
Relative Area (%)	94.30	5.70

Figure S11. HPLC chromatogram of starting **2b** (Entries 14, 17, 26).

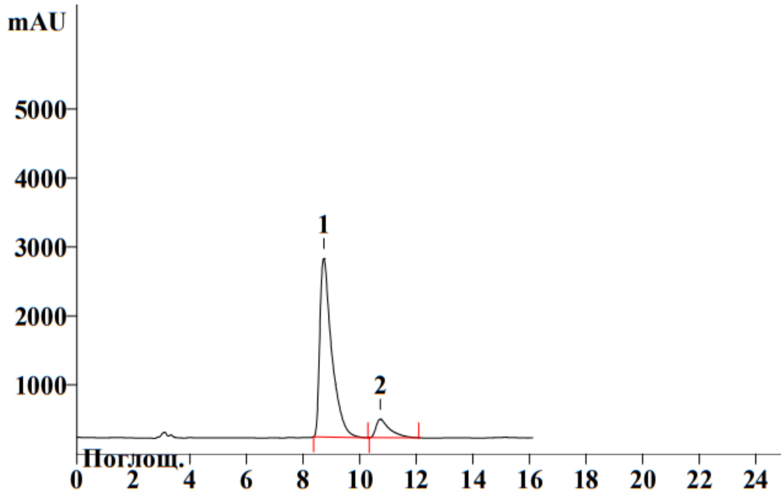

	Peak 1	Peak 2
Retention Time (min)	8.73	10.72
Relative Area (%)	89.28	10.72

Figure S12. HPLC chromatogram of starting **2b** (Entries 15, 16, 27, 28).

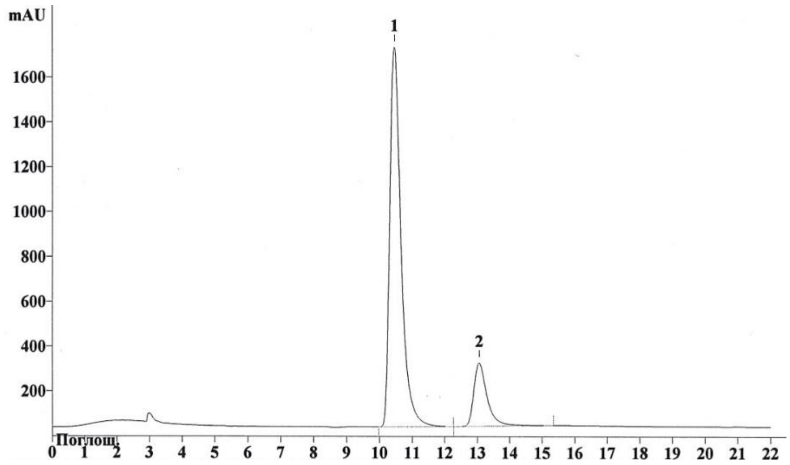

	Peak 1	Peak 2
Retention Time (min)	10.47	13.06
Relative Area (%)	83.42	16.58

Figure S13. HPLC chromatogram of starting **2b** (Entries 18–25).

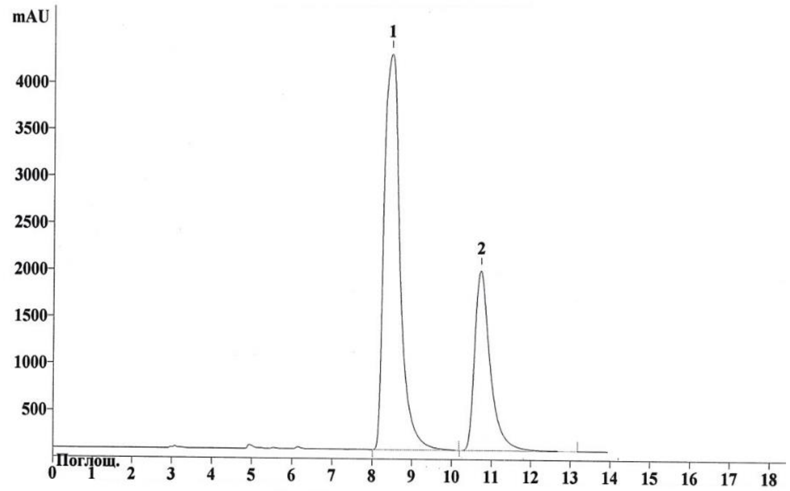

	Peak 1	Peak 2
Retention Time (min)	8.65	10.88
Relative Area (%)	69.45	30.55

Figure S14. HPLC chromatogram of starting **2b** (Entry 29).

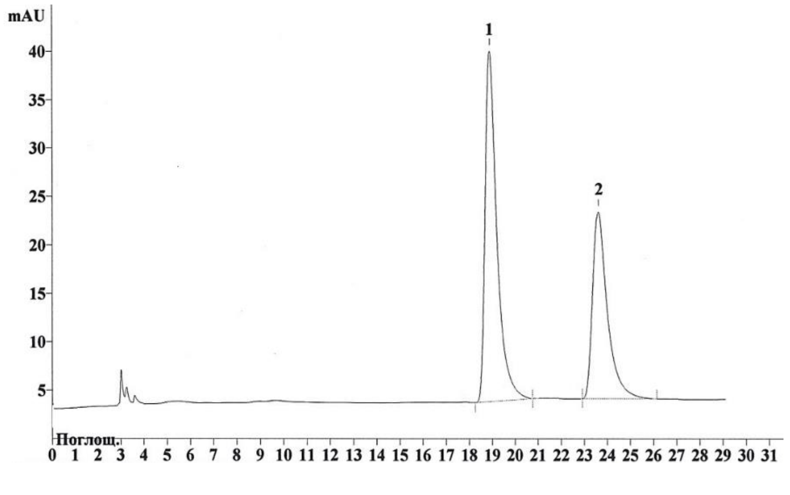

	Peak 1	Peak 2
Retention Time (min)	18.89	23.62
Relative Area (%)	60.03	39.97

Figure S15. HPLC chromatogram of **2a** after isolation (Entry 1).

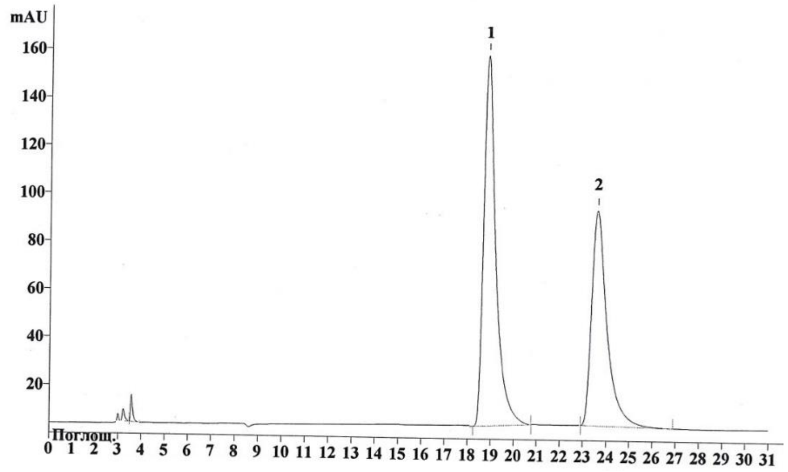

	Peak 1	Peak 2
Retention Time (min)	18.81	23.59
Relative Area (%)	57.78	42.22

Figure S16. HPLC chromatogram of **2a** after isolation (Entry 2).

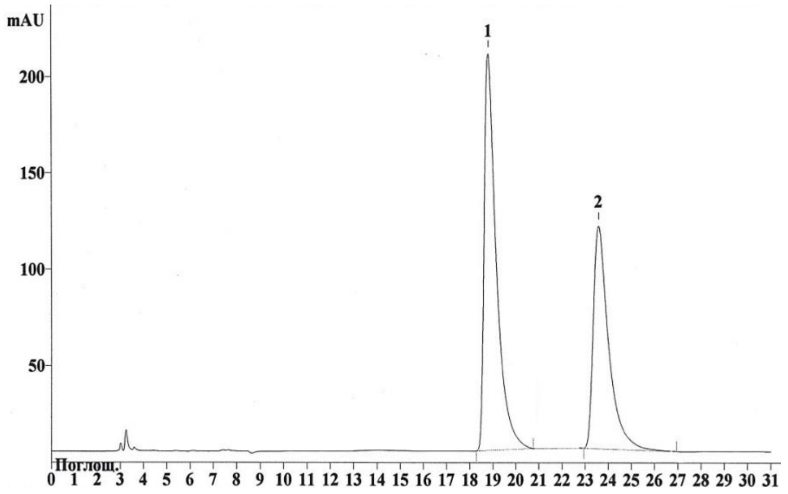

	Peak 1	Peak 2
Retention Time (min)	18.81	23.59
Relative Area (%)	58.53	41.47

Figure S17. HPLC chromatogram of **2a** after isolation (Entry 3).

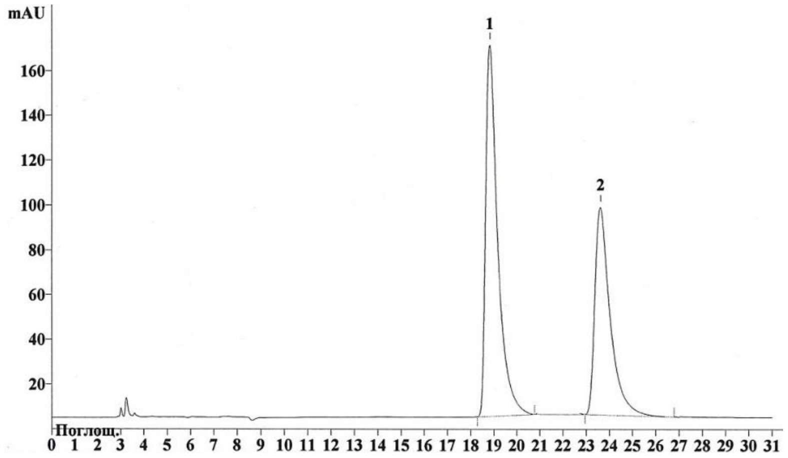

	Peak 1	Peak 2
Retention Time (min)	18.83	23.62
Relative Area (%)	58.72	41.28

Figure S18. HPLC chromatogram of **2a** after isolation (Entry 4).

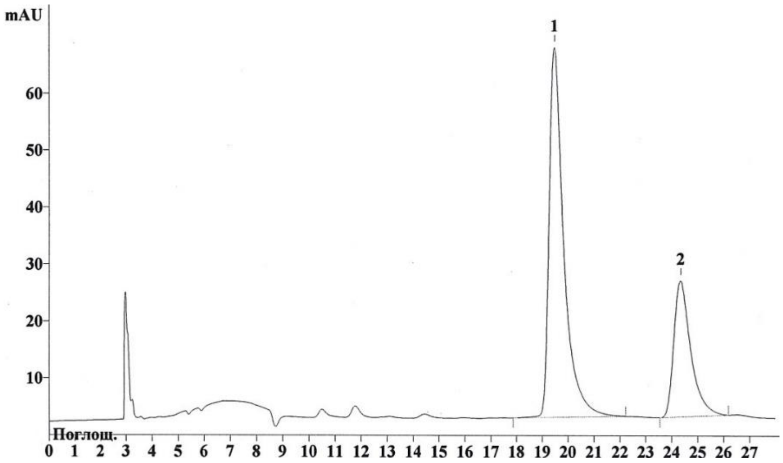

	Peak 1	Peak 2
Retention Time (min)	19.48	24.35
Relative Area (%)	70.24	29.76

Figure S19. HPLC chromatogram of **2a** after isolation (Entry 5).

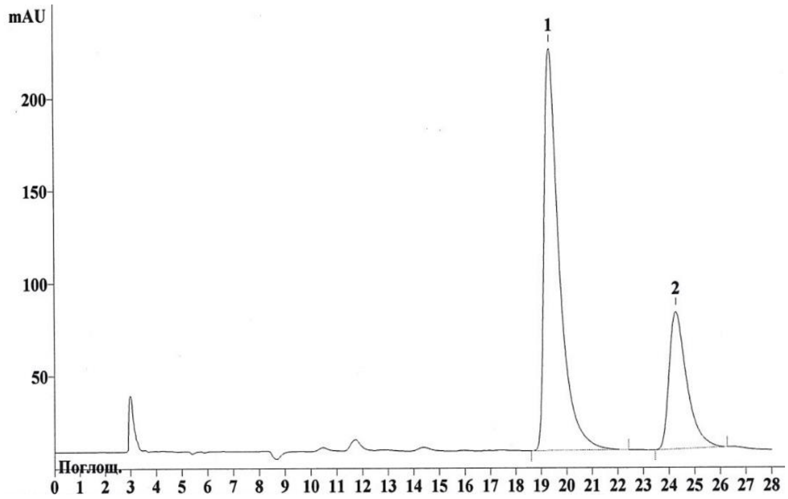

	Peak 1	Peak 2
Retention Time (min)	19.36	24.28
Relative Area (%)	71.63	28.37

Figure S20. HPLC chromatogram of **2a** after isolation (Entry 6).

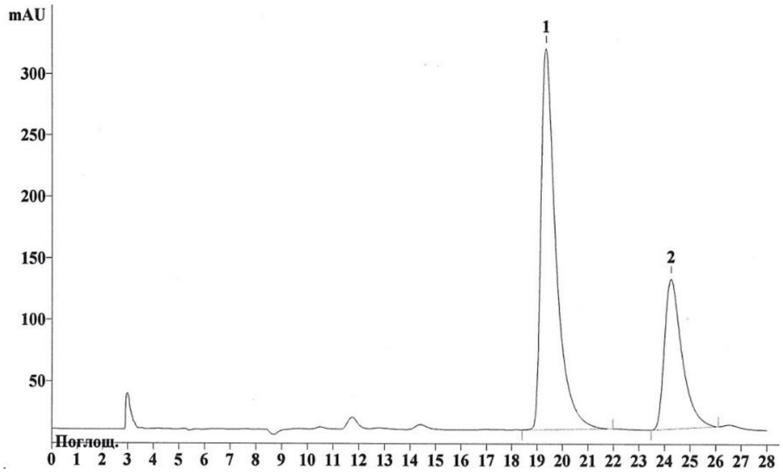

	Peak 1	Peak 2
Retention Time (min)	19.35	24.25
Relative Area (%)	68.66	31.34

Figure S21. HPLC chromatogram of **2a** after isolation (Entry 7).

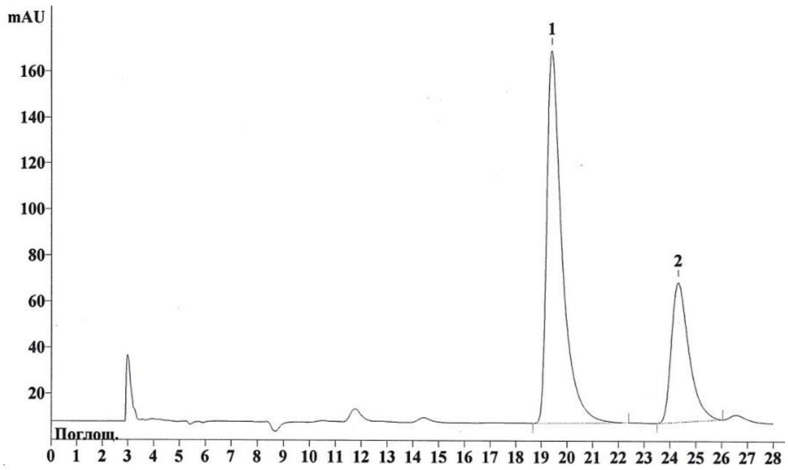

	Peak 1	Peak 2
Retention Time (min)	19.41	24.32
Relative Area (%)	70.03	29.97

Figure S22. HPLC chromatogram of **2a** after isolation (Entry 8).

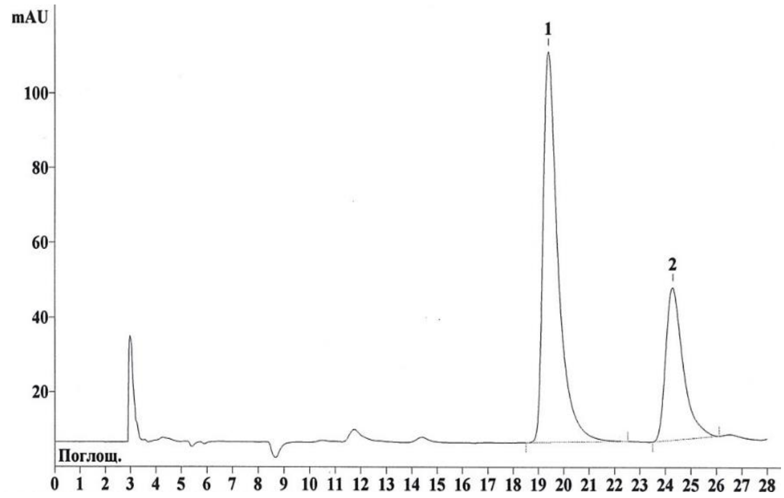

	Peak 1	Peak 2
Retention Time (min)	19.40	24.29
Relative Area (%)	69.02	30.98

Figure S23. HPLC chromatogram of **2a** after isolation (Entry 9).

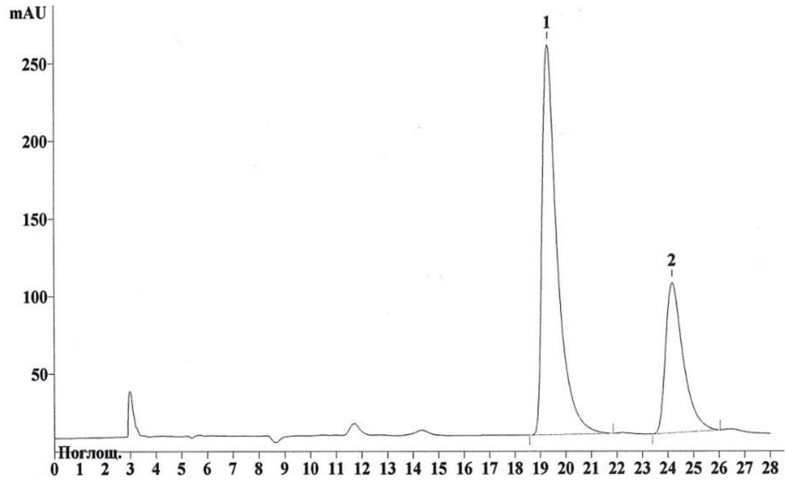

	Peak 1	Peak 2
Retention Time (min)	19.30	24.19
Relative Area (%)	69.09	30.91

Figure S24. HPLC chromatogram of **2a** after isolation (Entry 10).

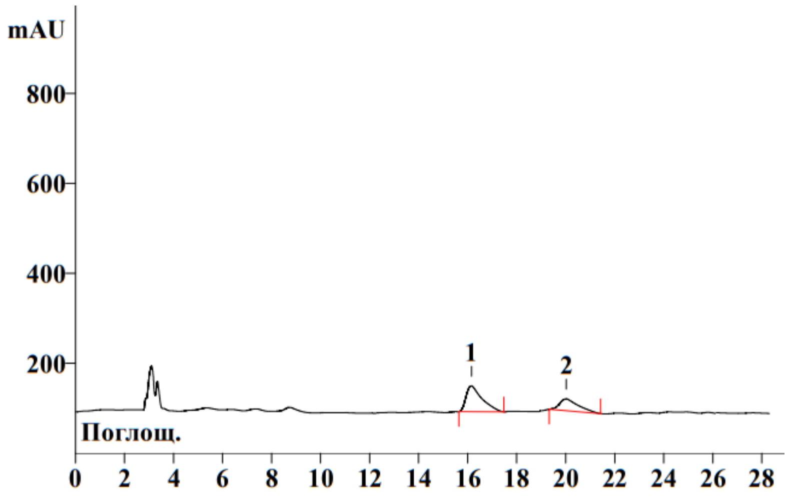

	Peak 1	Peak 2
Retention Time (min)	16.15	20.03
Relative Area (%)	64.84	35.16

Figure S25. HPLC chromatogram of **2a** after isolation (Entry 11).

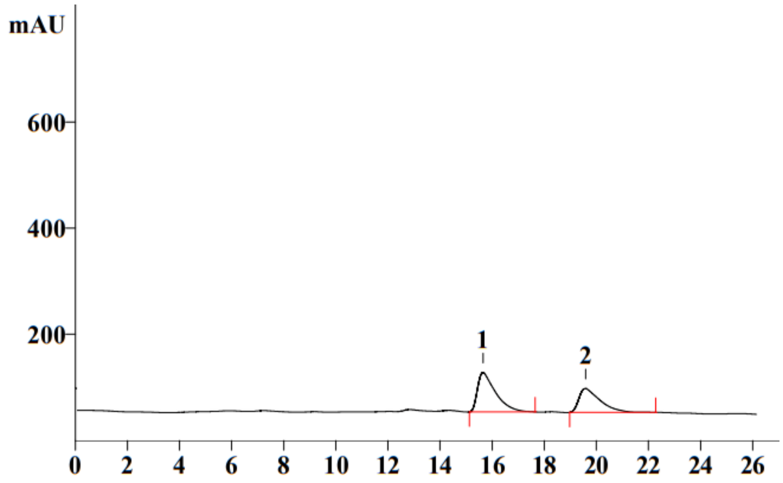

	Peak 1	Peak 2
Retention Time (min)	15.65	19.68
Relative Area (%)	58.23	41.77

Figure S26. HPLC chromatogram of **2a** after isolation (Entry 12).

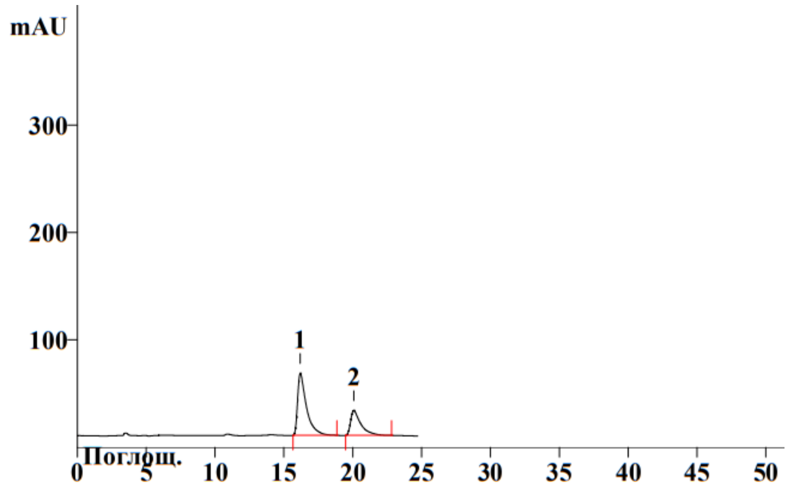

	Peak 1	Peak 2
Retention Time (min)	16.18	20.07
Relative Area (%)	68.43	31.57

Figure S27. HPLC chromatogram of **2a** after isolation (Entry 13).

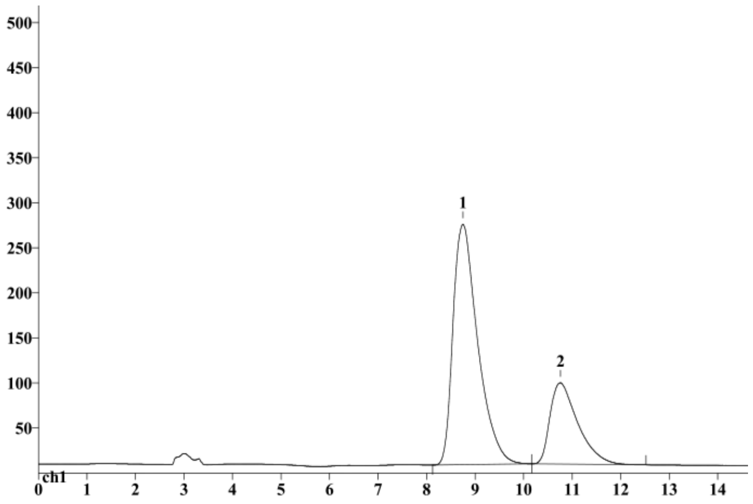

	Peak 1	Peak 2
Retention Time (min)	8.75	10.75
Relative Area (%)	58.23	41.77

Figure S28. HPLC chromatogram of **2b** after isolation (Entry 14).

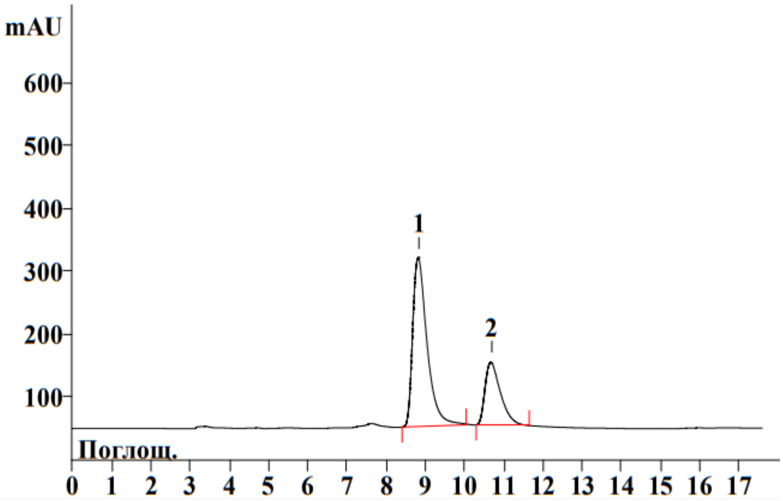

	Peak 1	Peak 2
Retention Time (min)	8.82	10.67
Relative Area (%)	71.16	28.84

Figure S29. HPLC chromatogram of **2b** after isolation (Entry 15).

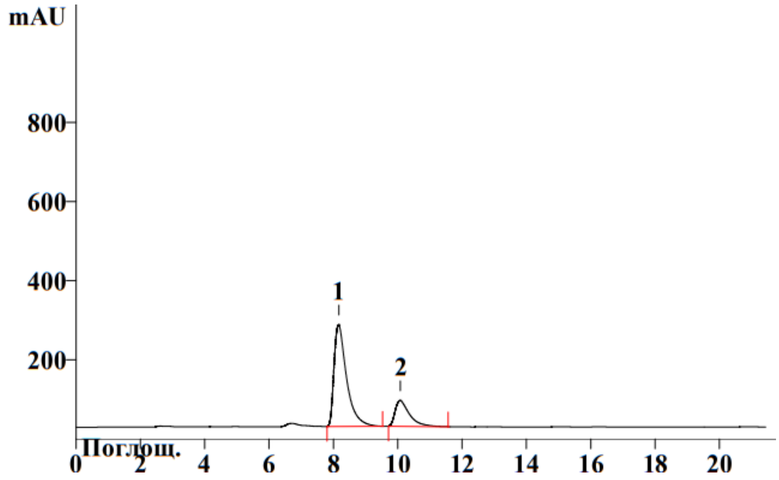

	Peak 1	Peak 2
Retention Time (min)	8.16	10.08
Relative Area (%)	77.57	22.03

Figure S30. HPLC chromatogram of **2b** after isolation (Entry 16).

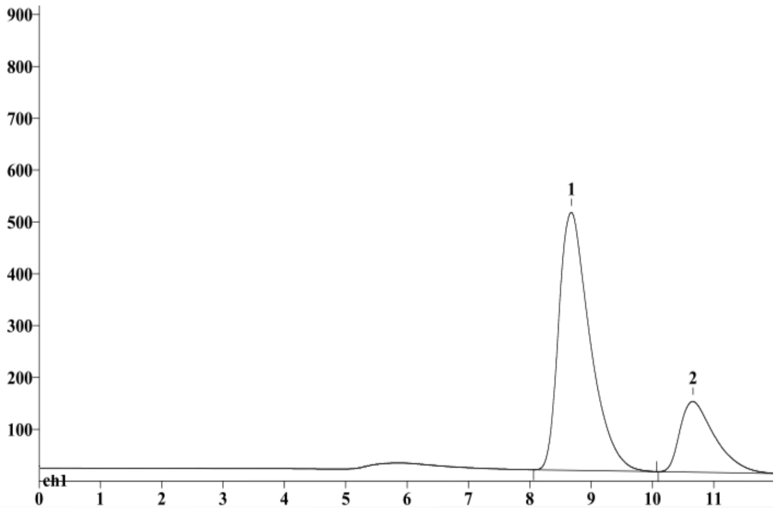

	Peak 1	Peak 2
Retention Time (min)	8.67	10.65
Relative Area (%)	76.55	23.45

Figure S31. HPLC chromatogram of **2b** after isolation (Entry 17).

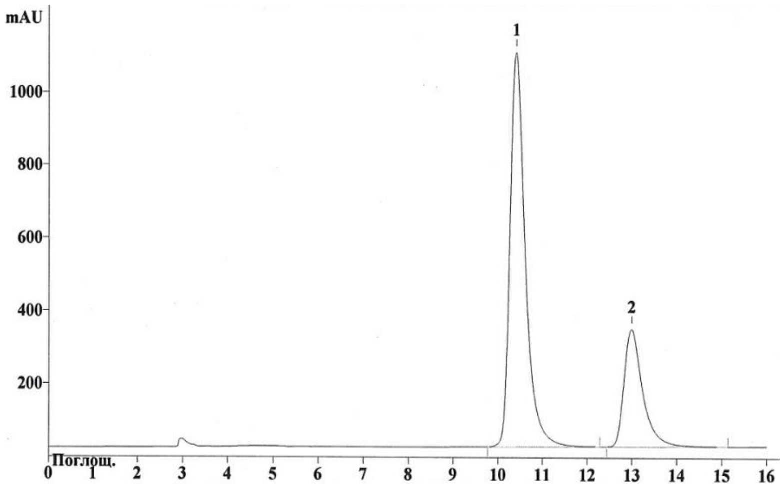

	Peak 1	Peak 2
Retention Time (min)	10.41	12.99
Relative Area (%)	73.49	26.51

Figure S33. HPLC chromatogram of **2b** after isolation (Entry 18).

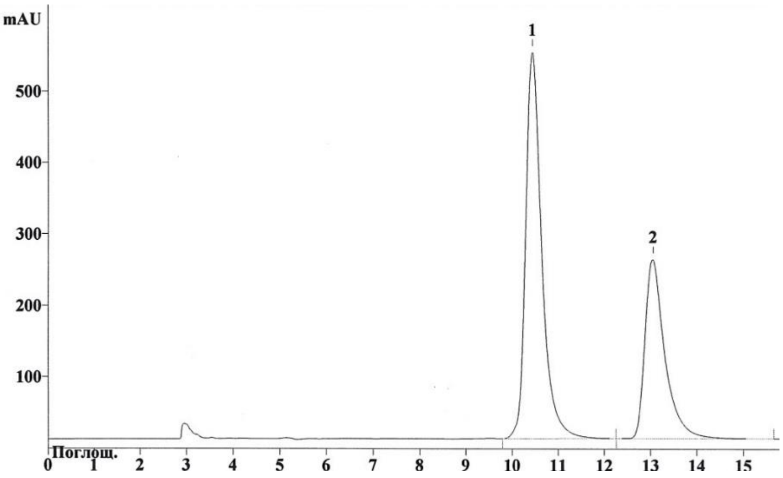

	Peak 1	Peak 2
Retention Time (min)	10.45	13.05
Relative Area (%)	63.85	36.15

Figure S33. HPLC chromatogram of **2b** after isolation (Entry 19).

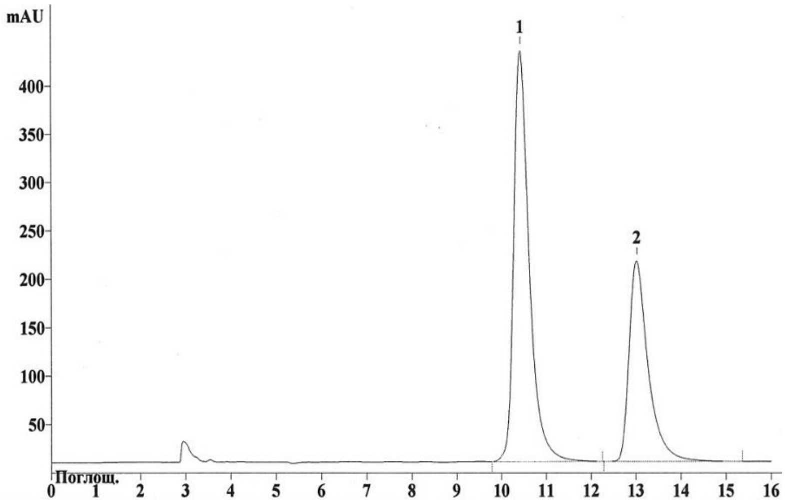

	Peak 1	Peak 2
Retention Time (min)	10.43	13.02
Relative Area (%)	62.92	37.08

Figure S34. HPLC chromatogram of **2b** after isolation (Entry 20).

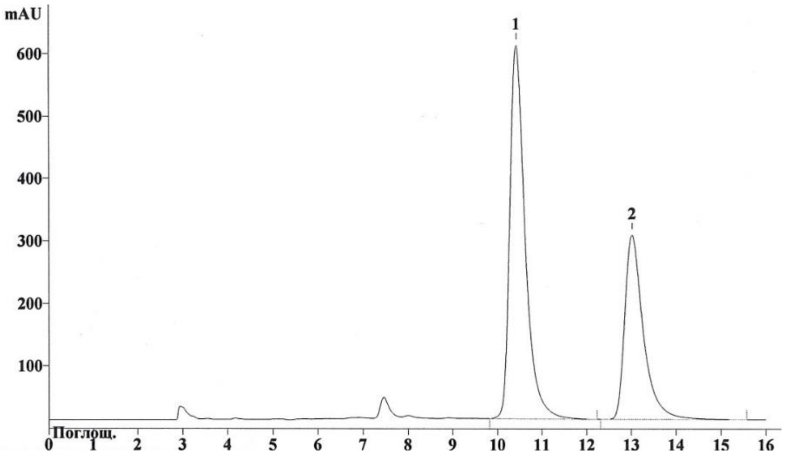

	Peak 1	Peak 2
Retention Time (min)	10.43	13.03
Relative Area (%)	62.82	37.18

Figure S35. HPLC chromatogram of **2b** after isolation (Entry 21).

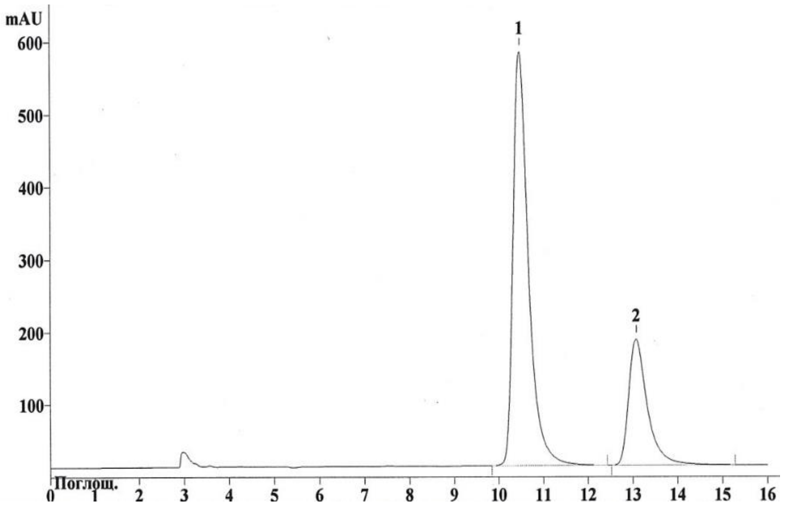

	Peak 1	Peak 2
Retention Time (min)	10.48	13.08
Relative Area (%)	73.36	26.64

Figure S36. HPLC chromatogram of **2b** after isolation (Entry 22).

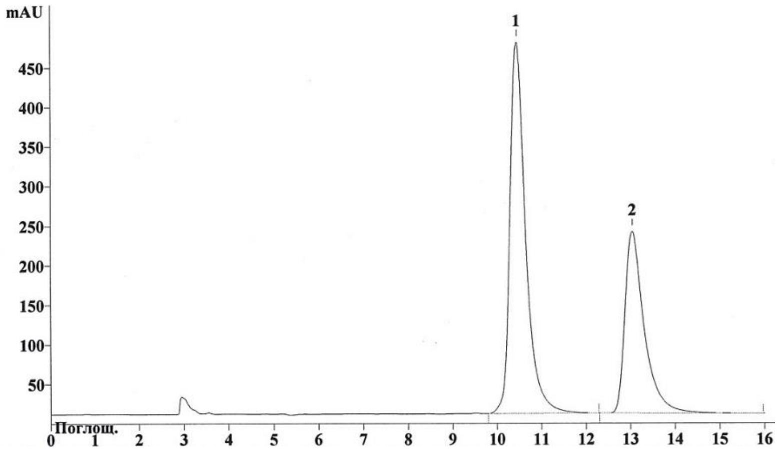

	Peak 1	Peak 2
Retention Time (min)	10.45	13.05
Relative Area (%)	62.62	37.38

Figure S37. HPLC chromatogram of **2b** after isolation (Entry 23).

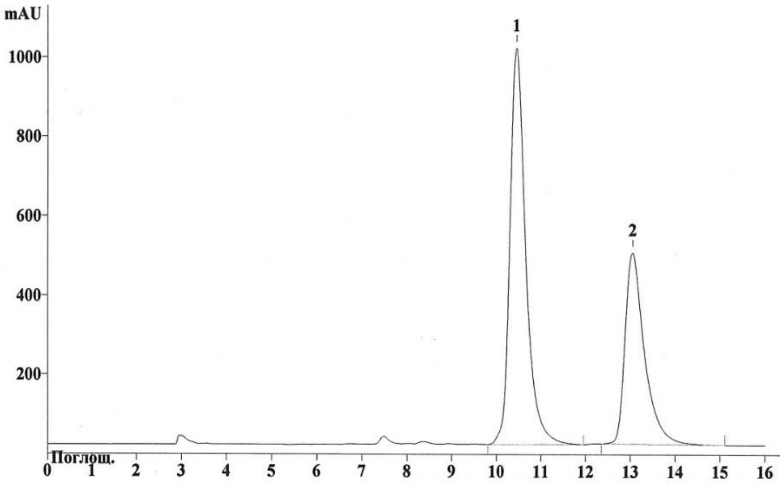

	Peak 1	Peak 2
Retention Time (min)	10.46	13.06
Relative Area (%)	62.98	37.02

Figure S38. HPLC chromatogram of **2b** after isolation (Entry 24).

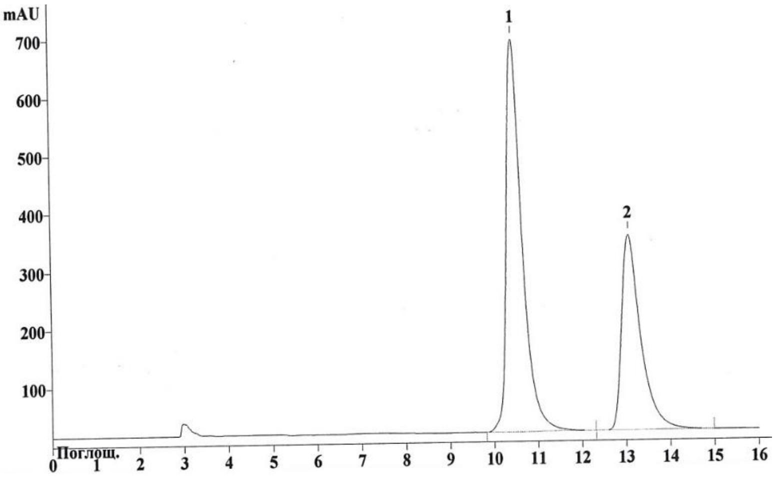

	Peak 1	Peak 2
Retention Time (min)	10.48	13.09
Relative Area (%)	62.69	37.31

Figure S39. HPLC chromatogram of **2b** after isolation (Entry 25).

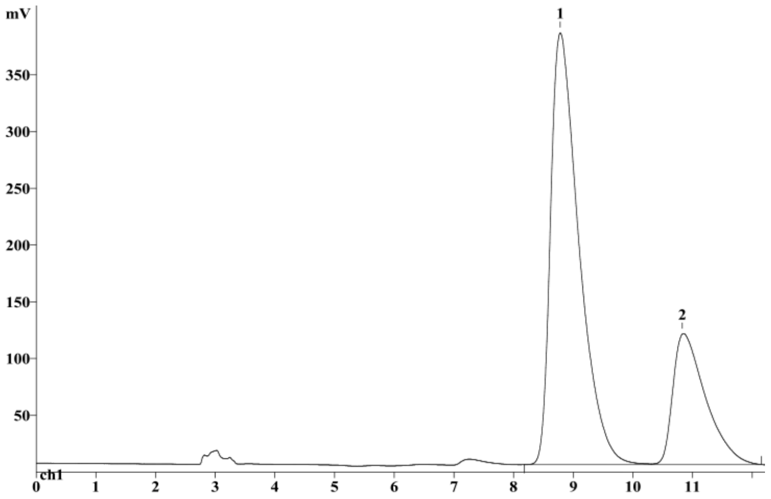

	Peak 1	Peak 2
Retention Time (min)	8.78	10.85
Relative Area (%)	74.15	25.85

Figure S40. HPLC chromatogram of **2b** after isolation (Entry 26).

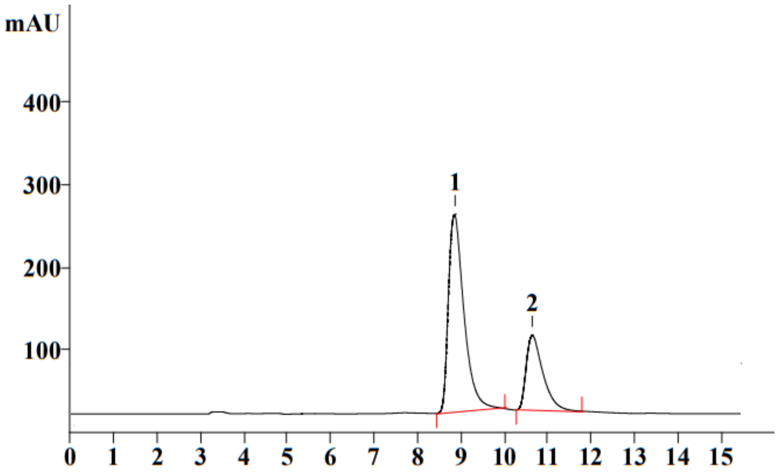

	Peak 1	Peak 2
Retention Time (min)	8.86	10.55
Relative Area (%)	71.09	28.91

Figure S41. HPLC chromatogram of **2b** after isolation (Entry 27).

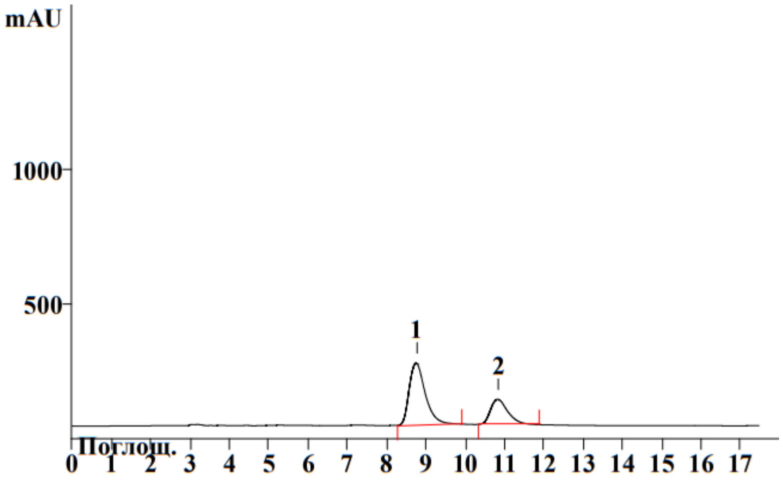

	Peak 1	Peak 2
Retention Time (min)	8.76	10.83
Relative Area (%)	69.51	30.49

Figure S42. HPLC chromatogram of **2b** after isolation (Entry 28).

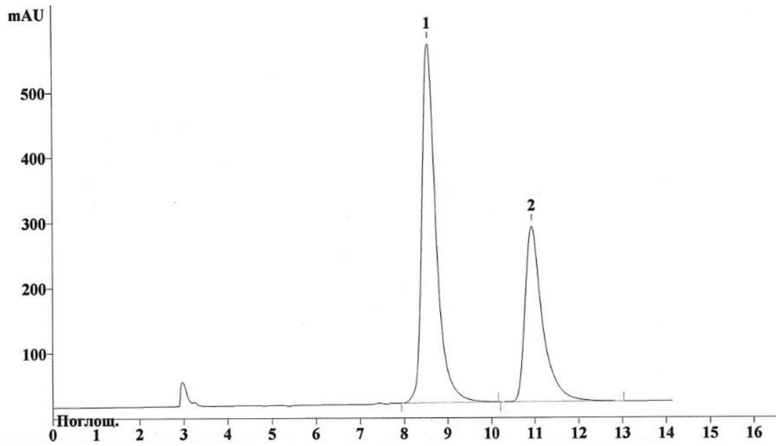

	Peak 1	Peak 2
Retention Time (min)	8.91	11.03
Relative Area (%)	62.61	37.39

Figure S43. HPLC chromatogram of **2b** after isolation (Entry 29).

4. NMR spectra of newly formed alcohols 2a, 2b (Table 4)

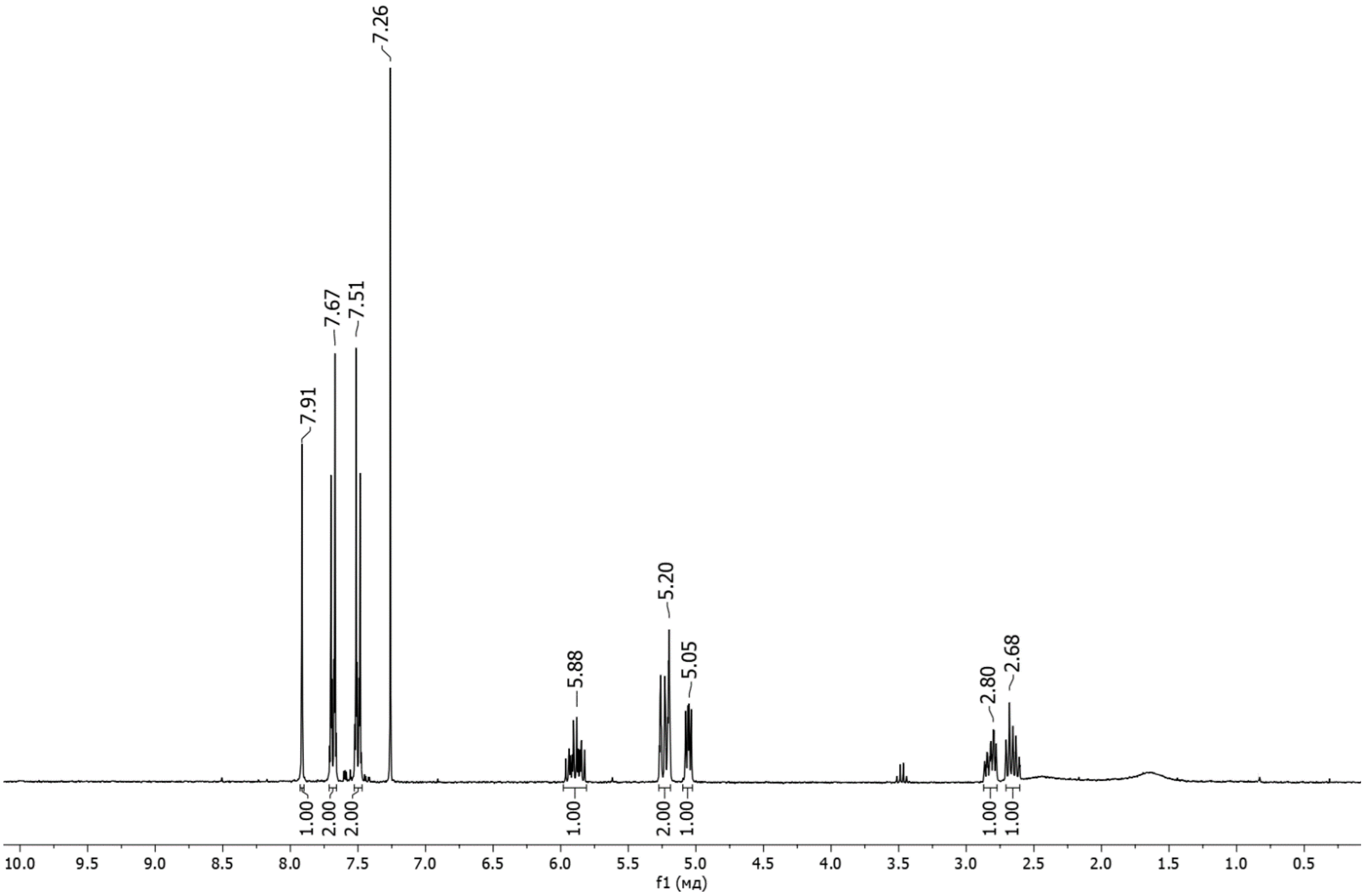

Figure S44. NMR spectra of **2a** after isolation (Entry 1).

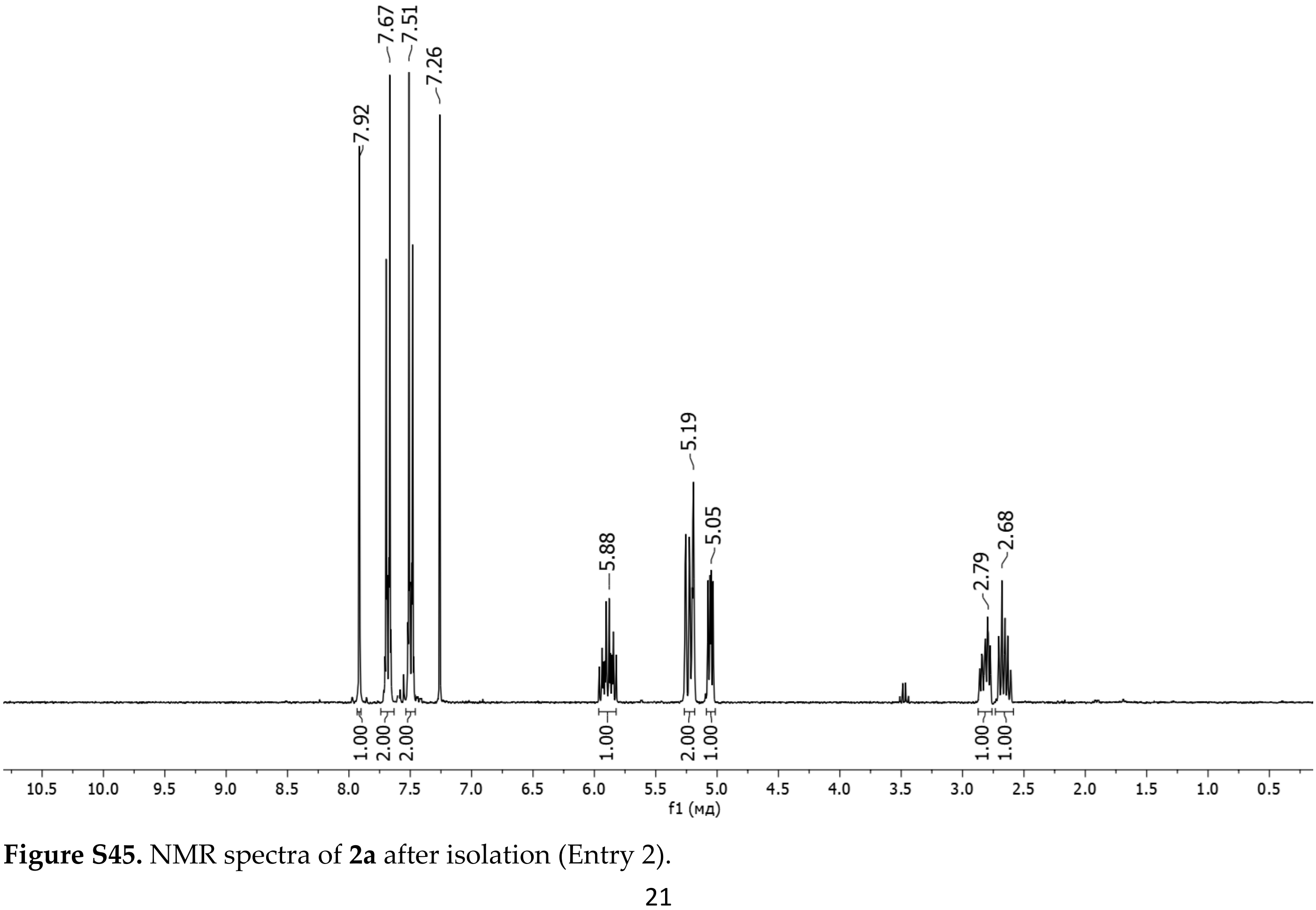

Figure S45. NMR spectra of **2a** after isolation (Entry 2).

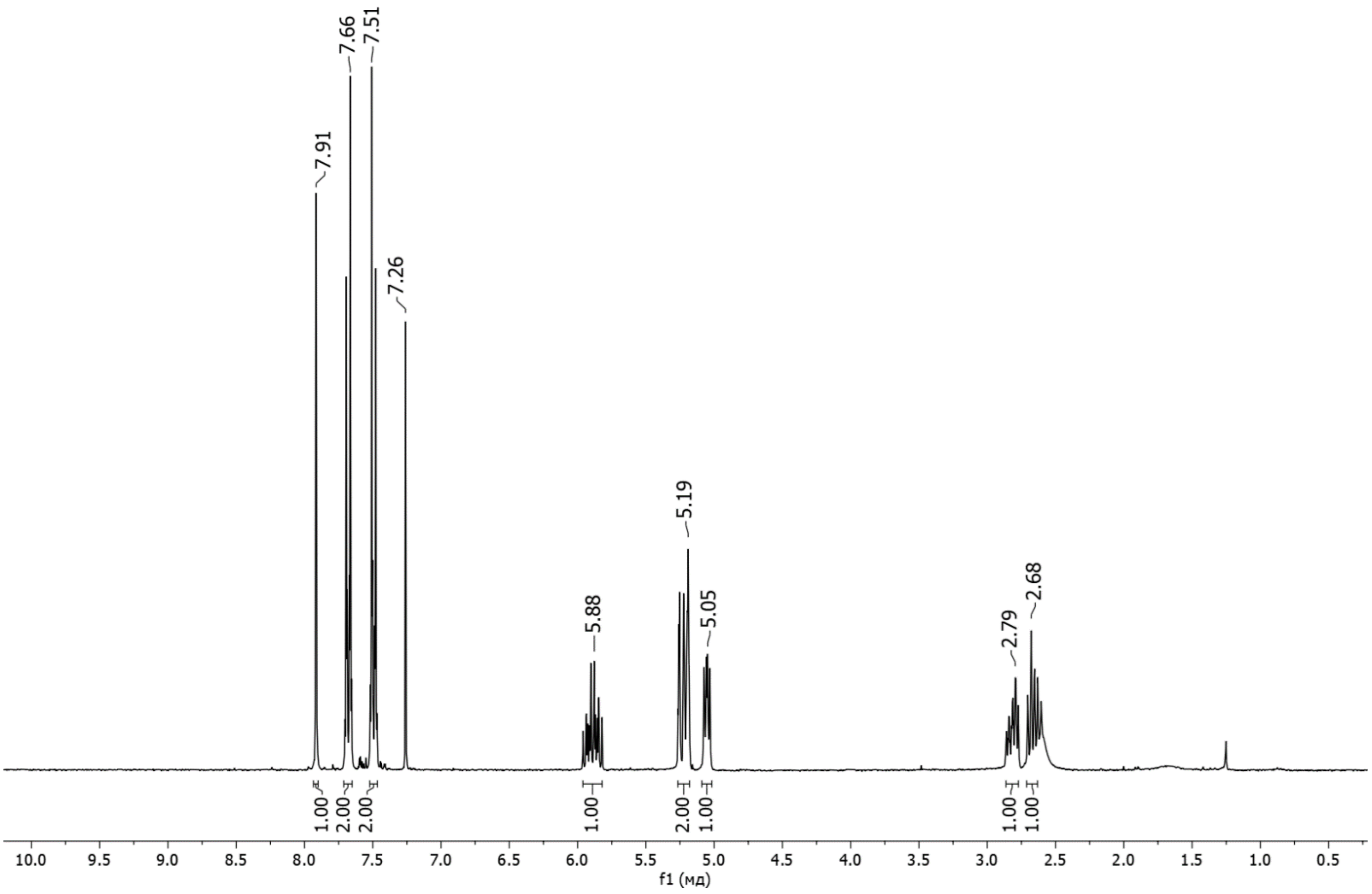

Figure S46. NMR spectra of **2a** after isolation (Entry 3).

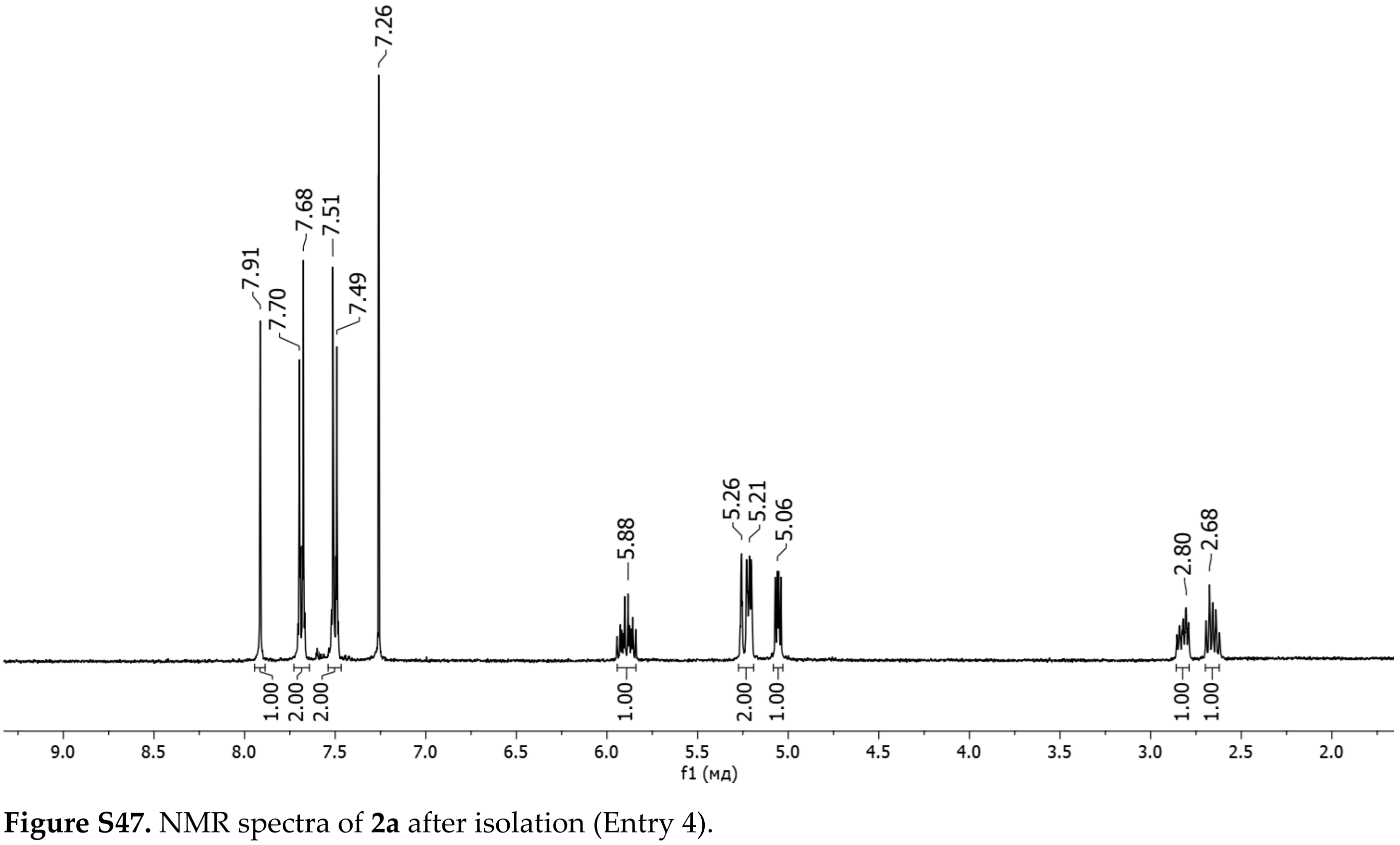

Figure S47. NMR spectra of **2a** after isolation (Entry 4).

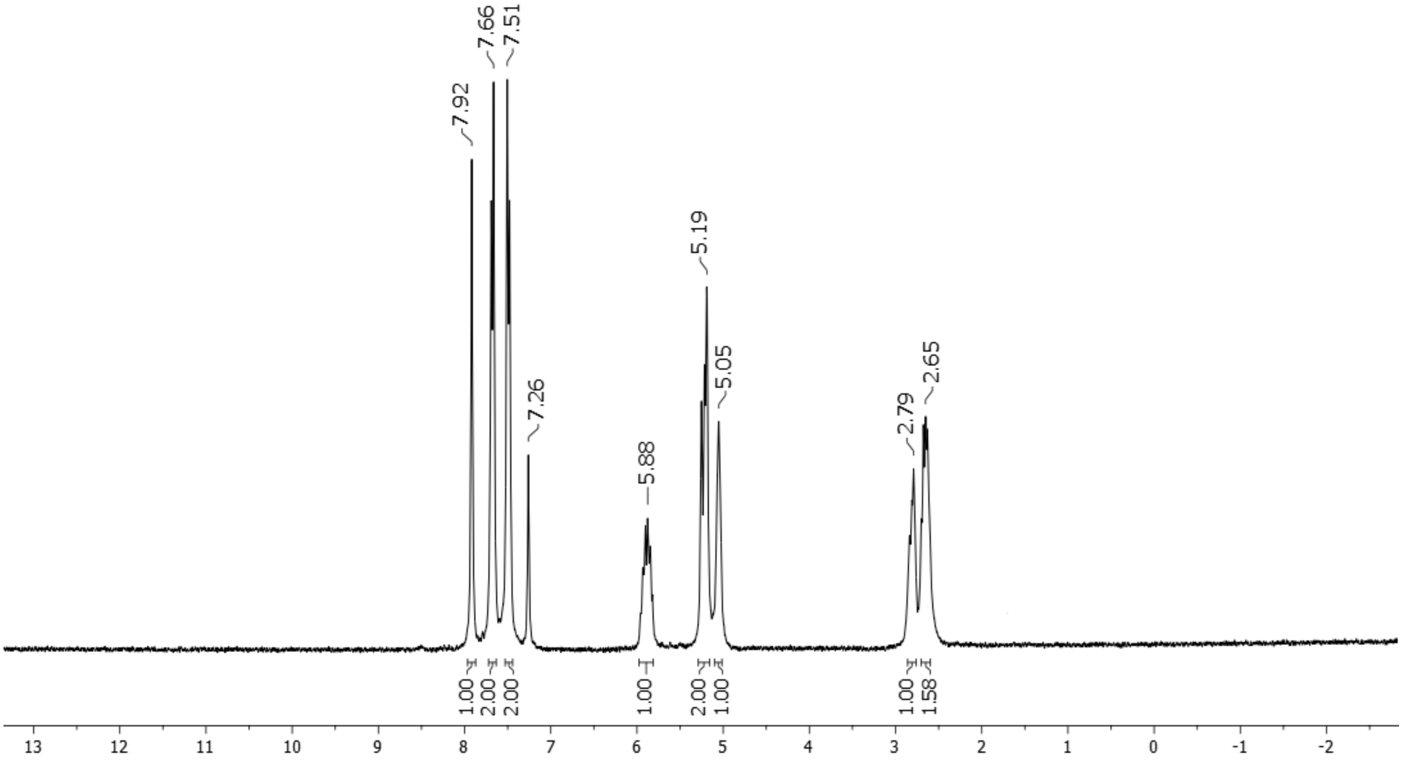

Figure S48. NMR spectra of **2a** after isolation (Entry 5).

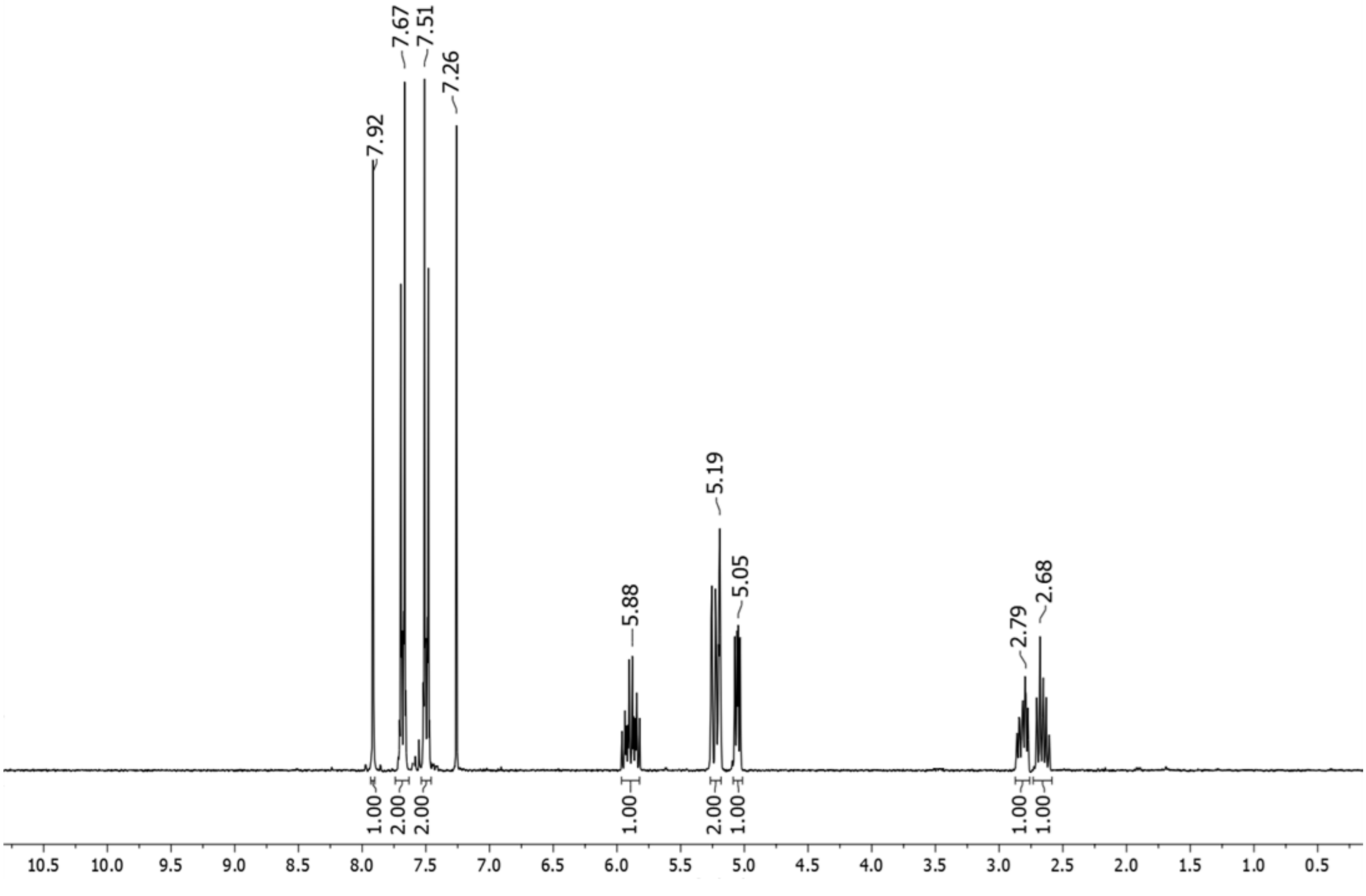

Figure S49. NMR spectra of **2a** after isolation (Entry 6).

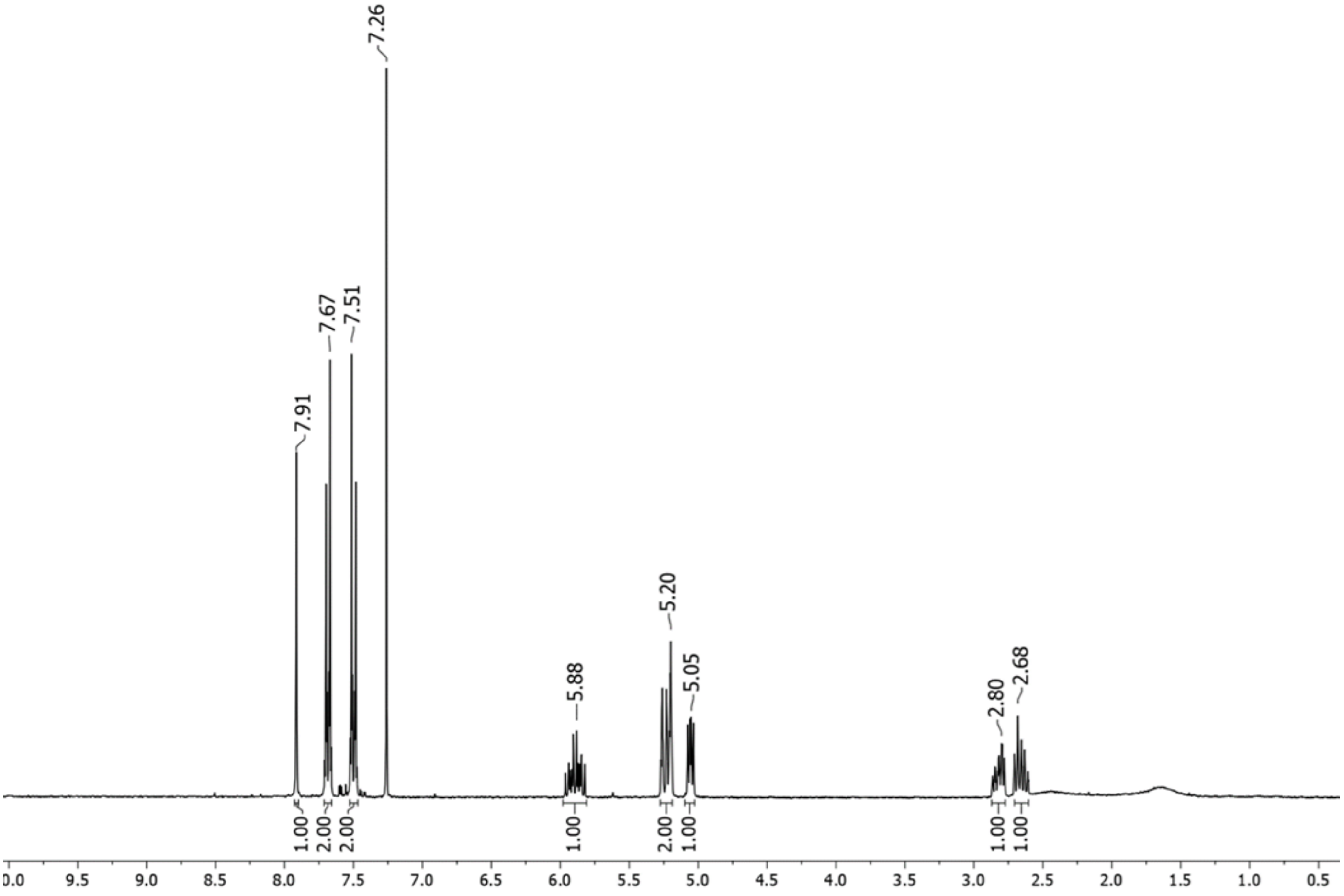

Figure S50. NMR spectra of **2a** after isolation (Entry 7).

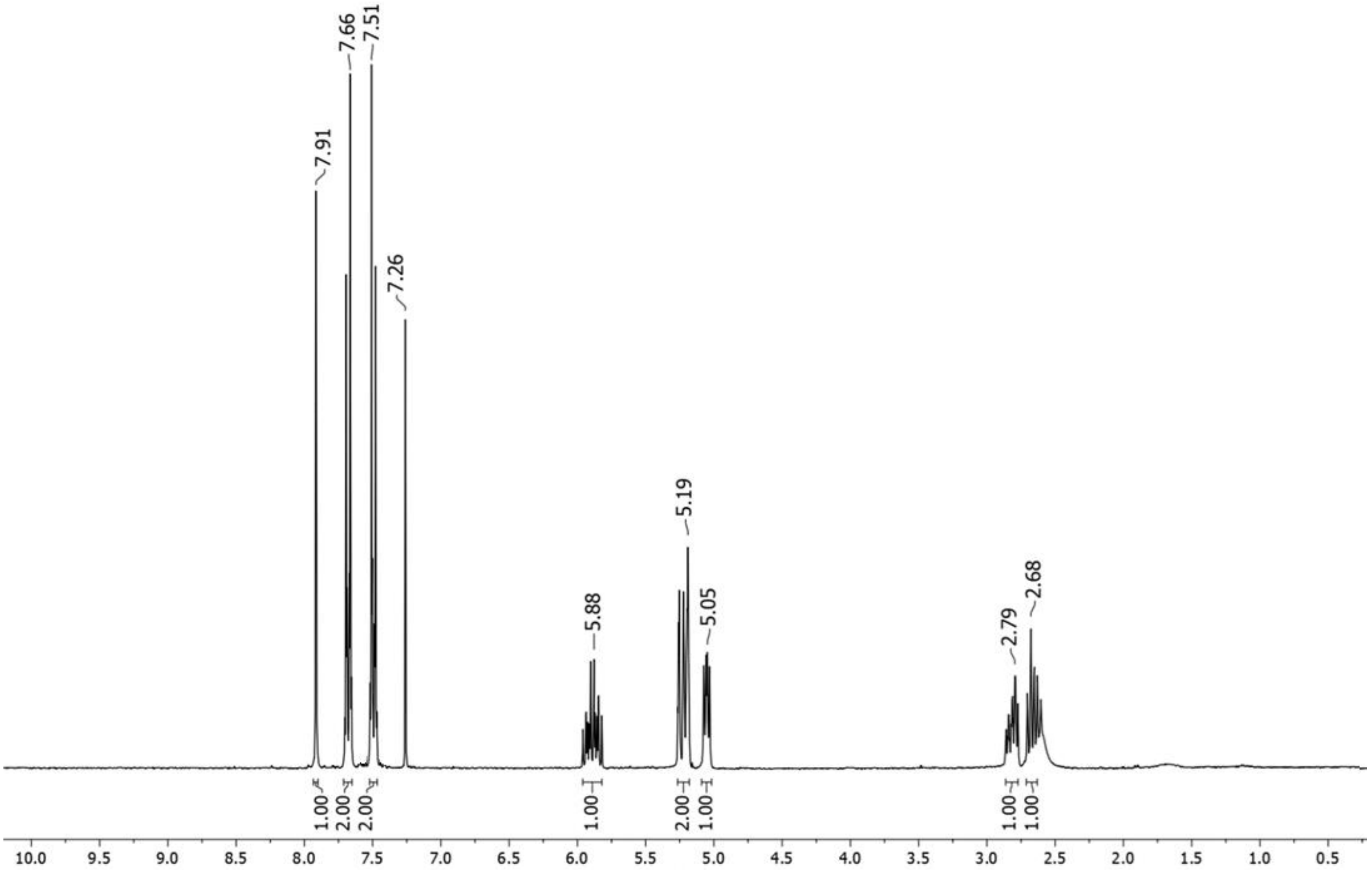

Figure S51. NMR spectra of **2a** after isolation (Entry 8).

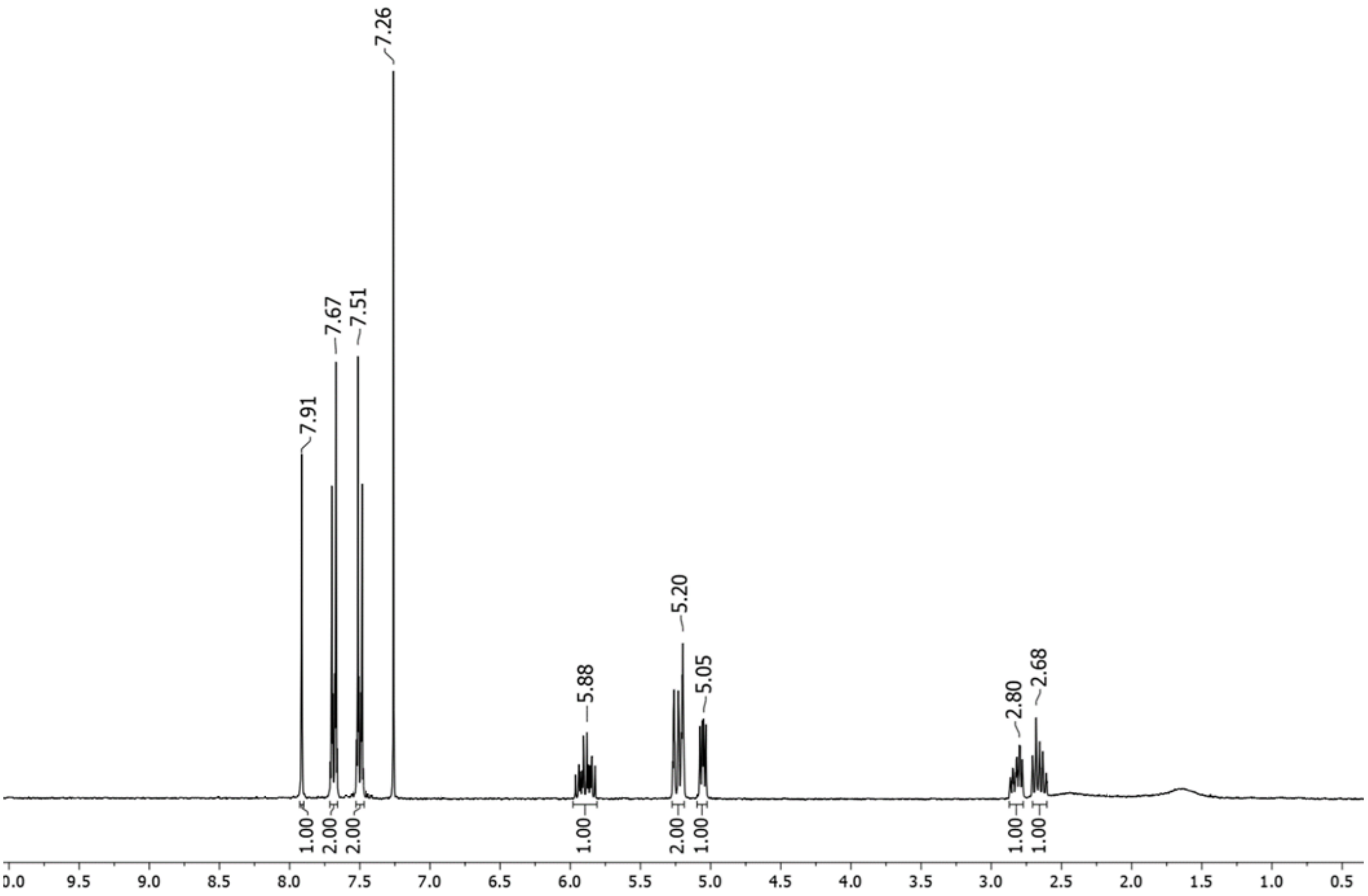

Figure S52. NMR spectra of **2a** after isolation (Entry 9).

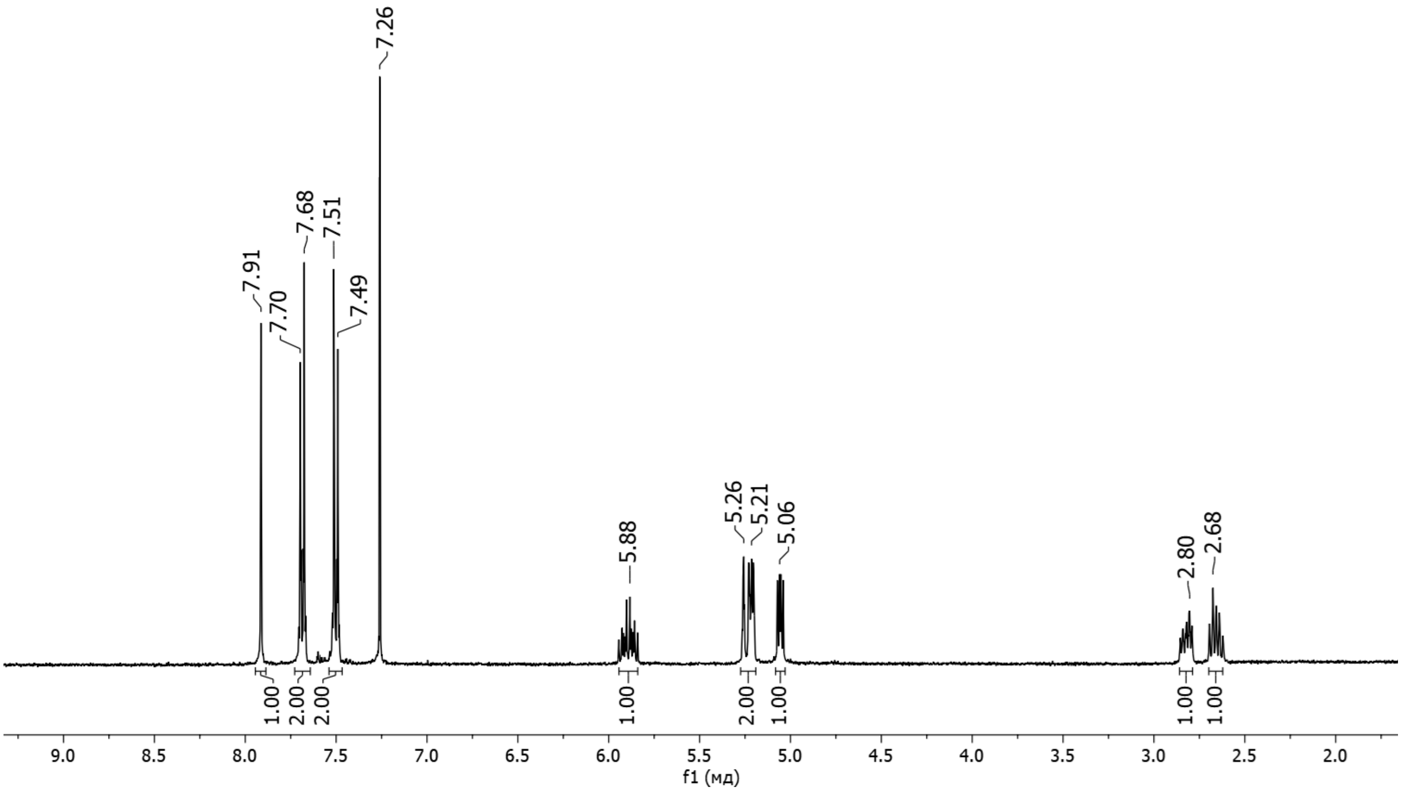

Figure S53. NMR spectra of **2a** after isolation (Entry 10).

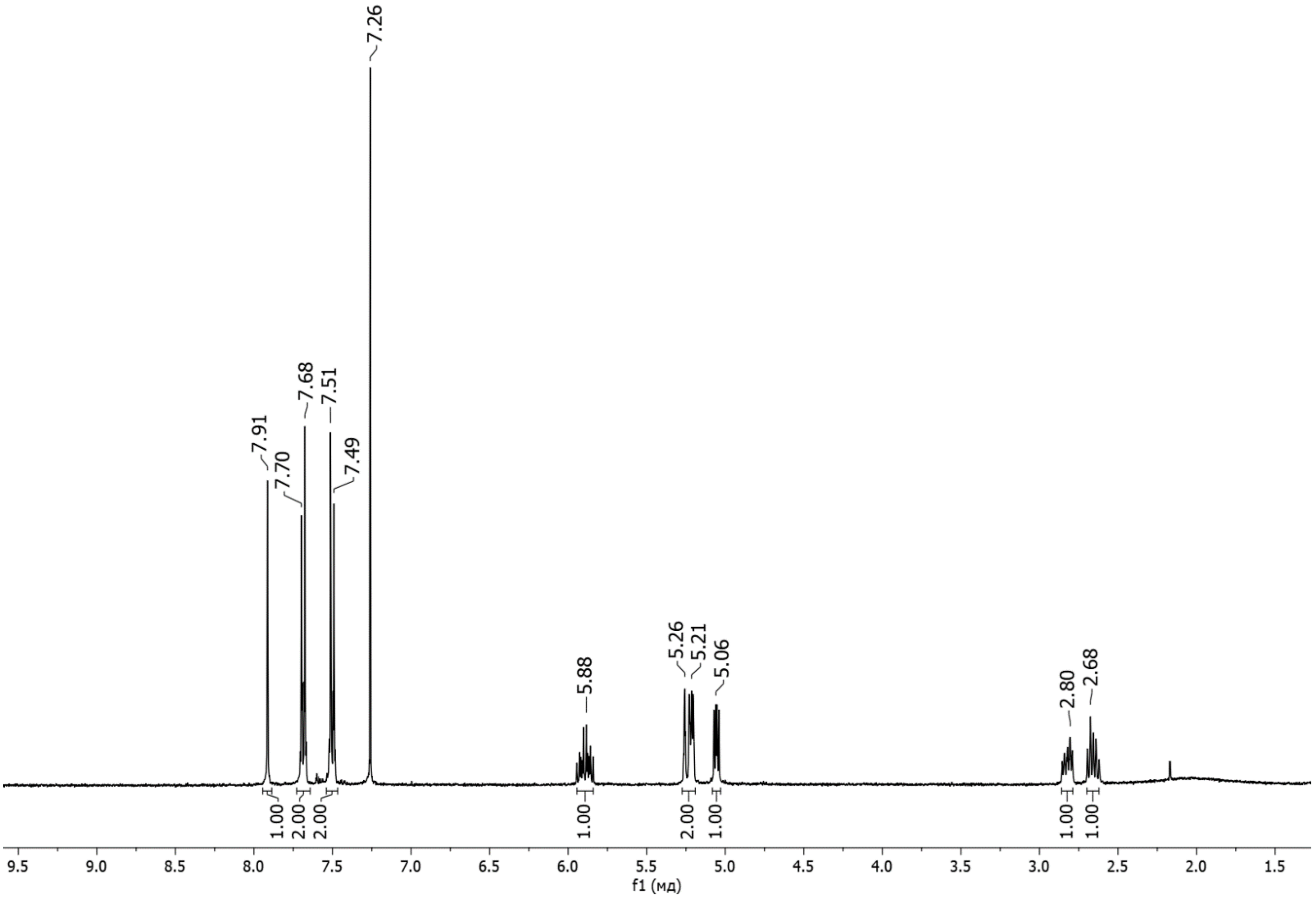

Figure S54. NMR spectra of **2a** after isolation (Entry 11).

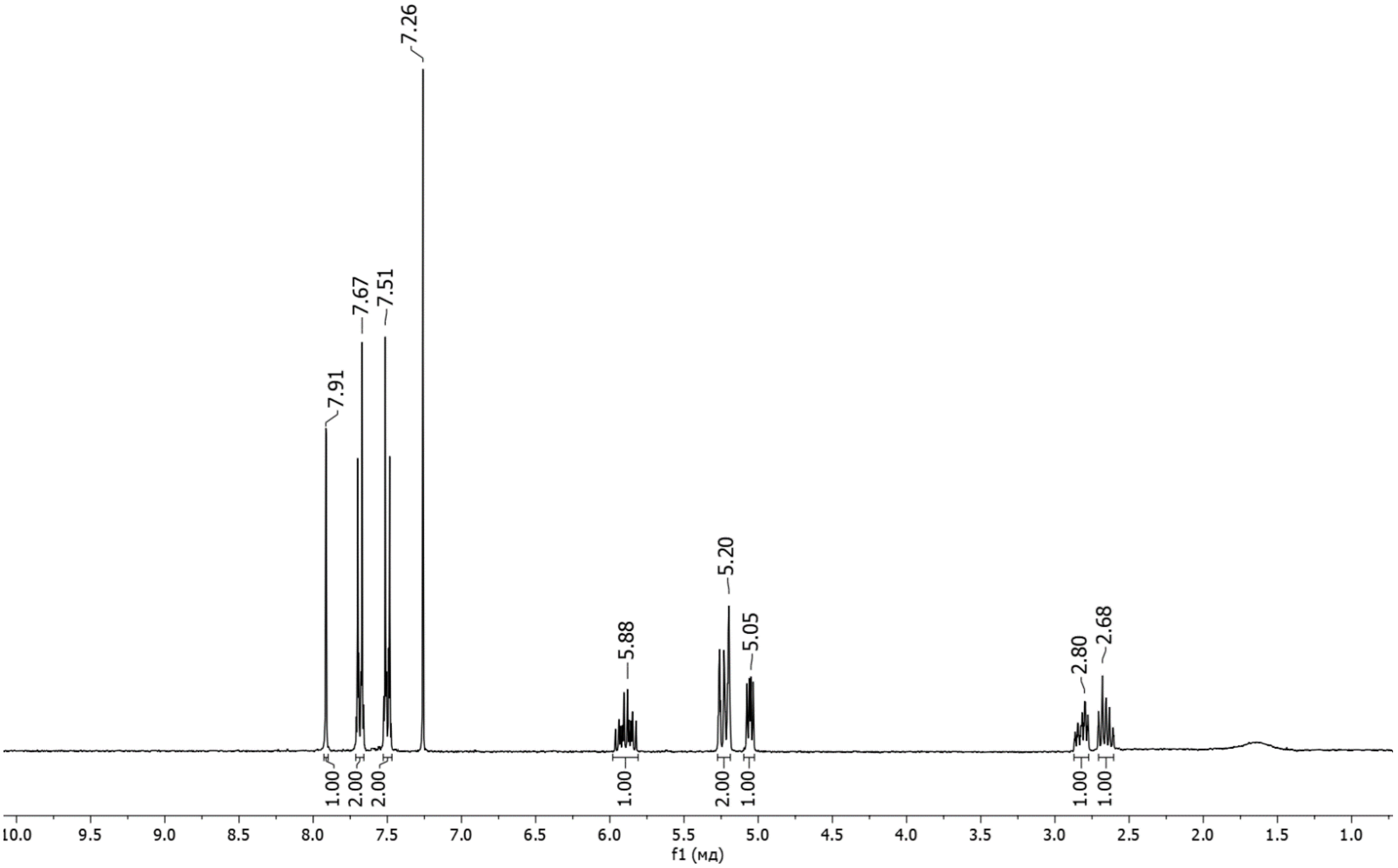

Figure S55. NMR spectra of **2a** after isolation (Entry 12).

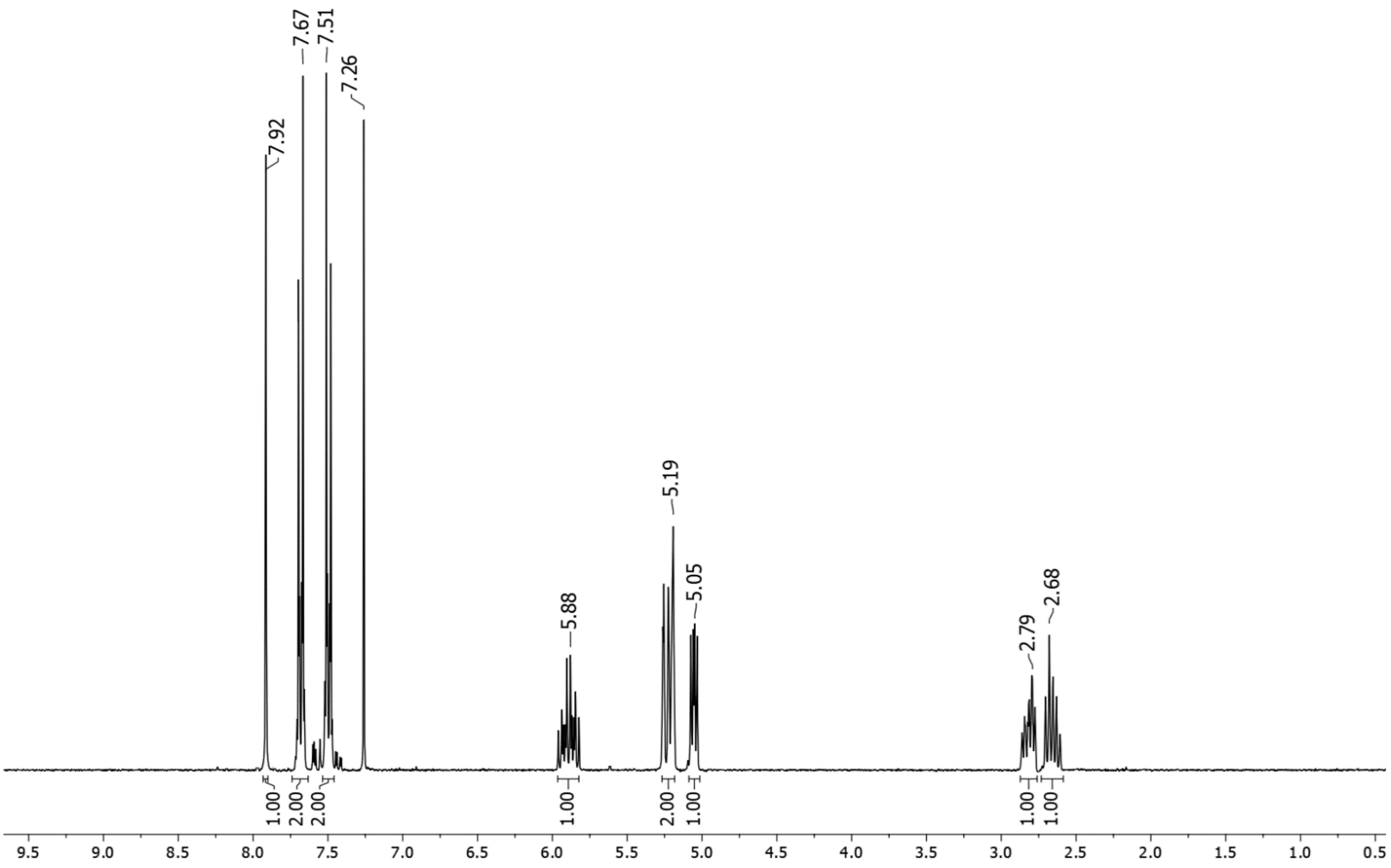

Figure S56. NMR spectra of **2a** after isolation (Entry 13).

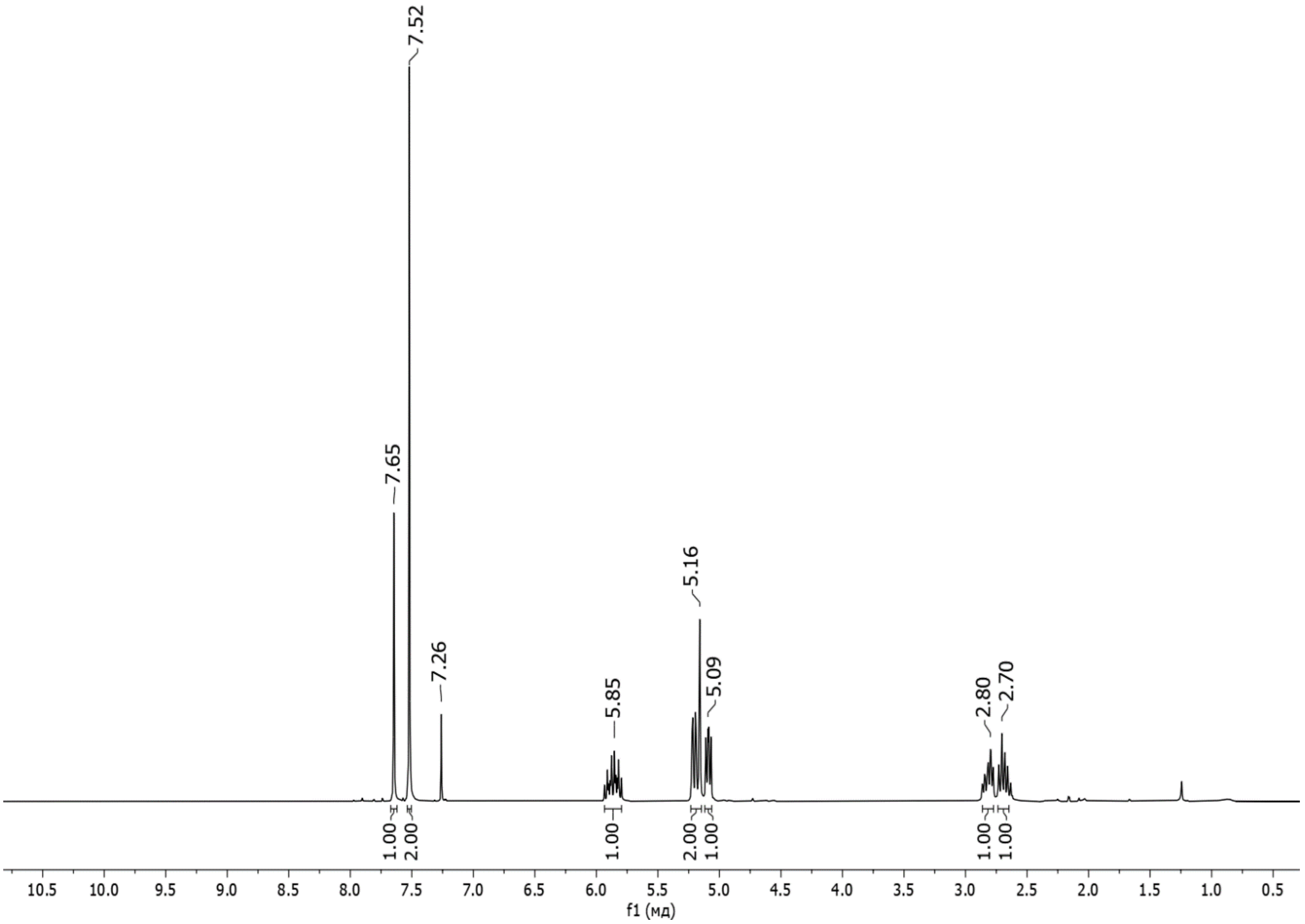

Figure S57. NMR spectra of **2b** after isolation (Entry 14).

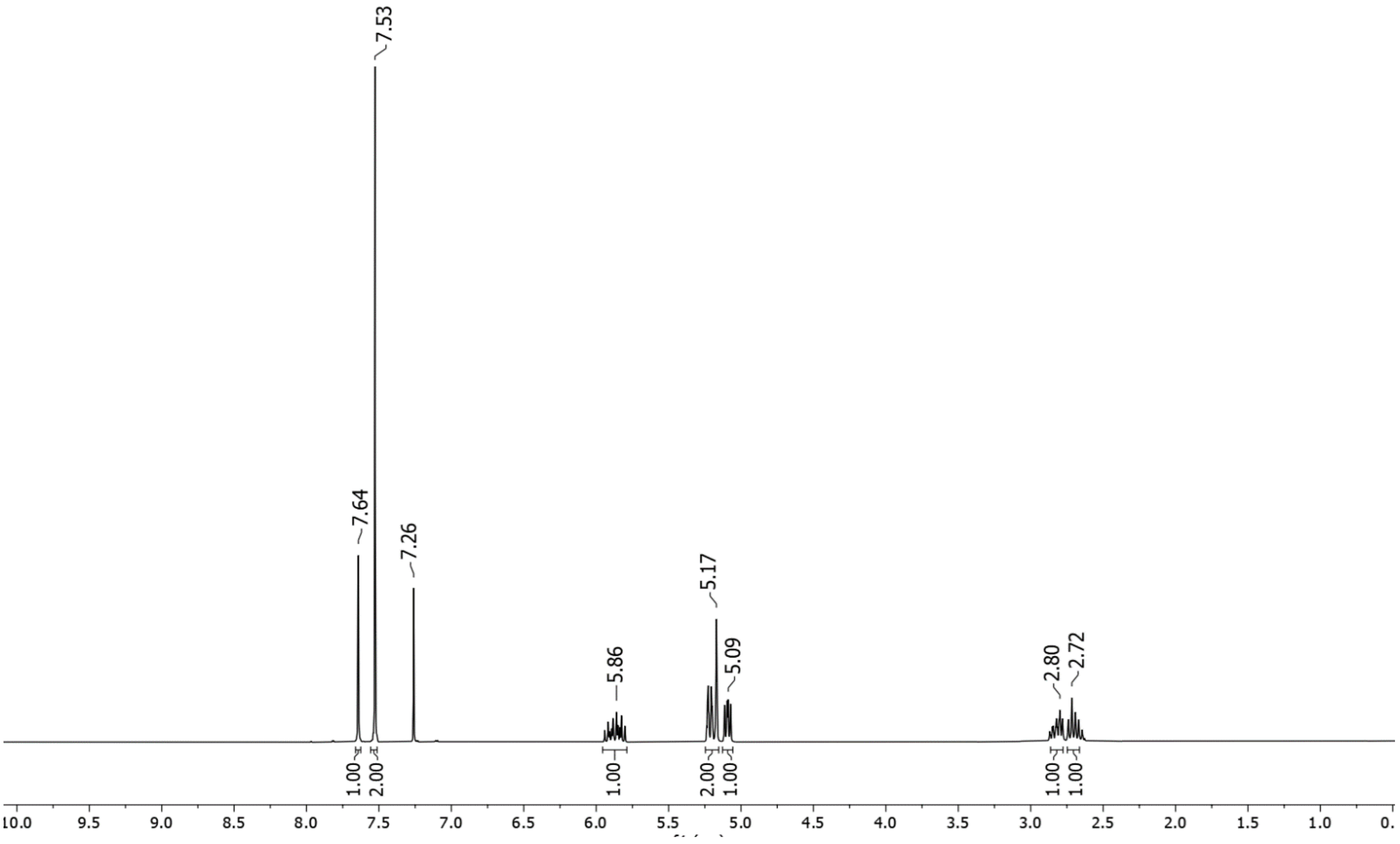

Figure S58. NMR spectra of **2b** after isolation (Entry 15).

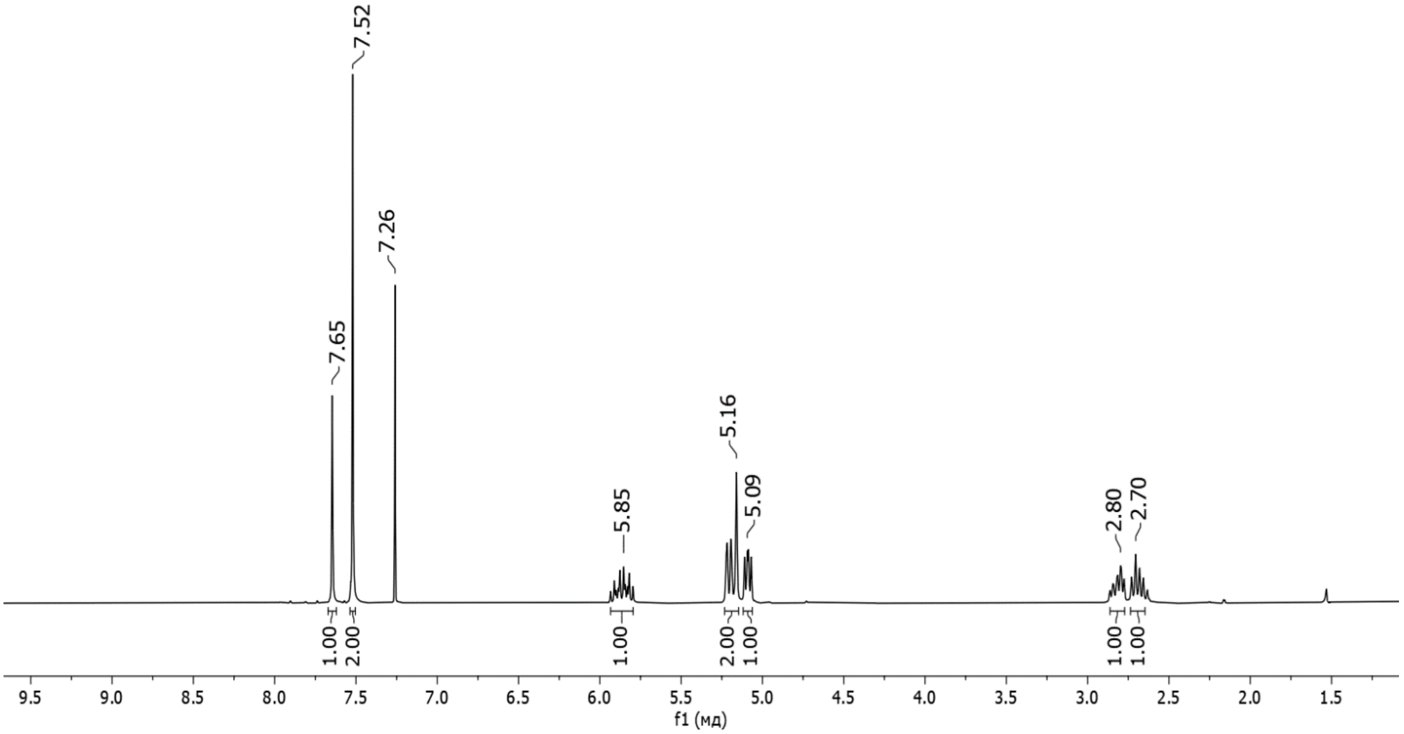

Figure S59. NMR spectra of **2b** after isolation (Entry 16).

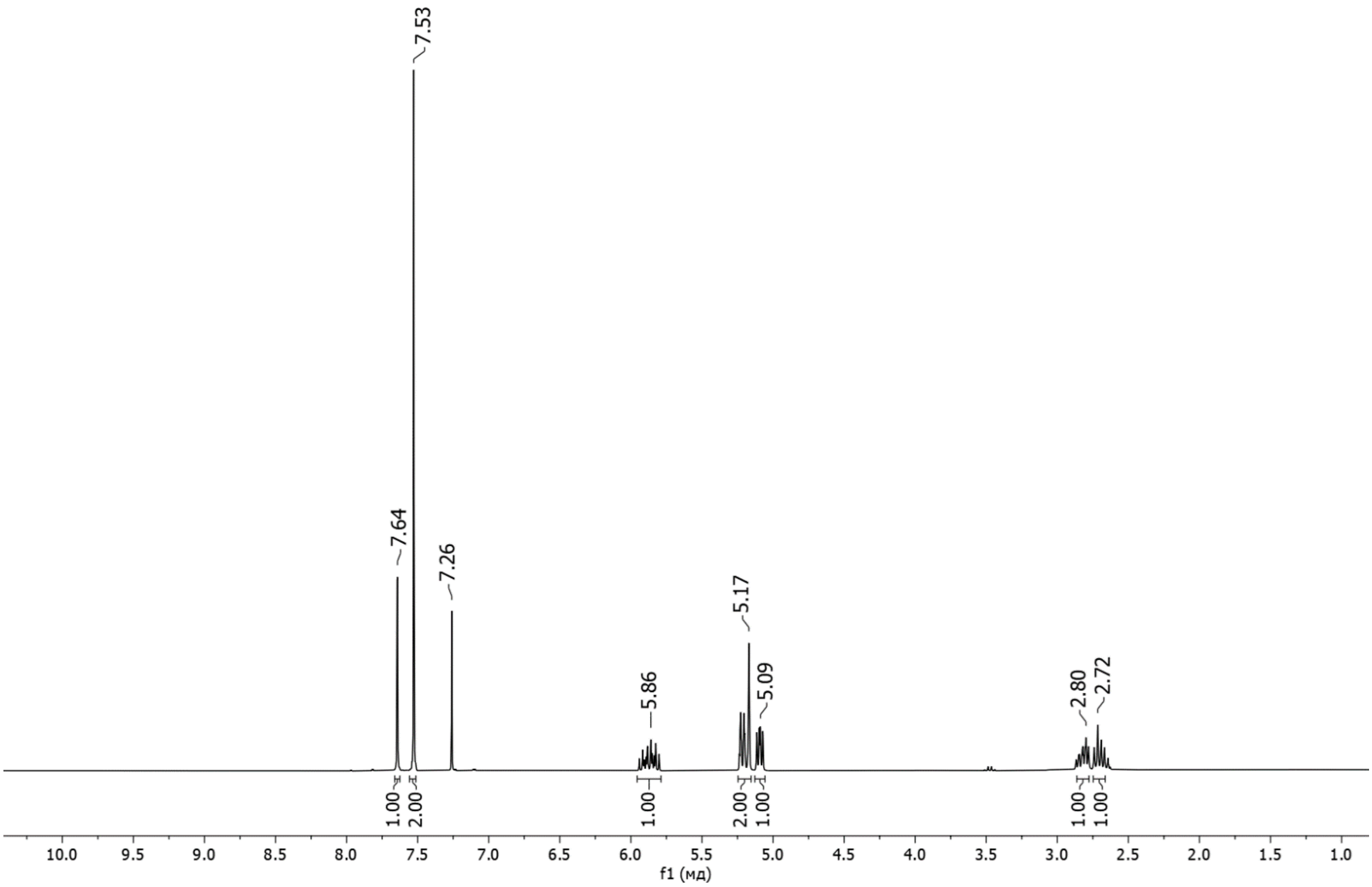

Figure S60. NMR spectra of **2b** after isolation (Entry 17).

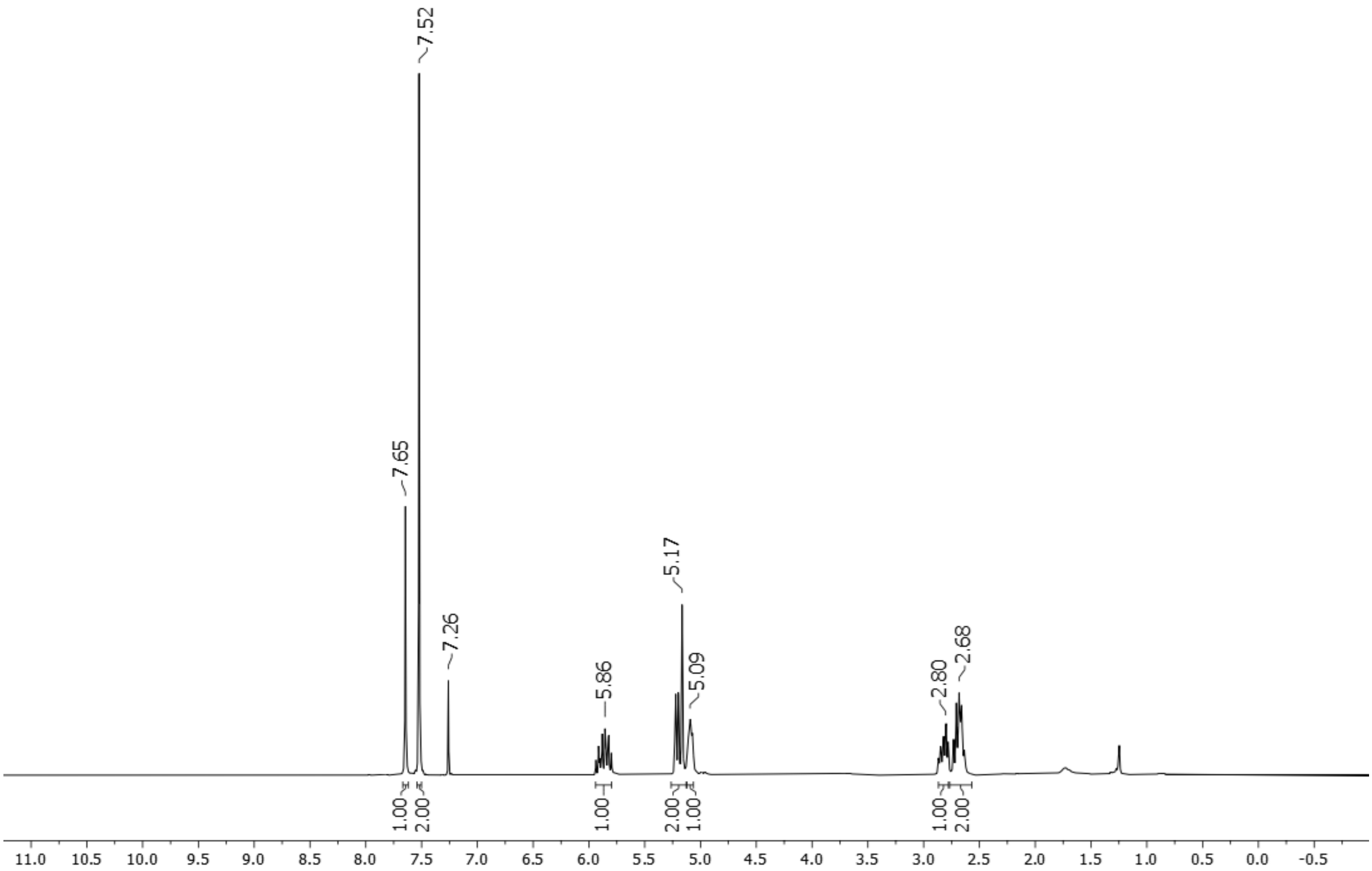

Figure S61. NMR spectra of **2b** after isolation (Entry 18).

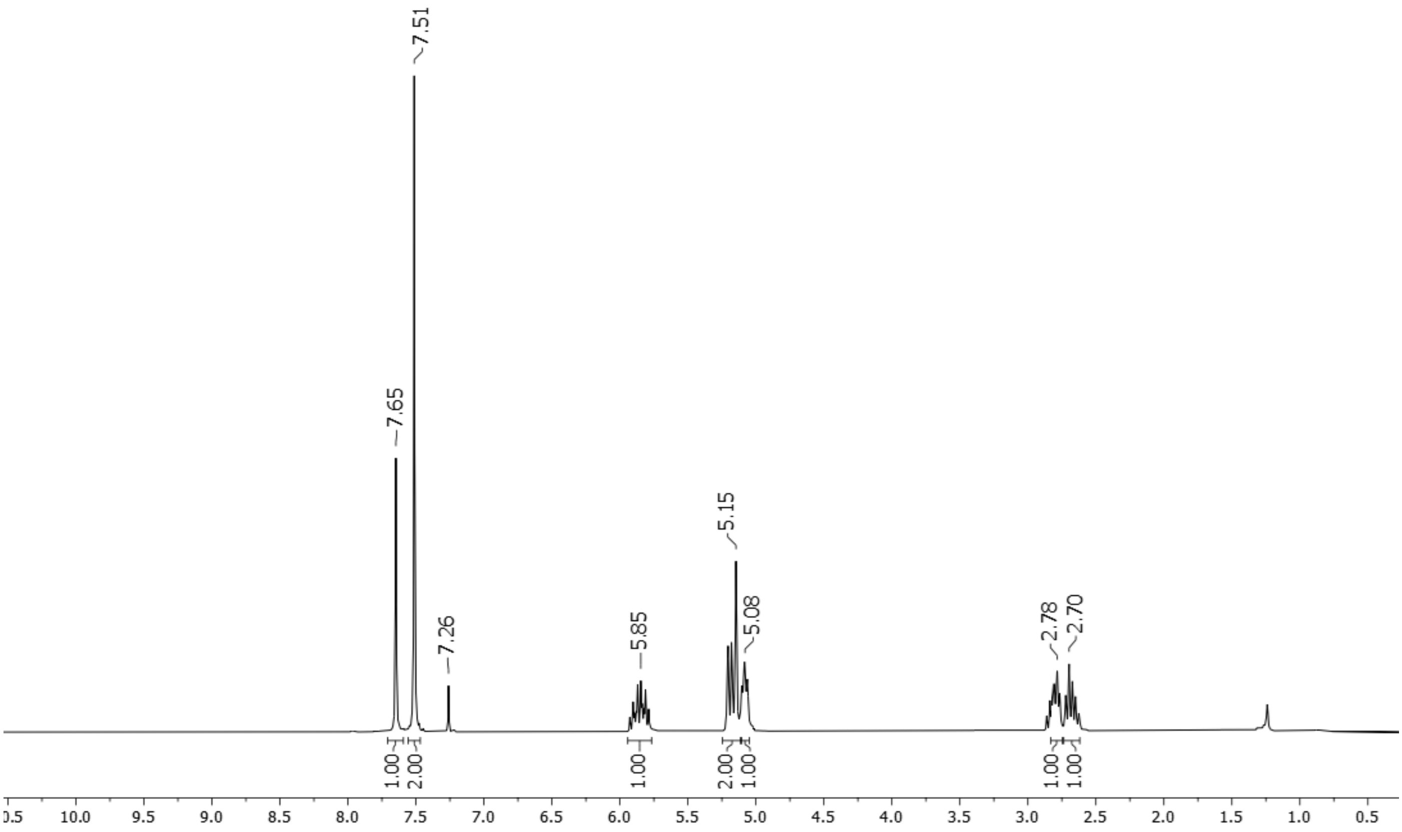

Figure S62. NMR spectra of **2b** after isolation (Entry 19).

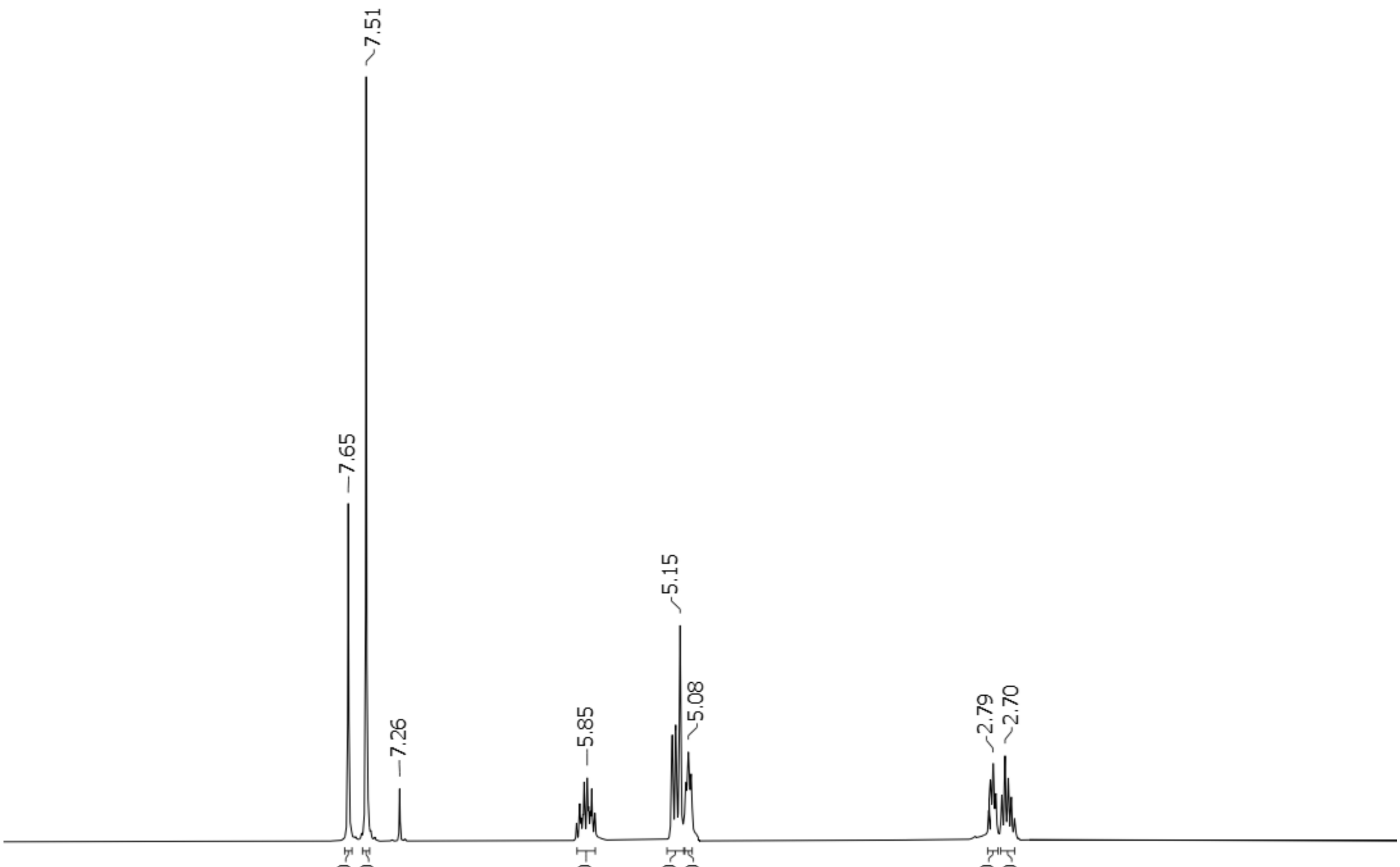

Figure S63. NMR spectra of **2b** after isolation (Entry 20).

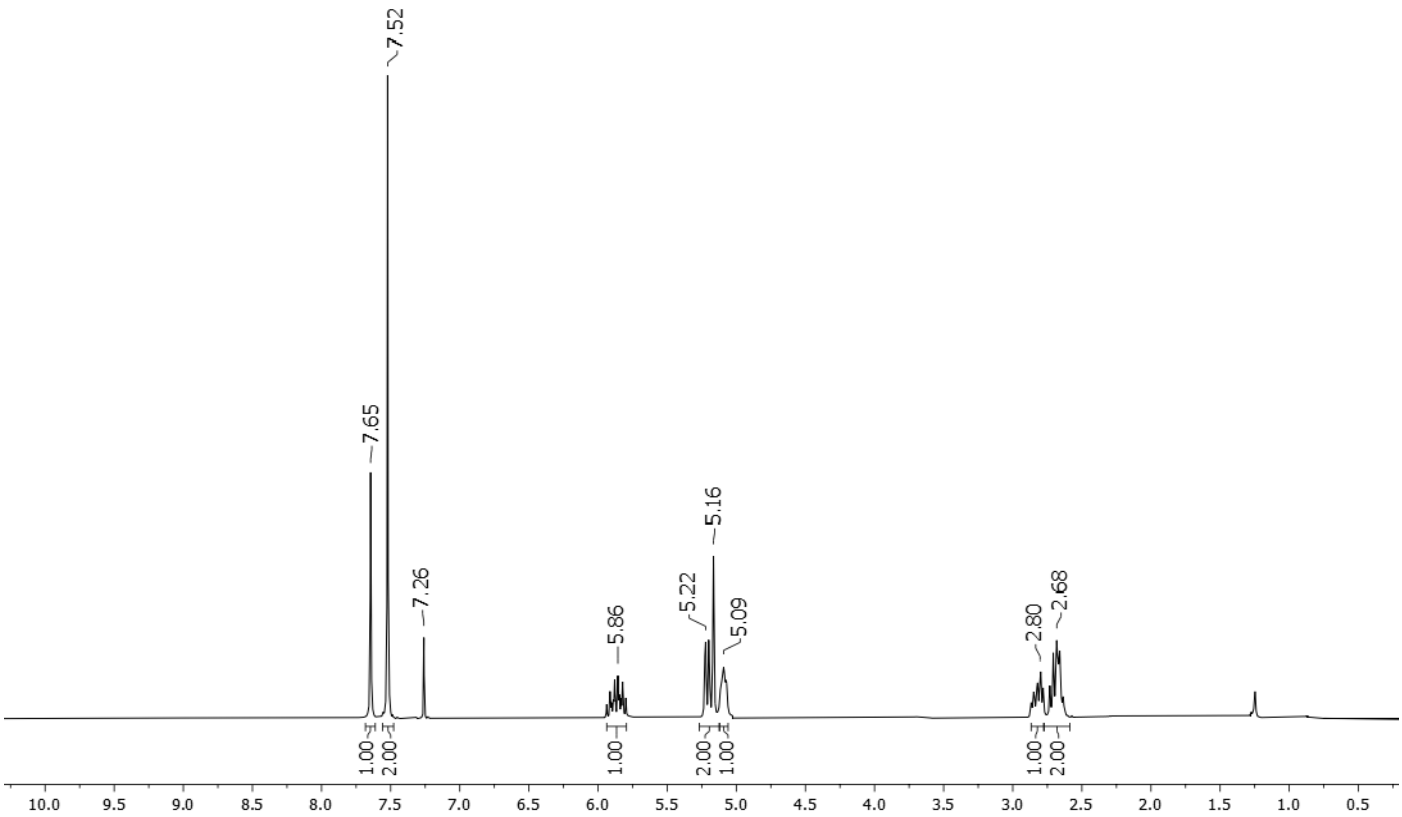

Figure S64. NMR spectra of **2b** after isolation (Entry 21).

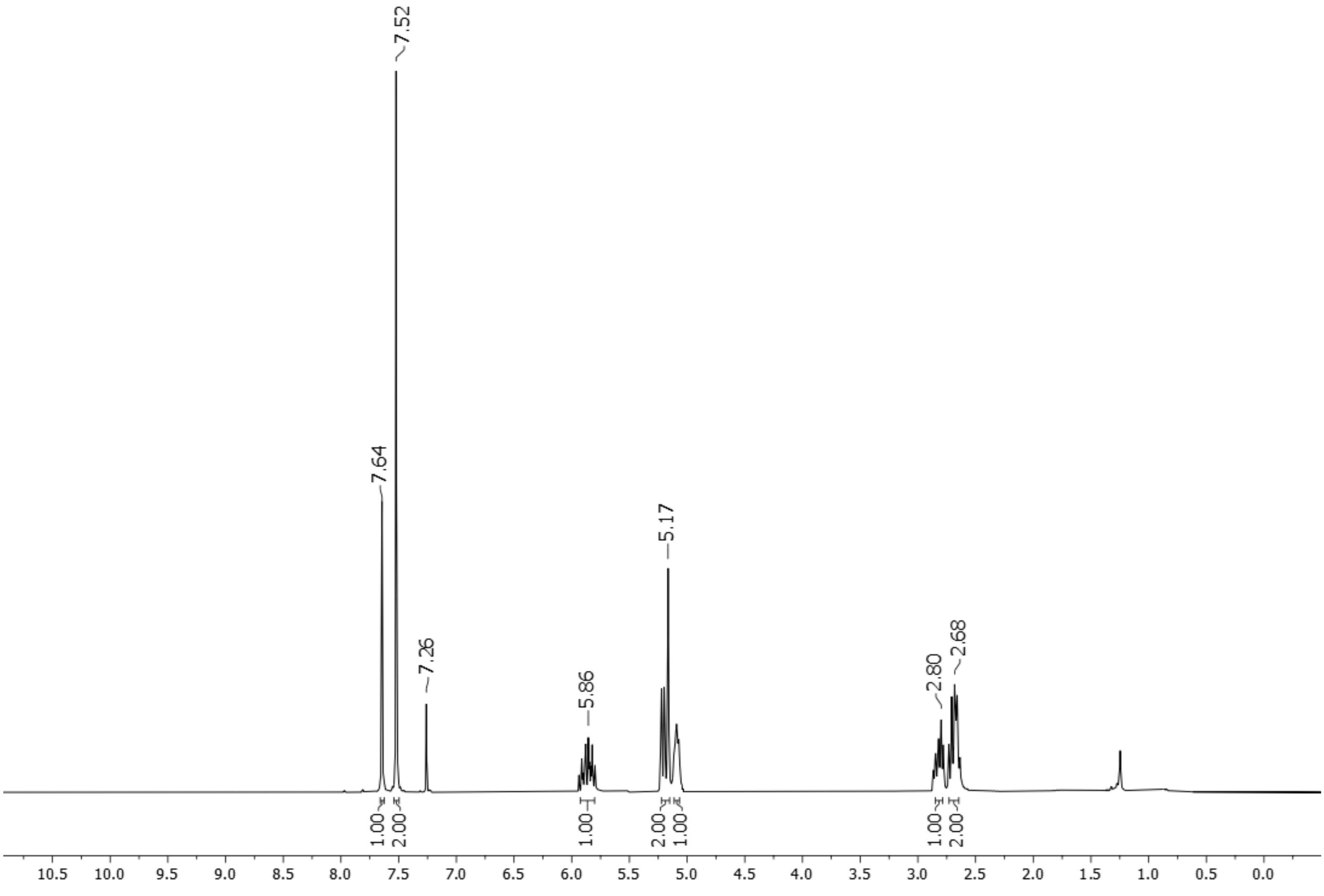

Figure S65. NMR spectra of **2b** after isolation (Entry 22).

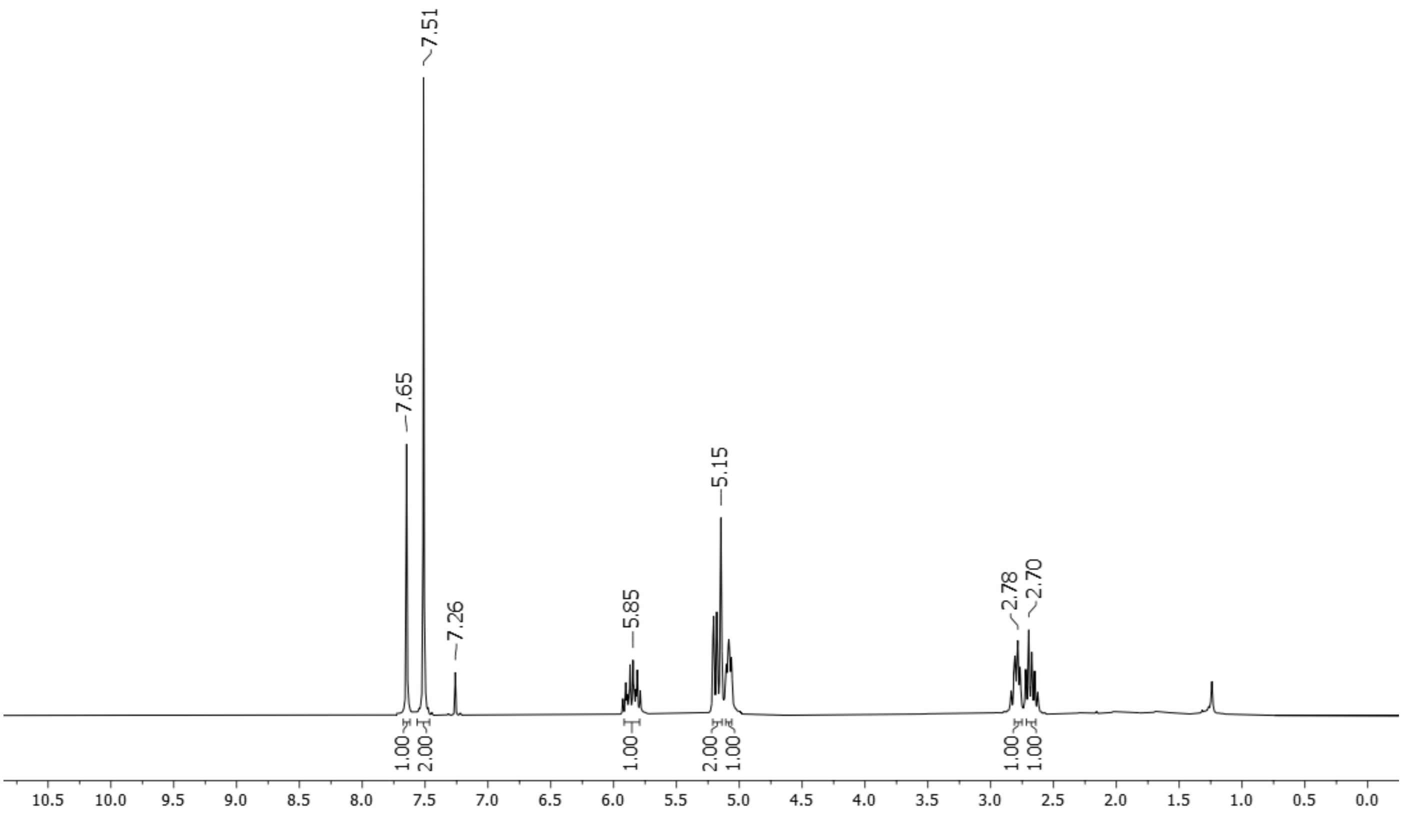

Figure S66. NMR spectra of **2b** after isolation (Entry 23).

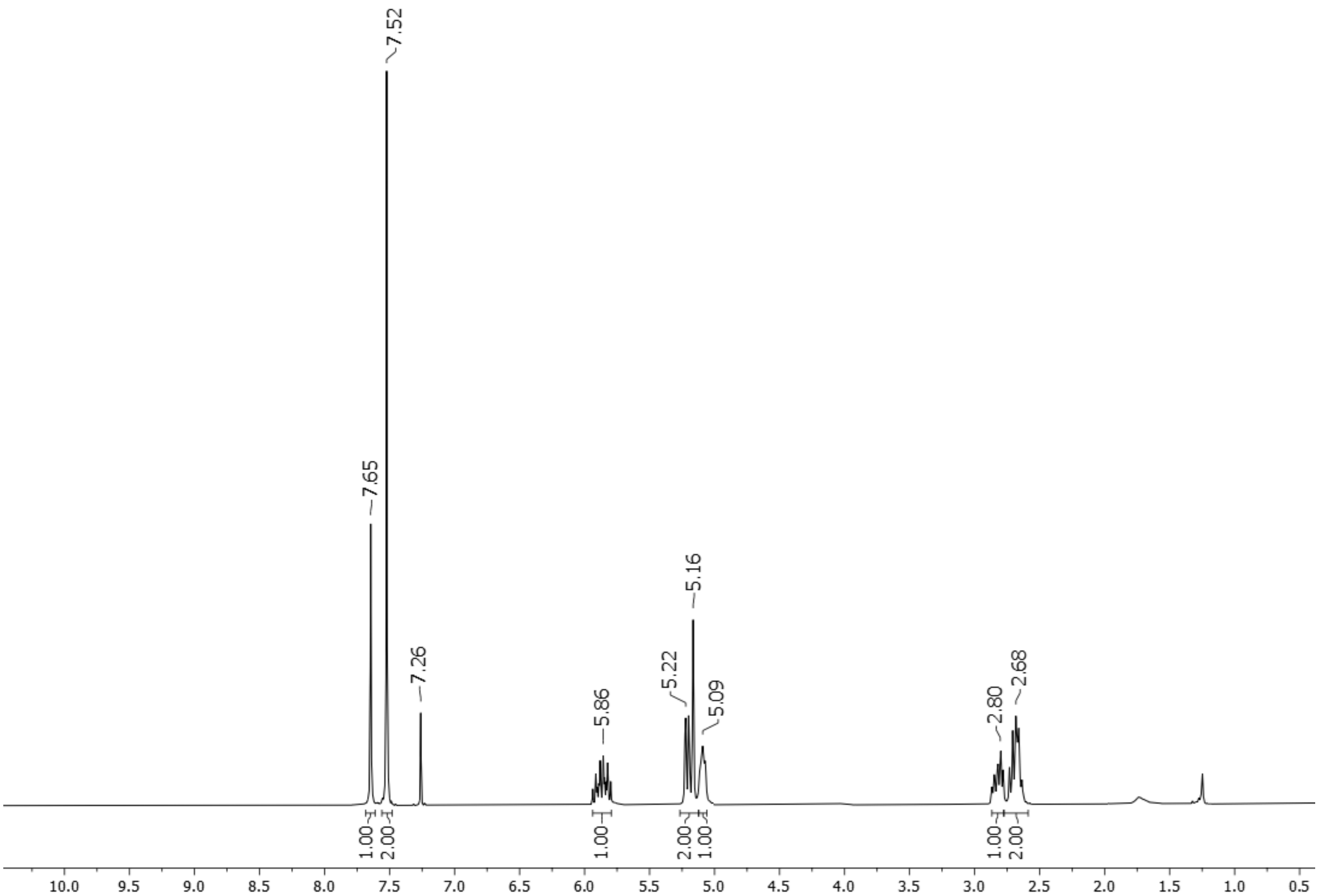

Figure S67. NMR spectra of **2b** after isolation (Entry 24).

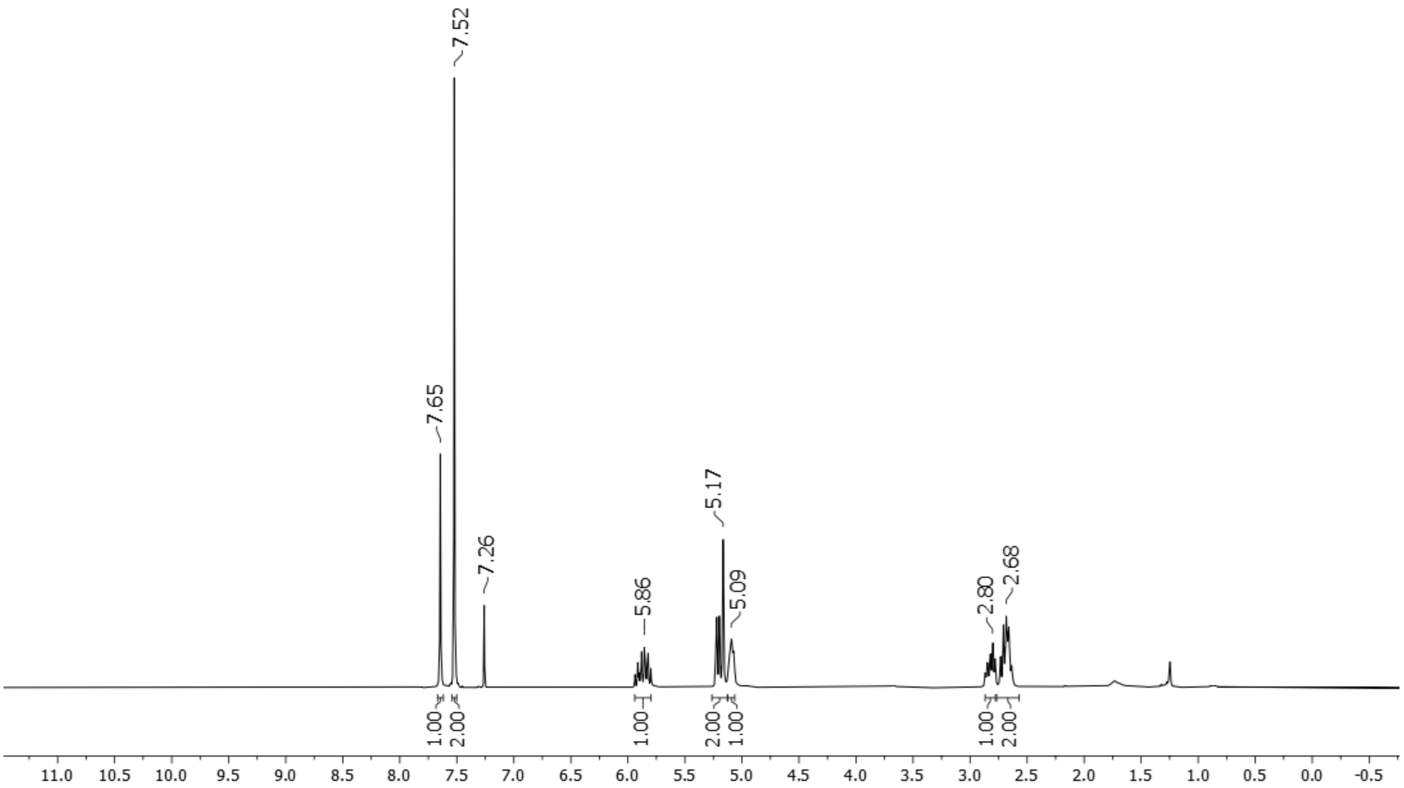

Figure S68. NMR spectra of **2b** after isolation (Entry 25).

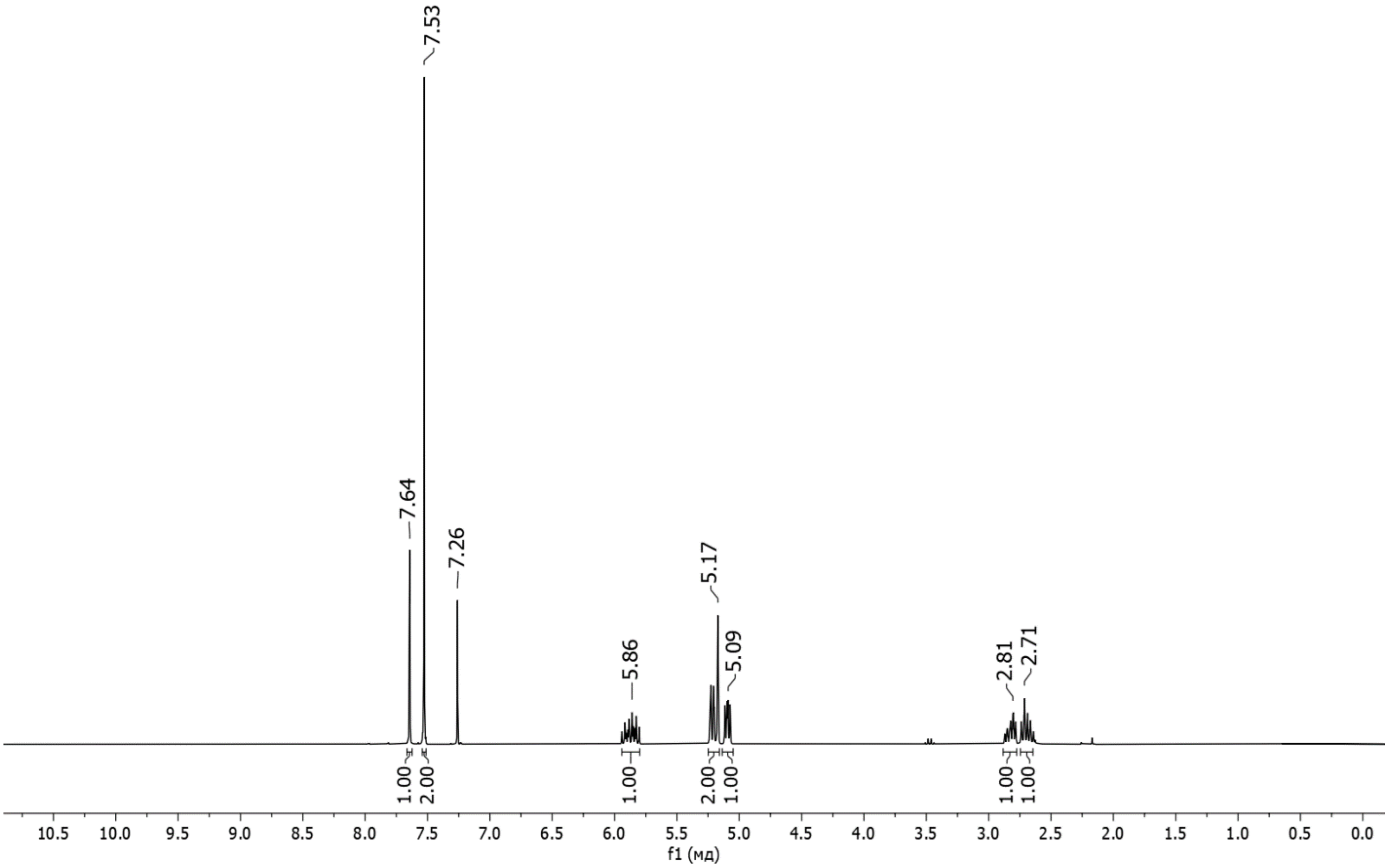

Figure S69. NMR spectra of **2b** after isolation (Entry 26).

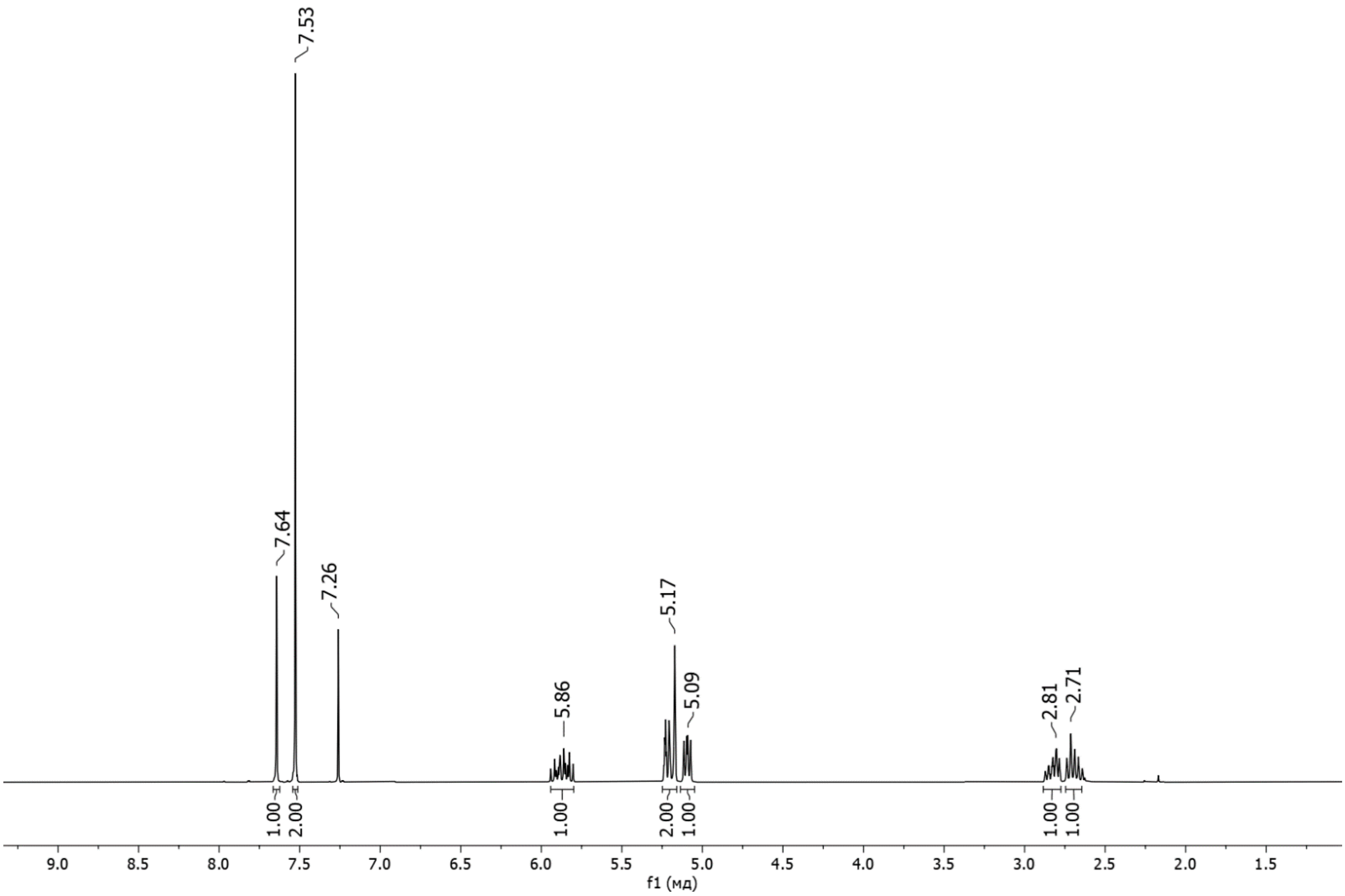

Figure S70. NMR spectra of **2b** after isolation (Entry 27).

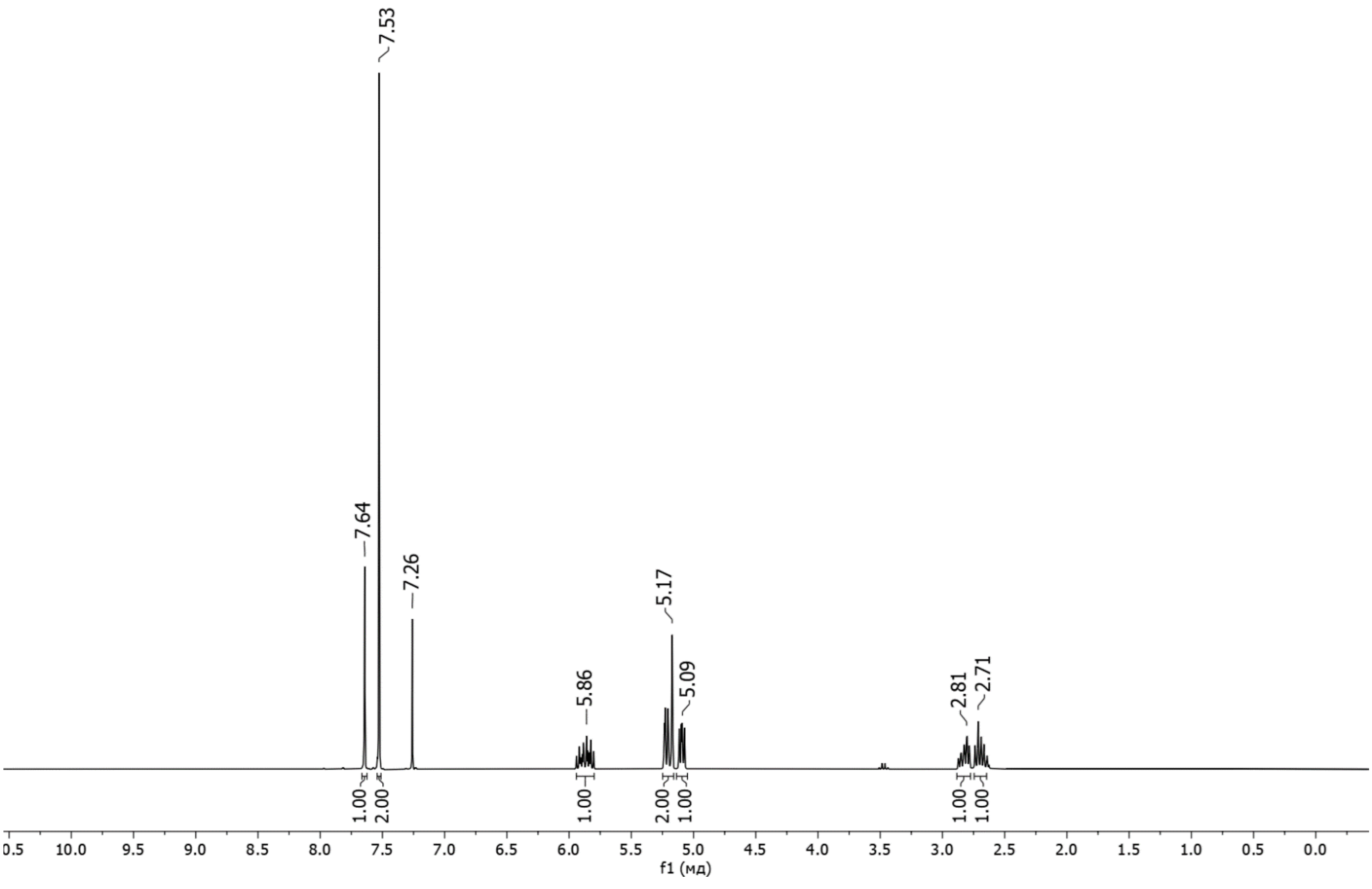

Figure S71. NMR spectra of **2b** after isolation (Entry 28).

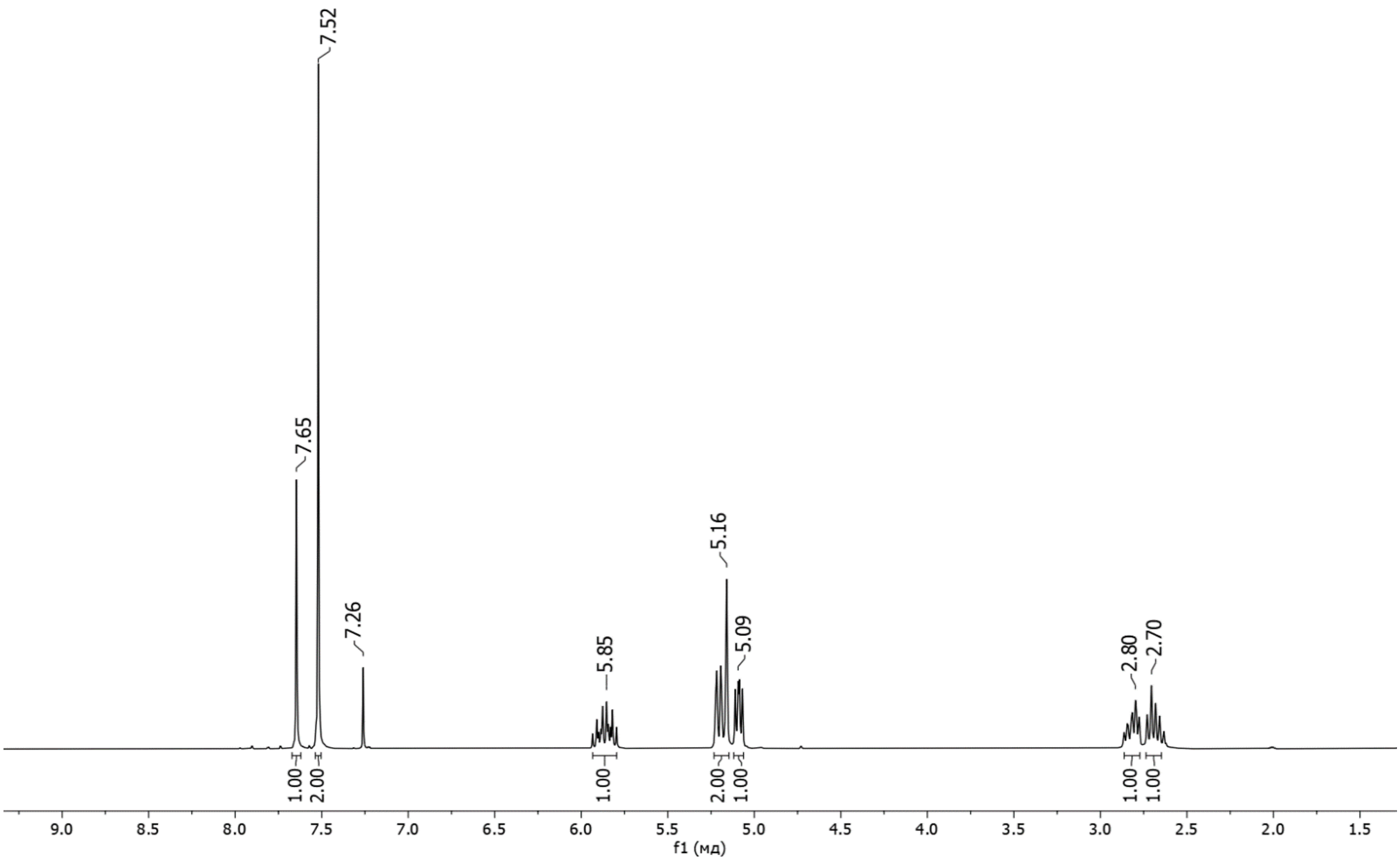

Figure S72. NMR spectra of **2b** after isolation (Entry 29).

5. Cartesian coordinates of optimized structures

B(all)$_3$

Electronic Energy = -376.684387
Sum of electronic and zero-point Energies = -376.466739
Sum of electronic and thermal Enthalpies = -376.453175
Sum of electronic and thermal Free Energies = -376.507021

Standard orientation:

Center Number	Atomic Number	Atomic Type	Coordinates (Angstroms) X	Y	Z
1	5	0	-0.267760	-0.041491	0.071484
2	6	0	0.431726	-0.949573	-1.019978
3	6	0	-1.610173	-0.534127	0.757468
4	1	0	-1.592890	-1.617686	0.926370
5	1	0	-1.799972	-0.034597	1.713781
6	6	0	1.533713	-1.598147	-0.218372
7	6	0	1.448446	-2.789164	0.373961
8	1	0	2.431204	-0.996941	-0.077296
9	1	0	2.264720	-3.184394	0.971304
10	1	0	0.567393	-3.418465	0.267149
11	6	0	-2.690675	-0.187301	-0.233818
12	6	0	-3.480991	0.883228	-0.163661
13	1	0	-2.779804	-0.848203	-1.095853
14	1	0	-4.217412	1.096457	-0.932783
15	1	0	-3.429154	1.575554	0.674020
16	1	0	-0.237383	-1.710955	-1.433970
17	1	0	0.866182	-0.369221	-1.841057
18	6	0	0.358086	1.335531	0.545080
19	1	0	0.689571	1.179715	1.583789
20	6	0	1.471203	1.901038	-0.281824
21	6	0	2.749430	1.974033	0.084285
22	1	0	1.192899	2.253898	-1.275496
23	1	0	3.510753	2.374892	-0.578514
24	1	0	3.079362	1.638729	1.065756
25	1	0	-0.471262	2.055564	0.616537

1a

Electronic Energy = -1046.010324
Sum of electronic and zero-point Energies = -1045.868782
Sum of electronic and thermal Enthalpies = -1045.856749
Sum of electronic and thermal Free Energies = -1045.907558

Standard orientation:

Center Number	Atomic Number	Atomic Type	Coordinates (Angstroms) X	Y	Z
1	6	0	-4.647381	-0.343619	-0.119366
2	8	0	-4.949519	-1.473998	-0.442533
3	1	0	-5.406586	0.432118	0.092666
4	6	0	-3.262127	0.111211	0.023340
5	6	0	-2.085794	-0.580987	-0.169722
6	1	0	-1.880285	-1.592880	-0.480409
7	7	0	-1.126331	0.323937	0.094698
8	7	0	-2.954031	1.388439	0.388771
9	7	0	-1.671802	1.519325	0.434646
10	6	0	0.287582	0.166006	0.056198
11	6	0	0.853892	-1.049898	0.424927
12	6	0	1.077954	1.236892	-0.349844
13	6	0	2.233479	-1.204684	0.372589
14	1	0	0.230432	-1.868820	0.766700
15	6	0	2.458145	1.088478	-0.387259
16	1	0	0.617703	2.175468	-0.634815
17	6	0	3.020996	-0.132453	-0.030941
18	1	0	2.687952	-2.146710	0.656302
19	1	0	3.086576	1.913959	-0.700827
20	17	0	4.759119	-0.322868	-0.088569

1b

Electronic Energy = -1965.155089
Sum of electronic and zero-point Energies = -1965.033340
Sum of electronic and thermal Enthalpies = -1965.018652
Sum of electronic and thermal Free Energies = -1965.076927

Standard orientation:

Center Number	Atomic Number	Atomic Type	Coordinates (Angstroms) X	Y	Z
1	6	0	-4.678313	-0.000000	0.413493
2	8	0	-4.980837	0.000698	1.588497
3	1	0	-5.436020	-0.000682	-0.392106
4	6	0	-3.291031	-0.000027	-0.060429
5	6	0	-2.117807	0.000677	0.660187
6	1	0	-1.903505	0.001510	1.717174
7	7	0	-1.159292	0.000192	-0.284486
8	7	0	-2.980145	-0.000912	-1.390280
9	7	0	-1.701716	-0.000839	-1.533267
10	6	0	0.248616	0.000141	-0.120819
11	6	0	0.954449	-1.203132	-0.048704
12	6	0	0.954716	1.203257	-0.048827
13	6	0	2.334646	-1.215983	0.096766

14	6	0	2.334909	1.215848	0.096661
15	6	0	3.003010	-0.000138	0.167770
16	1	0	2.873022	-2.153911	0.149813
17	1	0	2.873450	2.153682	0.149643
18	17	0	0.099117	-2.710424	-0.140487
19	17	0	4.734533	-0.000312	0.351388
20	17	0	0.099800	2.710787	-0.141068

1c

Electronic Energy = -3157.560193
Sum of electronic and zero-point Energies = -3157.419091
Sum of electronic and thermal Enthalpies = -3157.406813
Sum of electronic and thermal Free Energies = -3157.459038

Standard orientation:

Center Number	Atomic Number	Atomic Type	Coordinates (Angstroms) X	Y	Z
1	6	0	-5.372705	-0.400503	-0.129063
2	8	0	-5.651217	-1.539692	-0.442197
3	1	0	-6.147811	0.362830	0.069995
4	6	0	-3.997260	0.082193	0.016842
5	6	0	-2.806846	-0.589356	-0.161345
6	1	0	-2.580986	-1.600956	-0.458618
7	7	0	-1.866469	0.337459	0.096713
8	7	0	-3.715939	1.369515	0.368604
9	7	0	-2.436952	1.526069	0.419229
10	6	0	-0.449633	0.208035	0.066100
11	6	0	0.139033	-0.997806	0.432547
12	6	0	0.321574	1.296376	-0.330298
13	6	0	1.521952	-1.123037	0.388854
14	1	0	-0.469127	-1.831486	0.765883
15	6	0	1.704724	1.175138	-0.359768
16	1	0	-0.155622	2.226820	-0.614058
17	6	0	2.291776	-0.034646	-0.004906
18	1	0	1.989908	-2.058326	0.672456
19	1	0	2.314368	2.017099	-0.666394
20	35	0	4.179395	-0.203660	-0.055351

1d

Electronic Energy = -586.430977
Sum of electronic and zero-point Energies = -586.279726
Sum of electronic and thermal Enthalpies = -586.268903
Sum of electronic and thermal Free Energies = -586.316397

Standard orientation:

Center Number	Atomic Number	Atomic Type	Coordinates (Angstroms) X	Y	Z
1	6	0	-3.811121	0.197197	0.091175
2	8	0	-4.178115	1.302797	0.433313
3	1	0	-4.525448	-0.612765	-0.148242
4	6	0	-2.402913	-0.181950	-0.040650
5	6	0	-1.265174	0.564684	0.182206
6	1	0	-1.113309	1.578203	0.517364
7	7	0	-0.258006	-0.284064	-0.087251
8	7	0	-2.025409	-1.433809	-0.427421
9	7	0	-0.736786	-1.497640	-0.458406
10	6	0	1.146729	-0.049394	-0.027589
11	6	0	1.646699	1.197589	-0.390325
12	6	0	1.987090	-1.075595	0.393946
13	6	0	3.017020	1.422187	-0.312985
14	1	0	0.977499	1.975570	-0.742858
15	6	0	3.356175	-0.840623	0.450750
16	1	0	1.569799	-2.036285	0.672695
17	6	0	3.872398	0.405887	0.104301
18	1	0	3.415361	2.391758	-0.592671
19	1	0	4.019665	-1.634637	0.776871
20	1	0	4.941352	0.584489	0.156913

1e

Electronic Energy = -625.739647
Sum of electronic and zero-point Energies = -625.559416
Sum of electronic and thermal Enthalpies = -625.548093
Sum of electronic and thermal Free Energies = -625.597368

Standard orientation:

Center Number	Atomic Number	Atomic Type	Coordinates (Angstroms) X	Y	Z
1	6	0	3.730261	0.970178	0.336575
2	8	0	3.846871	2.042952	-0.220273
3	1	0	4.488312	0.591659	1.048211
4	6	0	2.594351	0.070000	0.131742
5	6	0	1.480197	0.220690	-0.669989
6	1	0	1.151646	1.011699	-1.325521
7	7	0	0.782480	-0.906449	-0.473042
8	7	0	2.503778	-1.131140	0.769058
9	7	0	1.410023	-1.717877	0.404757
10	6	0	-0.517022	-1.285502	-1.018939

11	1	0	-0.626759	-2.350126	-0.801110
12	1	0	-0.481069	-1.158415	-2.102790
13	6	0	-1.641709	-0.478381	-0.411007
14	6	0	-1.801935	-0.434962	0.976430
15	6	0	-2.528121	0.222193	-1.226377
16	6	0	-2.838286	0.301537	1.537697
17	1	0	-1.108266	-0.976300	1.614794
18	6	0	-3.571452	0.955147	-0.663781
19	1	0	-2.402639	0.196746	-2.305351
20	6	0	-3.726475	0.997043	0.717839
21	1	0	-2.955557	0.332804	2.616378
22	1	0	-4.257391	1.497892	-1.306655
23	1	0	-4.536068	1.571030	1.157674

2a

Electronic Energy = -1163.903274
Sum of electronic and zero-point Energies = -1163.676020
Sum of electronic and thermal Enthalpies = -1163.659662
Sum of electronic and thermal Free Energies = -1163.721271

Standard orientation:

Center Number	Atomic Number	Atomic Type	Coordinates (Angstroms) X	Y	Z
1	6	0	2.024096	-0.009266	-0.357377
2	6	0	0.851350	0.653215	-0.100073
3	1	0	0.639777	1.663627	0.206884
4	6	0	-1.516879	-0.160800	-0.147452
5	6	0	-2.260575	-1.259199	0.275719
6	6	0	-2.134450	1.051938	-0.438905
7	6	0	-3.638239	-1.144004	0.405879
8	1	0	-1.762875	-2.194784	0.501048
9	6	0	-3.510905	1.174167	-0.292875
10	1	0	-1.553207	1.896318	-0.792772
11	6	0	-4.249075	0.073442	0.125627
12	1	0	-4.227123	-1.993127	0.732866
13	1	0	-4.001648	2.114289	-0.516521
14	7	0	0.438546	-1.463400	-0.636326
15	7	0	1.725819	-1.297242	-0.678105
16	7	0	-0.108957	-0.286854	-0.283737
17	6	0	3.430735	0.505017	-0.306942
18	6	0	4.315413	-0.372590	0.596599
19	1	0	3.870394	-0.375716	1.598988
20	1	0	4.286759	-1.397863	0.213687
21	6	0	5.731097	0.124027	0.661324
22	6	0	6.776376	-0.499968	0.124454
23	1	0	5.889262	1.068003	1.183274

24	1	0	7.780493	-0.090635	0.191714
25	1	0	6.665673	-1.449127	-0.395881
26	8	0	3.352892	1.842883	0.155797
27	1	0	4.184079	2.276244	-0.060740
28	1	0	3.848935	0.476480	-1.324249
29	17	0	-5.985010	0.222343	0.301566

2b

Electronic Energy = -2083.052817
Sum of electronic and zero-point Energies = -2082.844951
Sum of electronic and thermal Enthalpies = -2082.826272
Sum of electronic and thermal Free Energies = -2082.895019

Standard orientation:

Center Number	Atomic Number	Atomic Type	Coordinates (Angstroms) X	Y	Z
1	6	0	-2.392462	-0.828543	-0.112696
2	6	0	-1.255881	-0.577121	0.610945
3	1	0	-1.060535	-0.483309	1.666546
4	6	0	1.084499	-0.157037	-0.149182
5	6	0	2.020536	-1.182737	0.003583
6	6	0	1.531381	1.166637	-0.131425
7	6	0	3.370431	-0.905856	0.172548
8	6	0	2.875316	1.470823	0.035078
9	6	0	3.774833	0.422948	0.185034
10	1	0	4.086856	-1.709654	0.289101
11	1	0	3.209751	2.500687	0.046128
12	7	0	-0.792821	-0.611828	-1.569709
13	7	0	-2.061125	-0.839258	-1.434886
14	7	0	-0.287589	-0.450537	-0.330013
15	6	0	-3.797384	-1.043721	0.387817
16	17	0	5.465077	0.787932	0.395407
17	17	0	0.389194	2.461554	-0.318894
18	17	0	1.494108	-2.836903	-0.017548
19	8	0	-3.874402	-0.745822	1.765892
20	1	0	-3.922836	0.217791	1.836540
21	6	0	-4.824647	-0.235645	-0.429276
22	1	0	-4.756500	-0.545495	-1.476736
23	1	0	-5.821125	-0.494244	-0.057827
24	6	0	-4.581602	1.241095	-0.299040
25	6	0	-5.262491	2.040566	0.521542
26	1	0	-3.756858	1.649775	-0.881487
27	1	0	-5.028350	3.097629	0.610669
28	1	0	-6.095298	1.668546	1.115284
29	1	0	-4.046755	-2.106161	0.294918

2c

Electronic Energy = -3275.453155
Sum of electronic and zero-point Energies = -3275.226369
Sum of electronic and thermal Enthalpies = -3275.209752
Sum of electronic and thermal Free Energies = -3275.272803

Standard orientation:

Center Number	Atomic Number	Atomic Type	Coordinates (Angstroms) X	Y	Z
1	6	0	2.791299	0.021222	0.359065
2	6	0	1.608446	-0.633243	0.129537
3	1	0	1.383111	-1.646664	-0.156861
4	6	0	-0.750263	0.205357	0.194952
5	6	0	-1.491167	1.306270	-0.226614
6	6	0	-1.373477	-0.999803	0.505194
7	6	0	-2.871379	1.199872	-0.337451
8	1	0	-0.989523	2.236148	-0.466487
9	6	0	-2.752821	-1.110981	0.379260
10	1	0	-0.794789	-1.846286	0.858242
11	6	0	-3.489468	-0.009475	-0.039260
12	1	0	-3.454767	2.052889	-0.663986
13	1	0	-3.244446	-2.046546	0.618675
14	7	0	1.224882	1.496777	0.633449
15	7	0	2.510790	1.317791	0.660889
16	7	0	0.660424	0.319909	0.309504
17	6	0	4.191124	-0.510301	0.299485
18	6	0	5.077354	0.345975	-0.622676
19	1	0	4.620424	0.346995	-1.619667
20	1	0	5.068747	1.374913	-0.248708
21	35	0	-5.373405	-0.159449	-0.205582
22	6	0	6.484392	-0.173149	-0.699765
23	6	0	7.545139	0.437266	-0.177832
24	1	0	6.621754	-1.122659	-1.217548
25	1	0	8.541859	0.011612	-0.253353
26	1	0	7.455071	1.391063	0.337980
27	8	0	4.091713	-1.852087	-0.147513
28	1	0	4.919782	-2.293381	0.064951
29	1	0	4.620500	-0.476075	1.311965

2d

Electronic Energy = -704.324149
Sum of electronic and zero-point Energies = -704.087190
Sum of electronic and thermal Enthalpies = -704.072097
Sum of electronic and thermal Free Energies = -704.130356

Standard orientation:

Center Number	Atomic Number	Atomic Type	Coordinates (Angstroms) X	Y	Z
1	6	0	1.181752	0.017449	0.515313
2	6	0	0.063430	-0.620581	0.039158
3	1	0	-0.081033	-1.562132	-0.464522
4	6	0	-2.338388	0.086968	0.031894
5	6	0	-3.097402	1.211247	-0.282345
6	6	0	-2.912952	-1.180146	0.082717
7	6	0	-4.453775	1.057153	-0.545689
8	1	0	-2.625397	2.186143	-0.319062
9	6	0	-4.267740	-1.321042	-0.199326
10	1	0	-2.314196	-2.043359	0.353943
11	6	0	-5.040528	-0.205677	-0.510830
12	1	0	-5.050976	1.929660	-0.789578
13	1	0	-4.720255	-2.306508	-0.162492
14	1	0	-6.098289	-0.319903	-0.724307
15	7	0	-0.487792	1.347017	0.908301
16	7	0	0.799076	1.214904	1.035363
17	7	0	-0.950144	0.239325	0.303929
18	6	0	2.616610	-0.428408	0.471435
19	6	0	3.371032	0.235524	-0.686537
20	1	0	2.898349	-0.097803	-1.619768
21	1	0	3.232312	1.319104	-0.613346
22	6	0	4.832228	-0.108619	-0.694191
23	6	0	5.815083	0.782591	-0.598516
24	1	0	5.070696	-1.167585	-0.774798
25	1	0	6.858873	0.481047	-0.606334
26	1	0	5.612026	1.848258	-0.512559
27	8	0	2.697465	-1.831268	0.282104
28	1	0	2.394308	-2.255158	1.091951
29	1	0	3.099788	-0.129104	1.412386

2e

Electronic Energy = -743.634475
Sum of electronic and zero-point Energies = -743.368264
Sum of electronic and thermal Enthalpies = -743.351923
Sum of electronic and thermal Free Energies = -743.414622

Standard orientation:

Center Number	Atomic Number	Atomic Type	Coordinates (Angstroms) X	Y	Z
1	6	0	1.487393	-1.030964	-0.047746

2	6	0	0.334464	-0.866620	-0.776385
3	1	0	0.116168	-0.349458	-1.696072
4	7	0	-0.070761	-2.083848	1.030259
5	7	0	1.196838	-1.779048	1.048089
6	7	0	-0.599960	-1.541697	-0.072110
7	6	0	2.860271	-0.492951	-0.306482
8	6	0	3.234746	0.590740	0.724960
9	1	0	2.502066	1.401889	0.649789
10	1	0	3.157570	0.152791	1.725742
11	6	0	-2.024248	-1.668382	-0.343565
12	1	0	-2.151171	-2.026866	-1.367570
13	1	0	-2.386510	-2.443325	0.336100
14	6	0	-2.763950	-0.365974	-0.131313
15	6	0	-2.603503	0.346331	1.059955
16	6	0	-3.619747	0.128301	-1.113722
17	6	0	-3.293023	1.535903	1.263375
18	1	0	-1.931814	-0.033621	1.825488
19	6	0	-4.315918	1.317983	-0.907825
20	1	0	-3.743279	-0.417817	-2.045258
21	6	0	-4.152667	2.023804	0.279769
22	1	0	-3.161121	2.083386	2.191485
23	1	0	-4.980282	1.694666	-1.679409
24	1	0	-4.690785	2.952968	0.439844
25	6	0	4.622056	1.105464	0.474665
26	6	0	4.896110	2.271997	-0.108451
27	1	0	5.440939	0.439262	0.746166
28	1	0	5.917689	2.586051	-0.302773
29	1	0	4.104992	2.963426	-0.391789
30	8	0	2.864485	0.025092	-1.620747
31	1	0	3.653893	0.575102	-1.700538
32	1	0	3.581038	-1.320836	-0.212304

3a

Electronic Energy = -1422.699998
Sum of electronic and zero-point Energies = -1422.337587
Sum of electronic and thermal Enthalpies = -1422.312388
Sum of electronic and thermal Free Energies = -1422.393682

Standard orientation:

Center Number	Atomic Number	Atomic Type	Coordinates (Angstroms) X	Y	Z
1	8	0	2.770899	-0.638835	1.179843
2	6	0	1.594866	-0.898370	1.423345
3	1	0	1.409960	-1.713603	2.136215
4	6	0	0.398615	-0.273712	0.910245
5	6	0	-0.882810	-0.758392	1.100352

6	7	0	0.318768	0.848604	0.134231
7	1	0	-1.266261	-1.599473	1.656759
8	7	0	-0.908568	1.068298	-0.161859
9	7	0	-1.661455	0.087914	0.414196
10	6	0	-3.074290	0.072539	0.239672
11	6	0	-3.764115	1.280073	0.213803
12	6	0	-3.734691	-1.142538	0.093594
13	6	0	-5.142013	1.270148	0.044657
14	1	0	-3.227831	2.215036	0.324091
15	6	0	-5.114994	-1.153469	-0.060583
16	1	0	-3.179942	-2.074590	0.080108
17	6	0	-5.804834	0.053759	-0.084143
18	1	0	-5.695466	2.201368	0.022655
19	1	0	-5.644262	-2.091755	-0.176151
20	17	0	-7.539874	0.042350	-0.284701
21	5	0	3.648676	0.286917	0.022399
22	6	0	2.971487	-0.036214	-1.427833
23	6	0	3.494407	1.826908	0.524189
24	1	0	2.431075	2.079855	0.614182
25	1	0	3.956771	1.954848	1.512299
26	6	0	2.473863	-1.431607	-1.640056
27	6	0	1.215861	-1.774015	-1.923994
28	1	0	3.204136	-2.230655	-1.512838
29	1	0	0.922374	-2.813067	-2.049830
30	1	0	0.439657	-1.022587	-2.060698
31	6	0	4.132112	2.762936	-0.454280
32	6	0	5.284260	3.411633	-0.283222
33	1	0	3.609374	2.882123	-1.404793
34	1	0	5.702120	4.049491	-1.057287
35	1	0	5.850327	3.327290	0.642532
36	1	0	2.166603	0.671872	-1.654886
37	1	0	3.775702	0.163504	-2.153957
38	6	0	5.146862	-0.286565	0.283330
39	1	0	5.376756	-0.204988	1.354312
40	6	0	5.437243	-1.672497	-0.193175
41	6	0	5.556021	-2.759376	0.570146
42	1	0	5.531590	-1.794724	-1.274025
43	1	0	5.742651	-3.741474	0.143979
44	1	0	5.475250	-2.701239	1.654022
45	1	0	5.818391	0.417745	-0.229750

3b

Electronic Energy = -2341.855371
Sum of electronic and zero-point Energies = -2341.512599
Sum of electronic and thermal Enthalpies = -2341.484906
Sum of electronic and thermal Free Energies = -2341.573832

Standard orientation:

Center Number	Atomic Number	Atomic Type	Coordinates (Angstroms) X	Y	Z
1	6	0	2.029000	-0.628764	1.008527
2	8	0	2.625617	-0.113220	0.056192
3	1	0	2.571090	-1.110524	1.827818
4	6	0	0.594752	-0.609965	1.082727
5	6	0	-0.334000	-0.098947	0.196931
6	1	0	-0.244829	0.404663	-0.753137
7	7	0	-1.509261	-0.364405	0.782047
8	7	0	-0.072184	-1.153919	2.147382
9	7	0	-1.332477	-1.009961	1.975712
10	6	0	-2.818006	-0.071015	0.319311
11	6	0	-3.523431	-1.006465	-0.440774
12	6	0	-3.423564	1.147592	0.633334
13	6	0	-4.809068	-0.737193	-0.887867
14	6	0	-4.708000	1.438695	0.196092
15	6	0	-5.380299	0.486489	-0.560445
16	1	0	-5.350579	-1.466841	-1.476821
17	1	0	-5.170243	2.386092	0.444162
18	5	0	4.288810	-0.103374	-0.076503
19	6	0	4.680506	1.250251	0.750991
20	1	0	4.286876	1.167762	1.776746
21	1	0	4.208276	2.127603	0.290826
22	6	0	4.551987	0.007876	-1.679974
23	1	0	5.634182	0.152414	-1.803570
24	1	0	4.310092	-0.944304	-2.171562
25	6	0	4.862959	-1.458207	0.612416
26	1	0	4.866693	-1.377948	1.713034
27	1	0	5.936586	-1.484121	0.365797
28	6	0	6.161329	1.452704	0.815030
29	6	0	6.850466	2.391404	0.165122
30	1	0	6.716153	0.735998	1.423623
31	1	0	7.933455	2.454419	0.231454
32	1	0	6.350035	3.131628	-0.455996
33	6	0	3.822163	1.119380	-2.364671
34	6	0	4.363500	2.238733	-2.847064
35	1	0	2.739141	1.002606	-2.437343
36	1	0	3.758245	3.017260	-3.304642
37	1	0	5.436948	2.412350	-2.802653
38	6	0	4.298748	-2.814357	0.272821
39	6	0	3.313320	-3.114717	-0.575481
40	1	0	4.757055	-3.649357	0.807646
41	1	0	3.003124	-4.145360	-0.726469
42	1	0	2.792712	-2.363870	-1.162653
43	17	0	-2.786061	-2.525880	-0.837256
44	17	0	-6.990612	0.837028	-1.118552
45	17	0	-2.563369	2.318803	1.581019

3c

Electronic Energy = -3534.263863
Sum of electronic and zero-point Energies = -3533.902272
Sum of electronic and thermal Enthalpies = -3533.876670
Sum of electronic and thermal Free Energies = -3533.961831

Standard orientation:

Center Number	Atomic Number	Atomic Type	Coordinates (Angstroms) X	Y	Z
1	6	0	2.411510	-0.918176	-0.418745
2	8	0	2.847164	0.223295	-0.219092
3	1	0	3.079888	-1.763986	-0.604563
4	6	0	0.997557	-1.161649	-0.412314
5	6	0	-0.044460	-0.277479	-0.195989
6	1	0	-0.065876	0.783515	0.000199
7	7	0	-1.139413	-1.038326	-0.311312
8	7	0	0.474223	-2.401454	-0.649826
9	7	0	-0.805919	-2.332527	-0.588372
10	6	0	-2.508134	-0.663346	-0.184386
11	6	0	-2.872647	0.289807	0.760273
12	6	0	-3.450895	-1.264252	-1.011864
13	6	0	-4.206290	0.663143	0.868323
14	1	0	-2.131361	0.728876	1.419349
15	6	0	-4.785707	-0.900441	-0.892824
16	1	0	-3.144382	-2.004084	-1.741792
17	6	0	-5.149739	0.063208	0.041807
18	1	0	-4.503540	1.405274	1.599663
19	1	0	-5.530312	-1.360329	-1.531858
20	5	0	4.432658	0.627411	-0.075258
21	6	0	5.276383	-0.396018	-1.027825
22	1	0	4.804325	-0.441645	-2.021801
23	1	0	5.274287	-1.415692	-0.617859
24	6	0	4.750242	0.431734	1.522187
25	1	0	5.842747	0.502514	1.612859
26	1	0	4.325198	1.257270	2.106566
27	6	0	4.458322	2.171891	-0.591811
28	1	0	4.279016	2.206443	-1.676061
29	1	0	5.483296	2.538407	-0.433739
30	6	0	6.694365	0.054896	-1.187001
31	6	0	7.760733	-0.505649	-0.614940
32	1	0	6.846557	0.946177	-1.797670
33	1	0	8.759628	-0.098254	-0.745521
34	1	0	7.670934	-1.397723	0.002386
35	6	0	4.273427	-0.866356	2.088663
36	6	0	4.984725	-1.989817	2.205562
37	1	0	3.224507	-0.898543	2.392417
38	1	0	4.547331	-2.906263	2.592729

39	1	0	6.037153	-2.024298	1.930619
40	6	0	3.502977	3.087588	0.104391
41	6	0	2.394018	3.611309	-0.419737
42	1	0	3.732743	3.316568	1.146474
43	1	0	1.734402	4.252418	0.159589
44	1	0	2.112633	3.423914	-1.454846
45	35	0	-6.969724	0.567137	0.195310

3d

Electronic Energy = -963.139293
Sum of electronic and zero-point Energies = -962.766924
Sum of electronic and thermal Enthalpies = -962.743221
Sum of electronic and thermal Free Energies = -962.821226

Standard orientation:

Center Number	Atomic Number	Atomic Type	Coordinates (Angstroms) X	Y	Z
1	6	0	-0.764162	-0.658139	0.618119
2	8	0	-1.404796	0.098425	-0.131761
3	1	0	-1.266878	-1.350084	1.296485
4	6	0	0.670513	-0.597730	0.677732
5	6	0	1.563634	0.175572	-0.040553
6	1	0	1.430404	0.906614	-0.822477
7	7	0	2.761846	-0.195079	0.430396
8	7	0	1.377048	-1.392330	1.535100
9	7	0	2.631234	-1.151519	1.390530
10	6	0	4.055774	0.276648	0.057091
11	6	0	4.229595	1.622886	-0.247713
12	6	0	5.113044	-0.626668	0.009174
13	6	0	5.492219	2.067652	-0.624503
14	1	0	3.398044	2.315804	-0.173889
15	6	0	6.372075	-0.163159	-0.354832
16	1	0	4.946374	-1.670084	0.250814
17	6	0	6.562621	1.178670	-0.676580
18	1	0	5.639156	3.115292	-0.864495
19	1	0	7.204485	-0.857671	-0.394560
20	1	0	7.546908	1.532425	-0.965166
21	5	0	-2.992771	-0.084351	-0.424996
22	6	0	-3.415128	1.352110	-1.063377
23	1	0	-2.713887	1.597141	-1.874885
24	1	0	-3.324030	2.142273	-0.306485
25	6	0	-3.763762	-0.451370	0.966832
26	1	0	-4.828704	-0.530795	0.710481
27	1	0	-3.471937	-1.444571	1.341042
28	6	0	-2.993892	-1.310492	-1.530911
29	1	0	-2.471358	-0.979883	-2.437708

30	1	0	-4.043892	-1.482989	-1.800155
31	6	0	-4.808465	1.341544	-1.608473
32	6	0	-5.864596	1.957314	-1.075271
33	1	0	-4.964948	0.740536	-2.506521
34	1	0	-6.856361	1.873183	-1.511925
35	1	0	-5.769316	2.570834	-0.181438
36	6	0	-3.590706	0.554938	2.061460
37	6	0	-4.524824	1.389982	2.517158
38	1	0	-2.592073	0.624745	2.500063
39	1	0	-4.311399	2.117834	3.295594
40	1	0	-5.539536	1.370900	2.124621
41	6	0	-2.387454	-2.562026	-1.008061
42	6	0	-1.116803	-2.945044	-1.189055
43	1	0	-3.014628	-3.170148	-0.354799
44	1	0	-0.717004	-3.842966	-0.726365
45	1	0	-0.446186	-2.387673	-1.840772

3e

Electronic Energy = -1002.440670
Sum of electronic and zero-point Energies = -1002.039530
Sum of electronic and thermal Enthalpies = -1002.014198
Sum of electronic and thermal Free Energies = -1002.097719

Standard orientation:

Center Number	Atomic Number	Atomic Type	Coordinates (Angstroms) X	Y	Z
1	6	0	1.156126	-1.082999	-0.487083
2	8	0	1.605623	0.068158	-0.584599
3	1	0	1.795765	-1.926028	-0.212659
4	6	0	-0.229976	-1.349196	-0.738699
5	6	0	-1.251057	-0.482024	-1.092304
6	1	0	-1.278816	0.583573	-1.260225
7	7	0	-2.319400	-1.273319	-1.200884
8	7	0	-0.745589	-2.611432	-0.650149
9	7	0	-2.001427	-2.564003	-0.925979
10	5	0	3.134438	0.519937	-0.171218
11	6	0	4.100543	-0.745819	-0.553567
12	1	0	3.857152	-1.097436	-1.568470
13	1	0	3.934416	-1.588872	0.132064
14	6	0	3.079848	0.789127	1.451120
15	1	0	4.126477	0.744271	1.782281
16	1	0	2.721178	1.802966	1.662191
17	6	0	3.415432	1.837209	-1.069503
18	1	0	3.518337	1.557396	-2.131786
19	1	0	4.421774	2.195820	-0.799609
20	6	0	5.548141	-0.371607	-0.506187

21	6	0	6.413215	-0.717603	0.448216
22	1	0	5.901980	0.274140	-1.311297
23	1	0	7.444209	-0.374398	0.437164
24	1	0	6.119009	-1.361381	1.275343
25	6	0	2.262114	-0.201064	2.212339
26	6	0	2.700746	-1.331674	2.771878
27	1	0	1.190273	0.003937	2.264466
28	1	0	2.025755	-2.022124	3.270679
29	1	0	3.756706	-1.595284	2.764256
30	6	0	2.496518	3.030587	-1.010182
31	6	0	1.322088	3.140920	-0.388459
32	1	0	2.849620	3.910495	-1.554031
33	1	0	0.755821	4.068491	-0.424031
34	1	0	0.884256	2.323800	0.176878
35	6	0	-3.700785	-0.910321	-1.515838
36	1	0	-4.218625	-1.857670	-1.678846
37	1	0	-3.696799	-0.352306	-2.454043
38	6	0	-4.337281	-0.109233	-0.404273
39	6	0	-4.421935	-0.642870	0.884392
40	6	0	-4.843520	1.164345	-0.655709
41	6	0	-5.006720	0.093052	1.907935
42	1	0	-4.025193	-1.635108	1.083863
43	6	0	-5.435587	1.899531	0.369494
44	1	0	-4.776608	1.582815	-1.656403
45	6	0	-5.515904	1.365754	1.651462
46	1	0	-5.068541	-0.326536	2.907054
47	1	0	-5.828695	2.890405	0.165251
48	1	0	-5.973742	1.938673	2.451686

TS1a

Electronic Energy = -1422.688979
Sum of electronic and zero-point Energies = -1422.326552
Sum of electronic and thermal Enthalpies = -1422.302512
Sum of electronic and thermal Free Energies = -1422.381817

Standard orientation:

Center Number	Atomic Number	Atomic Type	Coordinates (Angstroms) X	Y	Z
1	8	0	-2.633331	-0.596824	-0.793862
2	6	0	-1.579245	-1.205402	-0.445623
3	1	0	-1.482945	-2.236902	-0.795470
4	6	0	-0.302343	-0.522761	-0.203116
5	6	0	0.960427	-1.030493	-0.408912
6	7	0	-0.179240	0.744824	0.281133
7	1	0	1.313541	-1.969631	-0.803396
8	7	0	1.072691	1.041406	0.380439

9	7	0	1.784739	-0.028217	-0.039134
10	6	0	3.205831	0.013230	-0.054583
11	6	0	3.846524	1.226313	-0.288531
12	6	0	3.931841	-1.153310	0.163956
13	6	0	5.234001	1.271930	-0.304104
14	1	0	3.262747	2.123466	-0.455788
15	6	0	5.319895	-1.111270	0.132381
16	1	0	3.426085	-2.089117	0.375888
17	6	0	5.957814	0.101874	-0.099759
18	1	0	5.746514	2.209677	-0.483952
19	1	0	5.897808	-2.012193	0.301077
20	17	0	7.704419	0.156895	-0.132126
21	5	0	-3.688968	0.322051	-0.153614
22	6	0	-3.607284	-0.109321	1.530236
23	6	0	-3.316707	1.897820	-0.330038
24	1	0	-2.278652	2.057494	-0.013514
25	1	0	-3.374735	2.149850	-1.398193
26	6	0	-3.200115	-1.481451	1.643452
27	6	0	-1.889493	-1.866492	1.703531
28	1	0	-3.946618	-2.246725	1.435776
29	1	0	-1.620251	-2.918793	1.708151
30	1	0	-1.144240	-1.172948	2.078986
31	6	0	-4.223246	2.798751	0.445520
32	6	0	-5.195897	3.560447	-0.055997
33	1	0	-4.084111	2.796266	1.528585
34	1	0	-5.835147	4.166099	0.580595
35	1	0	-5.384468	3.605959	-1.126952
36	1	0	-2.896616	0.579753	1.995121
37	1	0	-4.628461	0.083396	1.870251
38	6	0	-5.104150	-0.075321	-0.857459
39	1	0	-4.983717	0.073460	-1.939360
40	6	0	-5.584945	-1.463493	-0.587935
41	6	0	-5.388582	-2.530904	-1.363630
42	1	0	-6.107820	-1.613081	0.359604
43	1	0	-5.739917	-3.519188	-1.078797
44	1	0	-4.877960	-2.447464	-2.320911
45	1	0	-5.857961	0.650772	-0.525989

TS1b

Electronic Energy = -2341.836832
Sum of electronic and zero-point Energies = -2341.495629
Sum of electronic and thermal Enthalpies = -2341.468254
Sum of electronic and thermal Free Energies = -2341.556063

Standard orientation:

Center Number	Atomic Number	Atomic Type	Coordinates (Angstroms) X	Y	Z

1	6	0	2.410512	0.487369	-0.984273
2	8	0	2.853745	0.228386	0.253891
3	1	0	2.988552	1.169594	-1.602065
4	6	0	0.961395	0.569102	-1.080224
5	6	0	0.003513	0.171293	-0.176877
6	1	0	0.052366	-0.260981	0.809100
7	7	0	-1.165826	0.437616	-0.806544
8	7	0	0.329650	1.048216	-2.192159
9	7	0	-0.950580	0.970696	-2.028372
10	6	0	-2.484148	0.228255	-0.337070
11	6	0	-3.188366	1.257177	0.293228
12	6	0	-3.110288	-1.009208	-0.504782
13	6	0	-4.486290	1.066647	0.747996
14	6	0	-4.405923	-1.226298	-0.056978
15	6	0	-5.074001	-0.178395	0.563903
16	1	0	-5.022192	1.872863	1.233454
17	1	0	-4.881098	-2.189639	-0.194282
18	5	0	4.191772	0.069787	0.594686
19	6	0	4.315273	-2.402121	-0.365826
20	1	0	4.383878	-2.968926	0.556976
21	1	0	5.247589	-2.073533	-0.816928
22	6	0	4.473724	-0.281661	2.117055
23	1	0	5.385264	-0.886474	2.192749
24	1	0	4.720272	0.683552	2.591340
25	6	0	5.341304	0.614063	-0.359896
26	1	0	5.185394	0.296617	-1.400533
27	1	0	6.304823	0.177230	-0.066928
28	6	0	3.132570	-2.267321	-1.011660
29	6	0	2.916692	-1.465070	-2.170555
30	1	0	2.243810	-2.677888	-0.533156
31	1	0	1.990313	-1.573949	-2.725671
32	1	0	3.778609	-1.150978	-2.754272
33	6	0	3.342380	-0.930651	2.851829
34	6	0	3.366699	-2.153291	3.379424
35	1	0	2.425869	-0.345811	2.928239
36	1	0	2.501082	-2.575159	3.882725
37	1	0	4.258778	-2.775395	3.333619
38	6	0	5.482479	2.119125	-0.352177
39	6	0	4.650086	2.995147	0.209064
40	1	0	6.357717	2.507411	-0.874237
41	1	0	4.837887	4.063462	0.150513
42	1	0	3.751604	2.691416	0.741875
43	17	0	-2.432116	2.802817	0.521029
44	17	0	-6.700918	-0.435321	1.130940
45	17	0	-2.259773	-2.303972	-1.289654

TS1c

Electronic Energy = -3534.238713
Sum of electronic and zero-point Energies = -3533.878200
Sum of electronic and thermal Enthalpies = -3533.853260
Sum of electronic and thermal Free Energies = -3533.935208

Standard orientation:

Center Number	Atomic Number	Atomic Type	Coordinates (Angstroms) X	Y	Z
1	6	0	-2.414205	-1.060184	-0.569080
2	8	0	-2.838228	0.098098	-0.073730
3	1	0	-3.030298	-1.575680	-1.300749
4	6	0	-0.973108	-1.167649	-0.706431
5	6	0	0.016971	-0.347254	-0.213410
6	1	0	-0.010993	0.576909	0.339356
7	7	0	1.165585	-0.934975	-0.620053
8	7	0	-0.380393	-2.195870	-1.379792
9	7	0	0.905781	-2.054095	-1.325307
10	6	0	2.505268	-0.520437	-0.391482
11	6	0	2.822471	0.175374	0.771568
12	6	0	3.481395	-0.818460	-1.338190
13	6	0	4.130534	0.592149	0.985125
14	1	0	2.062220	0.381395	1.517138
15	6	0	4.791948	-0.416413	-1.116278
16	1	0	3.216297	-1.358359	-2.239327
17	6	0	5.103817	0.290094	0.040071
18	1	0	4.385096	1.136833	1.886723
19	1	0	5.558953	-0.644650	-1.846962
20	5	0	-4.176256	0.398129	0.217693
21	6	0	-5.320720	-0.423014	-0.529135
22	1	0	-5.149688	-1.501783	-0.415739
23	1	0	-5.193330	-0.206126	-1.601494
24	6	0	-4.394705	1.870395	0.782212
25	1	0	-5.364101	1.959118	1.283127
26	1	0	-3.608688	2.110399	1.508775
27	6	0	-4.237937	-0.863373	2.347153
28	1	0	-5.193753	-1.252414	2.009022
29	1	0	-4.271789	-0.048554	3.062832
30	6	0	-6.720241	-0.083293	-0.112802
31	6	0	-7.610113	0.578302	-0.849698
32	1	0	-7.011747	-0.392899	0.891741
33	1	0	-8.603208	0.806143	-0.472544
34	1	0	-7.375870	0.915631	-1.857084
35	6	0	-4.322848	2.836090	-0.371495
36	6	0	-5.355535	3.482554	-0.910260
37	1	0	-3.334498	2.969351	-0.813283
38	1	0	-5.229074	4.140635	-1.765637
39	1	0	-6.361782	3.377660	-0.511554
40	6	0	-3.083282	-1.558913	2.132921
41	6	0	-2.916769	-2.595780	1.187334

42	1	0	-2.173158	-1.183076	2.601167
43	1	0	-1.997218	-3.170974	1.175589
44	1	0	-3.795128	-3.091858	0.782576
45	35	0	6.891840	0.850009	0.334715

TS1d

Electronic Energy = -963.114140
Sum of electronic and zero-point Energies = -962.742258
Sum of electronic and thermal Enthalpies = -962.719390
Sum of electronic and thermal Free Energies = -962.796102

Standard orientation:

Center Number	Atomic Number	Atomic Type	Coordinates (Angstroms) X	Y	Z
1	8	0	-1.864897	-0.565290	-0.780695
2	6	0	-0.801853	-1.170809	-0.443172
3	1	0	-0.701078	-2.201310	-0.793690
4	6	0	0.471162	-0.473287	-0.229193
5	6	0	1.738012	-0.980471	-0.416367
6	7	0	0.585473	0.812893	0.203871
7	1	0	2.097804	-1.932535	-0.772826
8	7	0	1.837641	1.120833	0.289646
9	7	0	2.554802	0.040412	-0.087901
10	6	0	3.978784	0.084786	-0.104516
11	6	0	4.615690	1.276673	-0.437486
12	6	0	4.701334	-1.061911	0.211733
13	6	0	6.005371	1.313781	-0.449876
14	1	0	4.028042	2.153682	-0.682846
15	6	0	6.090841	-1.013109	0.179312
16	1	0	4.186618	-1.973610	0.496029
17	6	0	6.744096	0.172103	-0.148091
18	1	0	6.511050	2.238782	-0.706183
19	1	0	6.661880	-1.902447	0.424314
20	5	0	-2.954902	0.293645	-0.123672
21	6	0	-2.861262	-0.158798	1.551006
22	6	0	-2.640277	1.886209	-0.277797
23	1	0	-1.608881	2.073012	0.046203
24	1	0	-2.699942	2.151615	-1.342843
25	6	0	-2.392499	-1.513478	1.643051
26	6	0	-1.064787	-1.836513	1.691190
27	1	0	-3.104099	-2.310697	1.431997
28	1	0	-0.744188	-2.874194	1.681890
29	1	0	-0.351123	-1.110805	2.067694
30	6	0	-3.577844	2.747294	0.505132
31	6	0	-4.563641	3.495809	0.007620
32	1	0	-3.451488	2.725098	1.589697

33	1	0	-5.225681	4.071418	0.649243
34	1	0	-4.740699	3.559680	-1.064659
35	1	0	-2.182061	0.551724	2.030851
36	1	0	-3.889492	-0.018762	1.896063
37	6	0	-4.360782	-0.138351	-0.829475
38	1	0	-4.256503	0.037004	-1.909347
39	6	0	-4.795082	-1.545840	-0.584260
40	6	0	-4.622175	-2.581341	-1.407904
41	1	0	-5.263581	-1.738468	0.383798
42	1	0	-4.936286	-3.586628	-1.138782
43	1	0	-4.164745	-2.454775	-2.387462
44	1	0	-5.136237	0.554040	-0.474458
45	1	0	7.828436	0.206832	-0.165528

TS1e

Electronic Energy = -1002.432232
Sum of electronic and zero-point Energies = -1002.030126
Sum of electronic and thermal Enthalpies = -1002.006355
Sum of electronic and thermal Free Energies = -1002.085855

Standard orientation:

Center Number	Atomic Number	Atomic Type	Coordinates (Angstroms) X	Y	Z
1	6	0	1.330811	-1.297267	-0.316595
2	8	0	1.754677	-0.164776	-0.753033
3	1	0	2.002257	-2.154345	-0.274863
4	6	0	-0.084138	-1.577743	-0.539163
5	6	0	-1.068691	-0.732654	-1.004153
6	1	0	-1.059353	0.299245	-1.317857
7	7	0	-2.173602	-1.497609	-1.010264
8	7	0	-0.640580	-2.792823	-0.285678
9	7	0	-1.905333	-2.739035	-0.568679
10	5	0	3.051264	0.458535	-0.293149
11	6	0	4.274872	-0.624444	-0.434507
12	1	0	4.284398	-0.965648	-1.481353
13	1	0	4.092207	-1.511453	0.187898
14	6	0	2.793779	0.777259	1.432654
15	1	0	3.726489	0.398194	1.860325
16	1	0	2.707403	1.855526	1.570141
17	6	0	3.289942	1.839076	-1.098142
18	1	0	3.558828	1.609933	-2.143097
19	1	0	4.198350	2.313738	-0.695053
20	6	0	5.613393	-0.061036	-0.087249
21	6	0	6.321403	-0.349106	1.006330
22	1	0	6.015044	0.680441	-0.779509
23	1	0	7.272387	0.132880	1.216230

24	1	0	5.975236	-1.086744	1.728693
25	6	0	1.627890	0.058866	1.855049
26	6	0	1.527763	-1.307408	1.875454
27	1	0	0.691828	0.615888	1.919723
28	1	0	0.602848	-1.793643	2.167831
29	1	0	2.427300	-1.916172	1.920071
30	6	0	2.217435	2.896875	-1.127875
31	6	0	0.984969	2.830761	-0.623073
32	1	0	2.501033	3.830009	-1.620911
33	1	0	0.302083	3.673375	-0.698701
34	1	0	0.613406	1.941575	-0.123126
35	6	0	-3.538545	-1.117142	-1.358710
36	1	0	-4.111573	-2.046724	-1.353549
37	1	0	-3.536050	-0.720267	-2.376102
38	6	0	-4.102168	-0.111533	-0.380834
39	6	0	-4.146131	-0.412968	0.983071
40	6	0	-4.575036	1.121211	-0.825771
41	6	0	-4.658664	0.509146	1.887592
42	1	0	-3.772419	-1.371624	1.333751
43	6	0	-5.094356	2.044033	0.080608
44	1	0	-4.538140	1.363070	-1.884627
45	6	0	-5.135353	1.739986	1.437259
46	1	0	-4.688768	0.268043	2.945573
47	1	0	-5.460932	3.001697	-0.275419
48	1	0	-5.536041	2.459339	2.144607

4a

Electronic Energy = -1422.759851
Sum of electronic and zero-point Energies = -1422.394820
Sum of electronic and thermal Enthalpies = -1422.369875
Sum of electronic and thermal Free Energies = -1422.452487

Standard orientation:

Center Number	Atomic Number	Atomic Type	Coordinates (Angstroms) X	Y	Z
1	8	0	-3.305556	0.309030	0.053822
2	6	0	-2.351726	1.326073	0.347954
3	1	0	-2.300223	1.473402	1.433479
4	6	0	-0.992220	0.933886	-0.152965
5	6	0	0.216911	0.906478	0.493464
6	7	0	-0.804807	0.477671	-1.421926
7	1	0	0.511419	1.140008	1.503794
8	7	0	0.443698	0.173429	-1.589478
9	7	0	1.083150	0.430304	-0.432208
10	6	0	2.474660	0.180691	-0.305174
11	6	0	3.038734	-0.886797	-0.997759

12	6	0	3.252747	1.000874	0.505617
13	6	0	4.399043	-1.136396	-0.877262
14	1	0	2.414693	-1.512499	-1.624581
15	6	0	4.611102	0.741906	0.640581
16	1	0	2.811353	1.848985	1.017545
17	6	0	5.171335	-0.323690	-0.054146
18	1	0	4.851635	-1.963427	-1.411595
19	1	0	5.228077	1.372802	1.269522
20	17	0	6.882620	-0.647235	0.108132
21	5	0	-3.285071	-0.958552	0.544852
22	6	0	-2.876275	2.592355	-0.327898
23	6	0	-2.182524	-1.515246	1.557765
24	1	0	-1.652744	-0.709112	2.079341
25	1	0	-2.686573	-2.121400	2.320531
26	6	0	-2.181663	3.878536	0.016958
27	6	0	-1.303620	4.095209	0.992821
28	1	0	-2.465306	4.719559	-0.614098
29	1	0	-0.883332	5.083364	1.153209
30	1	0	-0.968569	3.316763	1.672487
31	6	0	-1.195055	-2.378530	0.817121
32	6	0	-1.128807	-3.705926	0.893243
33	1	0	-0.518741	-1.861489	0.136569
34	1	0	-0.413085	-4.271877	0.304290
35	1	0	-1.787328	-4.273985	1.546853
36	1	0	-2.837812	2.434754	-1.412260
37	1	0	-3.939700	2.684042	-0.074596
38	6	0	-4.423703	-1.933915	0.028108
39	1	0	-4.905470	-2.415964	0.890056
40	6	0	-5.447388	-1.325866	-0.879837
41	6	0	-6.757291	-1.288050	-0.648469
42	1	0	-5.061855	-0.867888	-1.790002
43	1	0	-7.445576	-0.817828	-1.345014
44	1	0	-7.190888	-1.725682	0.248835
45	1	0	-3.894574	-2.748400	-0.492904

4b

Electronic Energy = -2341.913658
Sum of electronic and zero-point Energies = -2341.569202
Sum of electronic and thermal Enthalpies = -2341.541382
Sum of electronic and thermal Free Energies = -2341.633037

Standard orientation:

Center Number	Atomic Number	Atomic Type	Coordinates (Angstroms) X	Y	Z
1	6	0	-1.115496	0.854967	-1.074187
2	6	0	-0.154479	0.668705	-0.112573

3	1	0	-0.148006	0.791519	0.957962
4	7	0	0.929447	0.254353	-0.809708
5	7	0	-0.571740	0.547834	-2.284923
6	7	0	0.662670	0.187824	-2.130909
7	6	0	2.208763	-0.097389	-0.318460
8	6	0	2.497090	-1.418156	0.033334
9	6	0	3.209277	0.867819	-0.183728
10	6	0	3.750497	-1.778258	0.508650
11	6	0	4.471031	0.533902	0.288844
12	6	0	4.719862	-0.790186	0.627328
13	1	0	3.963001	-2.805209	0.778437
14	1	0	5.239188	1.290801	0.388051
15	17	0	1.268295	-2.635605	-0.120488
16	17	0	6.298432	-1.225304	1.221906
17	17	0	2.874876	2.516981	-0.610857
18	6	0	-2.534058	1.318084	-0.940356
19	8	0	-3.150151	0.692216	0.181119
20	5	0	-3.644210	-0.578181	0.164626
21	6	0	-4.244003	-1.114840	1.529624
22	1	0	-5.250710	-1.509120	1.333630
23	1	0	-3.645872	-2.000025	1.801592
24	6	0	-3.638180	-1.458427	-1.161456
25	1	0	-4.296397	-0.958466	-1.888545
26	1	0	-2.632918	-1.426804	-1.606215
27	6	0	-4.067849	-2.883074	-0.980532
28	6	0	-5.210257	-3.408932	-1.417311
29	1	0	-3.379836	-3.520173	-0.423499
30	1	0	-5.465791	-4.448476	-1.232480
31	1	0	-5.931179	-2.817311	-1.978458
32	6	0	-4.258609	-0.137425	2.663896
33	6	0	-5.349686	0.335717	3.262158
34	1	0	-3.284177	0.218839	2.998638
35	1	0	-5.288245	1.059353	4.070332
36	1	0	-6.346437	0.016954	2.962884
37	6	0	-2.645556	2.838303	-0.742378
38	1	0	-3.711051	3.093041	-0.780753
39	1	0	-2.154221	3.327354	-1.589792
40	6	0	-2.057887	3.309293	0.556104
41	6	0	-0.960156	4.054345	0.658336
42	1	0	-2.577838	2.988429	1.456831
43	1	0	-0.571268	4.365583	1.623953
44	1	0	-0.413627	4.385702	-0.222249
45	1	0	-3.054074	1.051195	-1.866927

4c

Electronic Energy = -3534.318598
Sum of electronic and zero-point Energies = -3533.954620
Sum of electronic and thermal Enthalpies = -3533.929238

Sum of electronic and thermal Free Energies = -3534.014478

Standard orientation:

Center Number	Atomic Number	Atomic Type	Coordinates (Angstroms) X	Y	Z
1	6	0	1.312772	-1.380409	-0.099032
2	6	0	0.292434	-0.479406	0.065782
3	1	0	0.288274	0.572600	0.301068
4	7	0	-0.828568	-1.203319	-0.171770
5	7	0	0.775346	-2.586612	-0.421231
6	7	0	-0.518654	-2.479191	-0.464306
7	6	0	-2.182716	-0.776964	-0.137733
8	6	0	-2.551740	0.280823	0.688401
9	6	0	-3.123142	-1.424929	-0.934178
10	6	0	-3.874821	0.704698	0.709363
11	1	0	-1.821599	0.768725	1.324953
12	6	0	-4.448271	-1.010764	-0.901463
13	1	0	-2.818952	-2.244853	-1.573537
14	6	0	-4.811622	0.053753	-0.084160
15	1	0	-4.168665	1.528395	1.349311
16	1	0	-5.186456	-1.510986	-1.517489
17	35	0	-6.619221	0.628033	-0.050146
18	6	0	2.787877	-1.151383	0.049095
19	1	0	3.285300	-1.367574	-0.903533
20	6	0	3.396041	-2.045112	1.138104
21	1	0	3.174701	-3.085241	0.878237
22	1	0	2.882693	-1.819730	2.080706
23	8	0	2.938335	0.216268	0.397831
24	5	0	3.778991	1.106086	-0.197234
25	6	0	3.634037	2.607933	0.295492
26	1	0	4.594415	2.892268	0.752521
27	1	0	3.536807	3.252376	-0.589492
28	6	0	4.813914	0.671187	-1.324185
29	1	0	5.296640	-0.273449	-1.042961
30	1	0	4.220756	0.449043	-2.226545
31	6	0	5.853687	1.701578	-1.650181
32	6	0	7.155350	1.581594	-1.397492
33	1	0	5.493421	2.622338	-2.109846
34	1	0	7.857604	2.374231	-1.639607
35	1	0	7.564908	0.683101	-0.939510
36	6	0	2.505003	2.848521	1.249850
37	6	0	1.405610	3.553287	0.984523
38	1	0	2.598342	2.375909	2.227340
39	1	0	0.615306	3.673642	1.720661
40	1	0	1.261398	4.044406	0.023887
41	6	0	4.876019	-1.843020	1.290810
42	6	0	5.797135	-2.748371	0.971242
43	1	0	5.193377	-0.877148	1.682390
44	1	0	6.857635	-2.547832	1.095018

45	1	0	5.522939	-3.726180	0.580771

4d

Electronic Energy = -963.186073
Sum of electronic and zero-point Energies = -962.812001
Sum of electronic and thermal Enthalpies = -962.788009
Sum of electronic and thermal Free Energies = -962.869538

Standard orientation:

Center Number	Atomic Number	Atomic Type	Coordinates (Angstroms) X	Y	Z
1	6	0	0.241269	0.965647	-0.251720
2	6	0	1.171855	0.022316	0.107527
3	1	0	1.083175	-0.949381	0.564587
4	7	0	2.351785	0.549790	-0.296166
5	7	0	0.889940	2.001608	-0.846131
6	7	0	2.165654	1.750736	-0.870081
7	6	0	3.656965	-0.007058	-0.186016
8	6	0	3.981251	-0.779651	0.925768
9	6	0	4.585963	0.233344	-1.194839
10	6	0	5.255139	-1.330586	1.018065
11	1	0	3.255043	-0.934226	1.716809
12	6	0	5.860409	-0.310349	-1.080858
13	1	0	4.306594	0.835987	-2.051292
14	6	0	6.196323	-1.095989	0.019241
15	1	0	5.513816	-1.934126	1.881853
16	1	0	6.590035	-0.125709	-1.862431
17	1	0	7.190931	-1.522377	0.099240
18	6	0	-1.241444	0.950639	-0.032528
19	8	0	-1.630106	-0.415911	0.033439
20	5	0	-2.827346	-0.894908	-0.402416
21	6	0	-3.911235	0.054789	-1.081475
22	1	0	-4.033688	0.973342	-0.492436
23	1	0	-3.481986	0.376793	-2.044461
24	6	0	-3.067653	-2.451693	-0.220189
25	1	0	-3.990259	-2.585391	0.362362
26	1	0	-3.302487	-2.856378	-1.217558
27	6	0	-5.247120	-0.582629	-1.321084
28	6	0	-6.379746	-0.250996	-0.704937
29	1	0	-5.267207	-1.396744	-2.046140
30	1	0	-7.312183	-0.768709	-0.911240
31	1	0	-6.415211	0.553028	0.027846
32	6	0	-1.934856	-3.219335	0.388351
33	6	0	-1.968535	-3.849025	1.560742
34	1	0	-1.009888	-3.238035	-0.188528
35	1	0	-1.102035	-4.377071	1.949398

36	1	0	-2.866134	-3.858353	2.176263
37	6	0	-1.654972	1.645993	1.276506
38	1	0	-2.728406	1.483508	1.421744
39	1	0	-1.129615	1.143028	2.098032
40	6	0	-1.355752	3.116964	1.281581
41	6	0	-2.284523	4.065331	1.365598
42	1	0	-0.308022	3.400941	1.197771
43	1	0	-2.021325	5.119125	1.362432
44	1	0	-3.342512	3.822734	1.444168
45	1	0	-1.722747	1.458620	-0.875612

4e

Electronic Energy = -1002.495028
Sum of electronic and zero-point Energies = -1002.091080
Sum of electronic and thermal Enthalpies = -1002.065921
Sum of electronic and thermal Free Energies = -1002.151428

Standard orientation:

Center Number	Atomic Number	Atomic Type	Coordinates (Angstroms) X	Y	Z
1	6	0	0.121776	-0.426698	-0.620183
2	6	0	-0.783825	0.302147	0.115756
3	1	0	-0.925953	0.445944	1.175027
4	7	0	-1.563109	0.893960	-0.811818
5	7	0	-0.156693	-0.242716	-1.936720
6	7	0	-1.181376	0.554488	-2.048604
7	6	0	-2.730222	1.741740	-0.609695
8	1	0	-2.943370	2.186892	-1.584167
9	1	0	-2.458976	2.543264	0.080547
10	6	0	-3.915831	0.957955	-0.092163
11	6	0	-4.346895	-0.186915	-0.767388
12	6	0	-4.591209	1.371486	1.054326
13	6	0	-5.440702	-0.905683	-0.299955
14	1	0	-3.818144	-0.515185	-1.658562
15	6	0	-5.691955	0.654320	1.519978
16	1	0	-4.256609	2.257751	1.587015
17	6	0	-6.117010	-0.485194	0.844782
18	1	0	-5.768013	-1.794756	-0.829945
19	1	0	-6.212006	0.984622	2.413826
20	1	0	-6.971815	-1.046819	1.208577
21	6	0	1.250444	-1.298923	-0.165261
22	1	0	1.855436	-1.542964	-1.044405
23	8	0	2.020701	-0.596351	0.807902
24	5	0	3.247972	-0.051364	0.586562
25	6	0	0.762239	-2.607332	0.468648
26	1	0	0.153287	-2.353716	1.345646

27	1	0	0.111564	-3.112881	-0.252337
28	6	0	3.890240	0.701257	1.829733
29	1	0	4.845913	0.206392	2.060734
30	1	0	3.247723	0.615647	2.712664
31	6	0	4.006019	-0.128496	-0.813871
32	1	0	5.060650	0.133933	-0.673885
33	1	0	3.980682	-1.158912	-1.195958
34	6	0	1.900493	-3.502191	0.866304
35	6	0	2.150527	-4.689666	0.321444
36	1	0	2.558049	-3.121975	1.646654
37	1	0	2.995135	-5.294551	0.639182
38	1	0	1.516316	-5.103168	-0.460041
39	6	0	4.153565	2.145759	1.507789
40	6	0	3.527209	3.182832	2.060591
41	1	0	4.900840	2.336818	0.737528
42	1	0	3.749288	4.203868	1.763518
43	1	0	2.769842	3.046383	2.829976
44	6	0	3.383262	0.799937	-1.823321
45	6	0	3.934907	1.928181	-2.266210
46	1	0	2.393552	0.525749	-2.189534
47	1	0	3.420909	2.566226	-2.979380
48	1	0	4.920276	2.251842	-1.936532

5a

Electronic Energy = -2845.560788
Sum of electronic and zero-point Energies = -2844.824940
Sum of electronic and thermal Enthalpies = -2844.777052
Sum of electronic and thermal Free Energies = -2844.908375

Standard orientation:

Center Number	Atomic Number	Atomic Type	Coordinates (Angstroms) X	Y	Z
1	8	0	0.280466	-2.526363	-1.833545
2	6	0	1.554328	-2.695453	-1.295094
3	6	0	2.107355	-1.497270	-0.561060
4	6	0	3.113879	-0.641883	-0.935480
5	7	0	1.670115	-1.042328	0.650293
6	1	0	3.707478	-0.582589	-1.832424
7	7	0	2.342417	0.007446	1.029138
8	7	0	3.213693	0.259152	0.068750
9	6	0	4.036239	1.418884	0.156308
10	6	0	3.525078	2.551003	0.782195
11	6	0	5.314079	1.400277	-0.388698
12	6	0	4.314602	3.689364	0.866180
13	1	0	2.521074	2.535347	1.193570
14	6	0	6.098070	2.544797	-0.316375

15	1	0	5.707204	0.502297	-0.852767
16	6	0	5.590768	3.676588	0.312208
17	1	0	3.935538	4.581471	1.350476
18	1	0	7.097466	2.550163	-0.734859
19	17	0	6.579635	5.114086	0.409824
20	5	0	-0.431177	-1.371621	-2.345831
21	6	0	-1.461840	-1.906970	-3.506448
22	1	0	-2.116655	-1.083243	-3.818643
23	1	0	-0.843790	-2.179996	-4.373154
24	6	0	-2.274956	-3.078802	-3.068205
25	6	0	-3.548637	-3.037024	-2.670471
26	1	0	-1.745521	-4.029712	-3.007416
27	1	0	-4.066773	-3.922145	-2.311012
28	1	0	-4.122102	-2.111665	-2.704522
29	6	0	0.414900	-0.056744	-2.865000
30	1	0	-0.038184	0.308189	-3.795620
31	6	0	0.603445	1.117502	-1.949857
32	6	0	0.423830	2.399614	-2.268634
33	1	0	0.895426	0.905299	-0.923148
34	1	0	0.586946	3.191195	-1.541297
35	1	0	0.111854	2.702764	-3.266168
36	1	0	1.406487	-0.431978	-3.160183
37	8	0	-0.104148	-2.758804	1.124576
38	6	0	-1.351947	-2.838596	0.499595
39	6	0	-1.911622	-1.532367	-0.010314
40	6	0	-2.939075	-0.760722	0.473310
41	7	0	-1.467505	-0.890628	-1.131900
42	1	0	-3.541073	-0.855294	1.361424
43	7	0	-2.155481	0.192340	-1.360979
44	7	0	-3.042531	0.278708	-0.386298
45	6	0	-3.895928	1.416432	-0.316382
46	6	0	-3.408239	2.644757	-0.749818
47	6	0	-5.184437	1.279617	0.184927
48	6	0	-4.231439	3.760208	-0.681293
49	1	0	-2.396373	2.720470	-1.133333
50	6	0	-6.003010	2.399085	0.265881
51	1	0	-5.557549	0.310680	0.498332
52	6	0	-5.518406	3.627269	-0.169894
53	1	0	-3.869942	4.725906	-1.014259
54	1	0	-7.011067	2.312275	0.653651
55	17	0	-6.550163	5.034859	-0.073870
56	5	0	0.604738	-1.679423	1.781230
57	6	0	-0.228843	-0.421454	2.446160
58	1	0	-1.231571	-0.804680	2.678279
59	1	0	0.214388	-0.192621	3.423810
60	6	0	-0.390511	0.866051	1.694943
61	6	0	-0.181197	2.091352	2.178553
62	1	0	-0.701483	0.798716	0.654110
63	1	0	-0.338880	2.975938	1.566016
64	1	0	0.145913	2.252782	3.203881
65	6	0	1.624195	-2.371672	2.865402

66	1	0	2.277716	-1.608397	3.307250
67	6	0	0.860466	-3.085010	3.930447
68	6	0	0.681713	-2.671309	5.186360
69	1	0	0.366844	-4.004739	3.614382
70	1	0	0.069111	-3.229008	5.889747
71	1	0	1.149628	-1.761346	5.557382
72	1	0	2.265662	-3.091981	2.337076
73	6	0	2.522738	-3.178890	-2.393848
74	1	0	1.986505	-3.964772	-2.932709
75	1	0	2.693358	-2.365533	-3.111796
76	6	0	-2.360341	-3.607129	1.376877
77	1	0	-3.287106	-3.747716	0.807829
78	1	0	-1.915288	-4.599923	1.516406
79	1	0	1.473974	-3.494362	-0.549821
80	1	0	-1.213750	-3.446009	-0.399126
81	6	0	3.824663	-3.715948	-1.870866
82	6	0	4.157490	-5.003535	-1.875410
83	1	0	4.523095	-2.998180	-1.441309
84	1	0	5.103694	-5.349028	-1.469388
85	1	0	3.493975	-5.760430	-2.287884
86	6	0	-2.651210	-3.035403	2.733089
87	6	0	-3.870704	-2.776276	3.201691
88	1	0	-1.787569	-2.860835	3.372370
89	1	0	-4.023179	-2.392459	4.206241
90	1	0	-4.762783	-2.955247	2.603245

5b

Electronic Energy = -4683.854462
Sum of electronic and zero-point Energies = -4683.158905
Sum of electronic and thermal Enthalpies = -4683.105312
Sum of electronic and thermal Free Energies = -4683.252259

Standard orientation:

Center Number	Atomic Number	Atomic Type	Coordinates (Angstroms) X	Y	Z
1	6	0	1.917284	1.065176	0.688830
2	6	0	2.640624	0.057817	1.273066
3	1	0	2.761019	-0.251503	2.297461
4	7	0	3.263599	-0.561042	0.243594
5	7	0	2.150339	0.970349	-0.653940
6	7	0	2.966975	-0.006676	-0.922796
7	6	0	4.173069	-1.649973	0.293103
8	6	0	5.536136	-1.417707	0.485399
9	6	0	3.716665	-2.960753	0.149000
10	6	0	6.438056	-2.471337	0.533640
11	6	0	4.600048	-4.029961	0.194676

12	6	0	5.950236	-3.764378	0.387239
13	1	0	7.494893	-2.284196	0.679273
14	1	0	4.240502	-5.045094	0.081638
15	17	0	6.113569	0.208565	0.664859
16	17	0	7.067345	-5.097797	0.448168
17	17	0	2.025898	-3.262557	-0.089709
18	6	0	1.030358	2.088948	1.345059
19	8	0	0.005200	1.425516	2.019490
20	5	0	-1.386997	1.834499	1.948550
21	6	0	-2.146644	1.253314	3.268644
22	1	0	-3.227305	1.431112	3.216577
23	1	0	-1.998084	0.166031	3.316673
24	6	0	-1.698471	3.425909	1.708472
25	1	0	-1.491427	3.920829	2.665995
26	1	0	-1.028942	3.894592	0.974052
27	6	0	-3.114766	3.700616	1.307983
28	6	0	-4.073768	4.180632	2.099685
29	1	0	-3.382310	3.441143	0.281084
30	1	0	-5.092178	4.316871	1.745483
31	1	0	-3.869447	4.452668	3.133244
32	6	0	-1.590774	1.868716	4.512449
33	6	0	-2.237551	2.685607	5.345136
34	1	0	-0.545834	1.638294	4.724657
35	1	0	-1.752163	3.115805	6.217492
36	1	0	-3.281400	2.948865	5.183735
37	6	0	1.821691	3.012578	2.293910
38	1	0	1.108646	3.772504	2.636101
39	1	0	2.603737	3.526988	1.723420
40	6	0	2.410938	2.315240	3.482147
41	6	0	3.712057	2.262965	3.758349
42	1	0	1.700770	1.819945	4.141752
43	1	0	4.087512	1.747441	4.638091
44	1	0	4.449576	2.742292	3.117419
45	1	0	0.650076	2.729341	0.551894
46	6	0	-1.931491	1.105409	-0.732373
47	6	0	-2.761524	0.160291	-1.279665
48	1	0	-2.981996	-0.108250	-2.298808
49	7	0	-3.343529	-0.449277	-0.222016
50	7	0	-2.076646	0.990067	0.626658
51	7	0	-2.929225	0.054686	0.928670
52	6	0	-4.329038	-1.471371	-0.226575
53	6	0	-5.684159	-1.136206	-0.198457
54	6	0	-3.957840	-2.816201	-0.252315
55	6	0	-6.660494	-2.122110	-0.200372
56	6	0	-4.917374	-3.818968	-0.255973
57	6	0	-6.257181	-3.451553	-0.230317
58	1	0	-7.709774	-1.856017	-0.177296
59	1	0	-4.623215	-4.860995	-0.275007
60	17	0	-6.158670	0.531880	-0.155928
61	17	0	-7.471653	-4.698118	-0.232978
62	17	0	-2.277620	-3.245863	-0.273746

63	6	0	-1.010494	2.081958	-1.430965
64	8	0	0.065803	1.372541	-1.967168
65	5	0	1.456532	1.812742	-1.953043
66	6	0	1.750567	3.407832	-1.730801
67	1	0	1.475007	3.910021	-2.666737
68	1	0	1.129038	3.865996	-0.948796
69	6	0	2.194563	1.228772	-3.283702
70	1	0	3.286012	1.299896	-3.206675
71	1	0	1.947422	0.161195	-3.375389
72	6	0	1.732205	1.941772	-4.514827
73	6	0	2.500441	2.643425	-5.349504
74	1	0	0.660192	1.897506	-4.714294
75	1	0	2.085386	3.156809	-6.213357
76	1	0	3.575139	2.726896	-5.197497
77	6	0	3.188385	3.696040	-1.424722
78	6	0	4.065615	4.269193	-2.248282
79	1	0	3.545129	3.365398	-0.446392
80	1	0	5.104986	4.412884	-1.964872
81	1	0	3.771340	4.609250	-3.238947
82	6	0	-1.738987	2.936758	-2.481272
83	1	0	-1.041113	3.731614	-2.777698
84	1	0	-2.569383	3.450021	-1.979553
85	6	0	-2.254988	2.286020	-3.738743
86	6	0	-1.875686	1.130106	-4.282432
87	1	0	-3.004743	2.879421	-4.262335
88	1	0	-2.315629	0.786956	-5.214885
89	1	0	-1.115334	0.506860	-3.822634
90	1	0	-0.689449	2.791848	-0.666466

6a

Electronic Energy = -2845.565578
Sum of electronic and zero-point Energies = -2844.830118
Sum of electronic and thermal Enthalpies = -2844.782242
Sum of electronic and thermal Free Energies = -2844.913005

Standard orientation:

Center Number	Atomic Number	Atomic Type	Coordinates (Angstroms) X	Y	Z
1	8	0	-0.046758	-2.428647	-1.747025
2	6	0	1.303183	-2.682744	-1.617947
3	6	0	2.050815	-1.656894	-0.798141
4	6	0	3.169667	-0.969950	-1.195446
5	7	0	1.738861	-1.179405	0.444303
6	1	0	3.719688	-0.977894	-2.122156
7	7	0	2.592131	-0.263967	0.814166
8	7	0	3.456050	-0.128621	-0.174151

9	6	0	4.458030	0.881108	-0.094278
10	6	0	4.147066	2.078651	0.541051
11	6	0	5.708686	0.655896	-0.655532
12	6	0	5.114261	3.071132	0.619790
13	1	0	3.157511	2.225885	0.961370
14	6	0	6.671382	1.655133	-0.588895
15	1	0	5.941725	-0.292403	-1.127078
16	6	0	6.364751	2.851028	0.050868
17	1	0	4.892994	4.011311	1.111183
18	1	0	7.653060	1.498621	-1.019896
19	17	0	7.578540	4.104899	0.142658
20	5	0	-0.709675	-1.286986	-2.356843
21	6	0	-1.829155	-1.834730	-3.424207
22	1	0	-2.468152	-1.005647	-3.755661
23	1	0	-1.276834	-2.192930	-4.304853
24	6	0	-2.655899	-2.940240	-2.857271
25	6	0	-3.911781	-2.827363	-2.419086
26	1	0	-2.142745	-3.893538	-2.727124
27	1	0	-4.436664	-3.662944	-1.963454
28	1	0	-4.463495	-1.893069	-2.513203
29	6	0	0.211966	-0.091261	-3.011305
30	1	0	-0.316573	0.324577	-3.878307
31	6	0	0.664052	1.048013	-2.148202
32	6	0	0.480306	2.344566	-2.399992
33	1	0	1.168854	0.796676	-1.217765
34	1	0	0.840604	3.109954	-1.717157
35	1	0	-0.029730	2.684848	-3.299205
36	1	0	1.101762	-0.589783	-3.428718
37	8	0	-0.264897	-2.600816	1.126802
38	6	0	-1.553894	-2.545884	0.589715
39	6	0	-2.002573	-1.200205	0.066215
40	6	0	-2.880175	-0.292467	0.605800
41	7	0	-1.611670	-0.659718	-1.127315
42	1	0	-3.395879	-0.279443	1.551088
43	7	0	-2.193134	0.485088	-1.346572
44	7	0	-2.955283	0.715958	-0.292812
45	6	0	-3.652472	1.953280	-0.188840
46	6	0	-3.058978	3.099293	-0.707123
47	6	0	-4.893524	1.993027	0.434196
48	6	0	-3.726907	4.311533	-0.603087
49	1	0	-2.084297	3.036923	-1.179242
50	6	0	-5.555217	3.208956	0.550418
51	1	0	-5.351730	1.086757	0.814745
52	6	0	-4.965853	4.354931	0.028228
53	1	0	-3.281531	5.216054	-0.999954
54	1	0	-6.524146	3.259881	1.032612
55	17	0	-5.800539	5.884407	0.168306
56	5	0	0.638082	-1.613977	1.664777
57	6	0	0.044983	-0.211259	2.295658
58	1	0	-0.977188	-0.433977	2.630648
59	1	0	0.598868	0.014176	3.215960

60	6	0	0.000397	1.031824	1.459804
61	6	0	0.440360	2.238438	1.819679
62	1	0	-0.418771	0.944941	0.459492
63	1	0	0.357385	3.093063	1.152543
64	1	0	0.887634	2.415432	2.796111
65	6	0	1.618229	-2.395539	2.725588
66	1	0	2.395478	-1.714533	3.094649
67	6	0	0.832231	-2.953533	3.864470
68	6	0	0.793300	-2.471610	5.108069
69	1	0	0.197513	-3.806340	3.620979
70	1	0	0.156169	-2.910683	5.871189
71	1	0	1.404467	-1.622434	5.408041
72	1	0	2.123651	-3.218394	2.198846
73	6	0	-2.574445	-3.155181	1.571686
74	1	0	-3.550888	-3.212448	1.076088
75	1	0	-2.234070	-4.186226	1.729374
76	1	0	-1.553772	-3.198210	-0.287029
77	6	0	-2.697550	-2.497463	2.914758
78	6	0	-3.844256	-2.096879	3.461432
79	1	0	-1.771634	-2.381474	3.475381
80	1	0	-3.876732	-1.656216	4.453665
81	1	0	-4.795011	-2.211452	2.942975
82	6	0	1.473845	-4.093812	-1.031011
83	1	0	2.548356	-4.310021	-0.961073
84	1	0	1.052443	-4.092967	-0.022152
85	1	0	1.802516	-2.664898	-2.603341
86	6	0	0.795498	-5.127810	-1.879392
87	6	0	-0.145794	-5.959274	-1.442488
88	1	0	1.111146	-5.181319	-2.922240
89	1	0	-0.602856	-6.697567	-2.095094
90	1	0	-0.489827	-5.931595	-0.410925

6b

Electronic Energy = -4683.864207
Sum of electronic and zero-point Energies = -4683.115917
Sum of electronic and thermal Enthalpies = -4683.114972
Sum of electronic and thermal Free Energies = -4683.260080

Standard orientation:

Center Number	Atomic Number	Atomic Type	Coordinates (Angstroms) X	Y	Z
1	6	0	1.925843	0.007887	1.057268
2	6	0	3.243197	-0.348549	1.196394
3	1	0	3.765897	-0.942707	1.928074
4	7	0	3.878309	0.230000	0.153023
5	7	0	1.866630	0.780176	-0.073921

6	7	0	3.045055	0.908890	-0.614576
7	6	0	5.257861	0.171606	-0.180501
8	6	0	6.153732	1.101495	0.348360
9	6	0	5.726463	-0.820967	-1.042811
10	6	0	7.503128	1.047344	0.028773
11	6	0	7.071651	-0.893495	-1.376015
12	6	0	7.938626	0.045734	-0.830708
13	1	0	8.196281	1.771228	0.439093
14	1	0	7.430879	-1.664353	-2.046507
15	17	0	5.576273	2.343813	1.413051
16	17	0	9.628500	-0.033028	-1.239388
17	17	0	4.611615	-1.967715	-1.709828
18	6	0	0.769552	-0.439052	1.927781
19	8	0	0.240225	-1.606783	1.361846
20	5	0	-1.158907	-2.005047	1.459413
21	6	0	-1.959507	-1.749344	2.867823
22	1	0	-2.945551	-2.221500	2.775596
23	1	0	-1.418890	-2.344080	3.617589
24	6	0	-1.242165	-3.565694	0.979024
25	1	0	-2.289016	-3.880043	0.872926
26	1	0	-0.776140	-3.641739	-0.015647
27	6	0	-0.521653	-4.468876	1.925872
28	6	0	-1.080838	-5.378213	2.725236
29	1	0	0.556978	-4.317091	1.984713
30	1	0	-0.490515	-5.969625	3.420394
31	1	0	-2.153806	-5.561859	2.712754
32	6	0	-2.136517	-0.352318	3.381480
33	6	0	-3.264902	0.360024	3.369372
34	1	0	-1.256965	0.129935	3.808984
35	1	0	-3.298915	1.373820	3.759680
36	1	0	-4.190687	-0.044782	2.967226
37	6	0	-1.630054	-0.742459	-0.955782
38	6	0	-2.743877	-0.181736	-1.524839
39	1	0	-2.905678	0.313722	-2.468340
40	7	0	-3.720119	-0.339294	-0.600314
41	7	0	-2.014708	-1.182893	0.276750
42	7	0	-3.278853	-0.949869	0.487625
43	6	0	-5.079975	0.065100	-0.667954
44	6	0	-6.050495	-0.811626	-1.155925
45	6	0	-5.460387	1.338321	-0.238060
46	6	0	-7.384252	-0.433688	-1.220158
47	6	0	-6.789283	1.736611	-0.294311
48	6	0	-7.729859	0.840600	-0.786651
49	1	0	-8.133588	-1.118882	-1.596935
50	1	0	-7.080751	2.723953	0.042016
51	17	0	-5.588320	-2.398193	-1.686712
52	17	0	-9.398478	1.330607	-0.862085
53	17	0	-4.267867	2.434476	0.375307
54	6	0	-0.242779	-0.820744	-1.540688
55	1	0	0.408761	-1.278125	-0.790091
56	6	0	-0.251327	-1.723150	-2.784055

57	1	0	-0.693832	-2.692415	-2.528252
58	1	0	-0.898203	-1.250631	-3.535964
59	8	0	0.202495	0.447392	-1.921994
60	5	0	0.602905	1.468834	-0.959297
61	6	0	1.275780	2.681647	-1.827240
62	1	0	1.815977	3.395344	-1.192124
63	1	0	2.013399	2.241733	-2.510427
64	6	0	-0.550590	2.015093	0.063091
65	1	0	-1.123515	1.249370	0.594552
66	1	0	-1.269770	2.498282	-0.615771
67	6	0	1.124132	-1.909027	-3.352804
68	6	0	1.687252	-3.091244	-3.590040
69	1	0	1.668607	-0.992820	-3.573417
70	1	0	2.681148	-3.171440	-4.022041
71	1	0	1.170011	-4.023910	-3.373503
72	6	0	-0.080125	3.041138	1.047131
73	6	0	-0.318601	3.030549	2.359579
74	1	0	0.518056	3.857720	0.640328
75	1	0	0.071603	3.801475	3.018959
76	1	0	-0.926025	2.253170	2.818204
77	6	0	0.243820	3.399234	-2.636436
78	6	0	-0.122245	4.674489	-2.500653
79	1	0	-0.270571	2.789457	-3.381243
80	1	0	-0.906693	5.113388	-3.111929
81	1	0	0.353254	5.328039	-1.771216
82	6	0	1.271244	-0.704000	3.361332
83	1	0	0.461581	-1.184543	3.913266
84	1	0	2.083246	-1.440868	3.316562
85	6	0	1.709617	0.540928	4.077593
86	6	0	1.178443	0.976659	5.217189
87	1	0	2.501794	1.125031	3.608780
88	1	0	1.523030	1.891554	5.690592
89	1	0	0.382001	0.431613	5.719669
90	1	0	0.035928	0.368733	1.962080

7a

Electronic Energy = -2468.773899
Sum of electronic and zero-point Energies = -2468.263631
Sum of electronic and thermal Enthalpies = -2468.227264
Sum of electronic and thermal Free Energies = -2468.336286

Standard orientation:

Center Number	Atomic Number	Atomic Type	Coordinates (Angstroms) X	Y	Z
1	8	0	-1.492891	1.965640	0.981781
2	6	0	-2.011433	2.420974	-0.246191

3	6	0	-3.168645	1.572137	-0.710981
4	6	0	-4.259137	1.166780	0.018251
5	7	0	-3.282327	1.046537	-1.960611
6	1	0	-4.564805	1.361922	1.033058
7	7	0	-4.370479	0.343675	-2.040807
8	7	0	-4.977239	0.403906	-0.840214
9	6	0	-6.199333	-0.280929	-0.617763
10	6	0	-7.084882	-0.456693	-1.677745
11	6	0	-6.498338	-0.770986	0.650103
12	6	0	-8.281432	-1.128275	-1.466509
13	1	0	-6.833079	-0.070778	-2.658083
14	6	0	-7.702552	-1.429059	0.867299
15	1	0	-5.791355	-0.658503	1.464647
16	6	0	-8.582409	-1.602662	-0.194339
17	1	0	-8.977845	-1.271566	-2.284376
18	1	0	-7.945341	-1.814150	1.850761
19	17	0	-10.096970	-2.437206	0.074909
20	5	0	-0.860754	0.718824	1.242879
21	6	0	-1.375806	-0.637420	0.468203
22	1	0	-2.413121	-0.802316	0.787266
23	1	0	-1.399047	-0.473332	-0.614614
24	6	0	-0.559956	-1.842621	0.786230
25	6	0	0.435128	-2.328172	0.035761
26	1	0	-0.761852	-2.327387	1.742559
27	1	0	1.014576	-3.193694	0.345545
28	1	0	0.673321	-1.897921	-0.934970
29	6	0	-0.671966	0.575924	2.855965
30	1	0	-0.282808	-0.401176	3.171487
31	6	0	0.153912	1.672907	3.446806
32	6	0	1.270058	1.514019	4.159687
33	1	0	-0.176569	2.684099	3.211405
34	1	0	1.843190	2.362410	4.523063
35	1	0	1.639892	0.524564	4.427051
36	1	0	-1.695416	0.635749	3.253396
37	8	0	0.685169	0.842853	0.561767
38	6	0	1.642921	0.202157	1.003022
39	1	0	1.588759	-0.284056	1.980097
40	6	0	2.898164	0.110037	0.293223
41	6	0	4.082576	-0.422387	0.758953
42	1	0	4.358165	-0.853999	1.708145
43	7	0	3.100460	0.561967	-0.980262
44	7	0	4.320157	0.340591	-1.313627
45	7	0	4.940880	-0.257871	-0.260560
46	6	0	6.320083	-0.601099	-0.343867
47	6	0	7.162530	0.180556	-1.128509
48	6	0	6.802516	-1.702569	0.355507
49	6	0	8.509340	-0.145215	-1.212624
50	1	0	6.766795	1.033284	-1.666875
51	6	0	8.153002	-2.019052	0.284128
52	1	0	6.134156	-2.325315	0.939854
53	6	0	8.993492	-1.237416	-0.500332

54	1	0	9.177226	0.452901	-1.821174
55	1	0	8.542917	-2.874346	0.823097
56	17	0	10.691202	-1.637675	-0.594507
57	1	0	-2.425767	3.413583	-0.017288
58	6	0	-0.955414	2.629588	-1.338821
59	1	0	-0.435027	1.693392	-1.557933
60	1	0	-1.487196	2.919109	-2.254080
61	6	0	0.018999	3.710105	-0.970454
62	6	0	1.342848	3.604124	-1.020340
63	1	0	-0.426090	4.656023	-0.656193
64	1	0	1.984089	4.442497	-0.760774
65	1	0	1.830075	2.679773	-1.320078

7b

Electronic Energy = -4307.073275
Sum of electronic and zero-point Energies = -4306.604291
Sum of electronic and thermal Enthalpies = -4306.562015
Sum of electronic and thermal Free Energies = -4306.687560

Standard orientation:

Center Number	Atomic Number	Atomic Type	Coordinates (Angstroms) X	Y	Z
1	6	0	-4.146789	0.840329	0.232542
2	6	0	-3.077548	1.253808	-0.518035
3	7	0	-4.262350	-0.027698	-1.816031
4	7	0	-4.857862	0.046908	-0.605507
5	6	0	-6.052113	-0.665320	-0.349421
6	6	0	-7.297255	-0.084014	-0.600584
7	6	0	-6.009751	-1.970586	0.147839
8	6	0	-8.476767	-0.777778	-0.367002
9	6	0	-7.174332	-2.686745	0.388902
10	6	0	-8.392614	-2.074172	0.124791
11	1	0	-9.435672	-0.315778	-0.566864
12	1	0	-7.128261	-3.698883	0.771231
13	6	0	-1.911289	2.119288	-0.122829
14	8	0	-0.710721	1.401393	-0.209142
15	5	0	-0.293491	0.543109	0.874060
16	6	0	-0.289920	1.216515	2.358290
17	1	0	-1.321885	1.246221	2.735153
18	1	0	0.257516	0.545675	3.033999
19	6	0	-0.893239	-0.967475	0.801267
20	1	0	-0.819876	-1.312993	-0.239525
21	1	0	-1.965330	-0.890450	1.025565
22	6	0	-0.260085	-1.958851	1.722203
23	6	0	-0.803500	-2.449349	2.837076
24	1	0	0.753602	-2.271217	1.464820

25	1	0	-0.267555	-3.142779	3.479839
26	1	0	-1.810365	-2.172466	3.145399
27	6	0	0.312492	2.584412	2.398187
28	6	0	-0.327378	3.727885	2.647676
29	1	0	1.378794	2.641243	2.167514
30	1	0	0.184275	4.686315	2.623532
31	1	0	-1.388382	3.745848	2.891080
32	6	0	4.202638	-0.007960	0.023142
33	6	0	3.289906	0.838878	-0.576079
34	6	0	1.885868	1.008512	-0.330375
35	8	0	1.310585	0.344891	0.544440
36	1	0	1.322193	1.732989	-0.924219
37	7	0	5.176644	1.241206	-1.552054
38	7	0	5.352740	0.268251	-0.605162
39	6	0	6.636123	-0.301042	-0.399326
40	6	0	7.041556	-1.416185	-1.136058
41	6	0	7.515795	0.258128	0.530222
42	6	0	8.298760	-1.973706	-0.950149
43	6	0	8.777563	-0.282623	0.732350
44	6	0	9.147349	-1.394292	-0.014755
45	1	0	8.607123	-2.839023	-1.523464
46	1	0	9.454498	0.155647	1.455013
47	1	0	-4.446691	1.028199	1.251443
48	1	0	4.117021	-0.745469	0.806334
49	7	0	-3.194309	0.699760	-1.759666
50	7	0	3.941628	1.575869	-1.528896
51	17	0	-4.476804	-2.715192	0.475876
52	17	0	-9.863061	-2.960833	0.421691
53	17	0	-7.378801	1.538115	-1.215606
54	17	0	5.963184	-2.119557	-2.298716
55	17	0	7.029681	1.647298	1.449320
56	17	0	10.726056	-2.083781	0.229446
57	6	0	-1.783430	3.366667	-1.007009
58	1	0	-0.909099	3.921260	-0.651266
59	1	0	-1.584847	3.034252	-2.032854
60	6	0	-3.007505	4.234154	-0.962506
61	6	0	-3.077134	5.399135	-0.323388
62	1	0	-3.891498	3.851492	-1.472336
63	1	0	-3.991591	5.985559	-0.303553
64	1	0	-2.216294	5.810169	0.200695
65	1	0	-2.093287	2.459805	0.904987

7a′

Electronic Energies = -2468.788088
Sum of electronic and zero-point Energies = -2468.279637
Sum of electronic and thermal Enthalpies = -2468.242119
Sum of electronic and thermal Free Energies = -2468.353322

Standard orientation:

Center Number	Atomic Number	Atomic Type	Coordinates (Angstroms) X	Y	Z
1	8	0	4.875874	-0.240267	-0.307361
2	6	0	4.446568	1.089882	-0.045192
3	6	0	3.000989	1.245644	-0.411939
4	6	0	1.925103	1.544389	0.381480
5	7	0	2.552837	1.068119	-1.686516
6	1	0	1.853680	1.766076	1.433420
7	7	0	1.271348	1.231071	-1.715415
8	7	0	0.862210	1.523594	-0.463801
9	6	0	-0.514904	1.752221	-0.199762
10	6	0	-0.948156	2.014123	1.097583
11	6	0	-1.427905	1.690758	-1.251733
12	6	0	-2.300137	2.221026	1.346130
13	6	0	-2.776430	1.905976	-1.004415
14	6	0	-3.203477	2.168678	0.292419
15	1	0	-2.640472	2.428418	2.354160
16	1	0	-3.489087	1.864481	-1.819701
17	17	0	-4.902670	2.439369	0.602784
18	5	0	4.648714	-1.303125	0.513171
19	6	0	4.030245	-1.158350	1.980210
20	1	0	3.248471	-0.389773	2.014701
21	1	0	3.575385	-2.110971	2.274644
22	6	0	5.133191	-0.806071	2.940258
23	6	0	5.715560	-1.651588	3.789384
24	1	0	5.503897	0.218900	2.891732
25	1	0	6.535856	-1.339550	4.429438
26	1	0	5.390334	-2.686661	3.873343
27	6	0	5.228520	-2.707303	0.057289
28	1	0	4.422231	-3.447794	0.133056
29	6	0	5.855341	-2.747485	-1.301713
30	6	0	5.400997	-3.446682	-2.339226
31	1	0	6.740305	-2.125732	-1.437747
32	1	0	5.890481	-3.411400	-3.308643
33	1	0	4.511566	-4.068820	-2.260106
34	1	0	5.967570	-2.992523	0.823882
35	8	0	2.111906	-1.904709	-0.460456
36	6	0	1.153050	-1.674327	0.246044
37	1	0	1.270861	-1.472557	1.328540
38	6	0	-0.230869	-1.636913	-0.227774
39	6	0	-1.369474	-1.433783	0.521944
40	1	0	-1.519144	-1.256518	1.574894
41	7	0	-0.596741	-1.774468	-1.536245
42	7	0	-1.876444	-1.677498	-1.624291
43	7	0	-2.370799	-1.470483	-0.375814
44	6	0	-3.773342	-1.333467	-0.177142
45	6	0	-4.322436	-1.536612	1.084761
46	6	0	-4.577981	-0.990953	-1.260812

**47**	**6**	**0**	**-5.690905**	**-1.382620**	**1.270142**
**48**	**6**	**0**	**-5.946530**	**-0.849340**	**-1.079006**
**49**	**6**	**0**	**-6.489857**	**-1.041256**	**0.186297**
**50**	**1**	**0**	**-6.127324**	**-1.536368**	**2.250156**
**51**	**1**	**0**	**-6.580728**	**-0.582221**	**-1.916213**
**52**	**17**	**0**	**-8.212378**	**-0.844707**	**0.419412**
**53**	**1**	**0**	**4.556737**	**1.328605**	**1.019498**
**54**	**6**	**0**	**5.358302**	**2.018506**	**-0.855264**
**55**	**1**	**0**	**6.391498**	**1.820217**	**-0.553601**
**56**	**1**	**0**	**5.256411**	**1.741167**	**-1.910727**
**57**	**6**	**0**	**5.022807**	**3.469109**	**-0.663546**
**58**	**6**	**0**	**5.800097**	**4.344907**	**-0.032390**
**59**	**1**	**0**	**4.068638**	**3.800601**	**-1.072177**
**60**	**1**	**0**	**5.509512**	**5.385579**	**0.080616**
**61**	**1**	**0**	**6.762044**	**4.054541**	**0.385404**
**62**	**1**	**0**	**-4.136204**	**-0.838043**	**-2.237483**
**63**	**1**	**0**	**-3.703729**	**-1.827786**	**1.926101**
**64**	**1**	**0**	**-1.083962**	**1.470393**	**-2.254009**
**65**	**1**	**0**	**-0.251904**	**2.060748**	**1.926608**

7b′

Electronic Energies = -4307.070542
Sum of electronic and zero-point Energies = -4306.601852
Sum of electronic and thermal Enthalpies = -4306.559671
Sum of electronic and thermal Free Energies = -4306.682298

Standard orientation:

Center Number	Atomic Number	Atomic Type	Coordinates (Angstroms) X	Y	Z
1	8	0	4.252153	-2.045350	-0.385923
2	6	0	4.755349	-0.729237	-0.415711
3	6	0	3.652447	0.267105	-0.642424
4	6	0	3.147175	1.218741	0.199772
5	7	0	2.900231	0.268905	-1.782601
6	1	0	3.435056	1.552431	1.183691
7	7	0	1.962851	1.147661	-1.686281
8	7	0	2.093251	1.742991	-0.477068
9	6	0	1.172440	2.735328	-0.075862
10	6	0	0.339216	2.554313	1.034904
11	6	0	1.054880	3.929280	-0.798048
12	6	0	-0.603106	3.507295	1.396457
13	6	0	0.110779	4.890464	-0.461851
14	6	0	-0.711973	4.659154	0.631014
15	1	0	-1.240643	3.345799	2.256583
16	1	0	0.030950	5.803515	-1.038553
17	17	0	-1.902984	5.855107	1.066642

18	5	0	3.422557	-2.531290	0.649809
19	6	0	3.619310	-1.972441	2.161410
20	1	0	3.581642	-0.874806	2.176031
21	1	0	2.793246	-2.325123	2.793378
22	6	0	4.922491	-2.442393	2.729398
23	6	0	5.073854	-3.380765	3.664049
24	1	0	5.820298	-2.000577	2.293099
25	1	0	6.056782	-3.702797	3.996778
26	1	0	4.215559	-3.862278	4.129516
27	6	0	3.211817	-4.133640	0.513589
28	1	0	2.636405	-4.548838	1.352442
29	6	0	2.615054	-4.555585	-0.789191
30	6	0	1.470231	-5.223058	-0.946176
31	1	0	3.153708	-4.230359	-1.679169
32	1	0	1.076519	-5.459009	-1.931015
33	1	0	0.893799	-5.574735	-0.091230
34	1	0	4.224257	-4.551310	0.608181
35	8	0	1.843952	-1.826235	0.241273
36	6	0	0.763464	-2.336053	0.527483
37	1	0	0.713270	-3.252182	1.128106
38	6	0	-0.490242	-1.749894	0.116983
39	6	0	-1.763743	-2.187753	0.415055
40	1	0	-2.139357	-3.037028	0.965174
41	7	0	-0.587832	-0.598807	-0.620093
42	7	0	-1.821162	-0.310928	-0.791160
43	7	0	-2.561686	-1.276199	-0.161789
44	6	0	-3.976923	-1.204812	-0.178015
45	6	0	-4.653871	-0.416281	0.755371
46	6	0	-4.712657	-1.891864	-1.146412
47	6	0	-6.037481	-0.313412	0.734859
48	6	0	-6.097494	-1.806070	-1.185176
49	6	0	-6.737497	-1.014116	-0.239735
50	1	0	-6.554359	0.300967	1.461546
51	1	0	-6.660409	-2.340706	-1.940331
52	17	0	-8.473527	-0.894059	-0.277174
53	1	0	5.239516	-0.474306	0.538140
54	6	0	5.816148	-0.677539	-1.523043
55	1	0	6.563147	-1.447318	-1.305277
56	1	0	5.321482	-0.946395	-2.463959
57	6	0	6.466784	0.669032	-1.647132
58	6	0	7.738604	0.924615	-1.351587
59	1	0	5.828566	1.481099	-1.995157
60	1	0	8.158303	1.921480	-1.454808
61	1	0	8.409757	0.142356	-1.002183
62	17	0	-3.755959	0.447304	1.963976
63	17	0	-3.889167	-2.864906	-2.323329
64	17	0	0.470458	1.132699	2.027247
65	17	0	2.127118	4.263349	-2.123231

TS2aS

Electronic Energies = -2468.768922
Sum of electronic and zero-point Energies = -2468.258774
Sum of electronic and thermal Enthalpies = -2468.223649
Sum of electronic and thermal Free Energies = -2468.329438

Standard orientation:

Center Number	Atomic Number	Atomic Type	Coordinates (Angstroms) X	Y	Z
1	8	0	-1.595097	2.948254	-0.254990
2	6	0	-1.901205	2.314424	-1.475281
3	6	0	-2.714821	1.060079	-1.284297
4	6	0	-4.037247	0.949657	-0.931278
5	7	0	-2.193313	-0.197455	-1.345019
6	1	0	-4.813835	1.683550	-0.788744
7	7	0	-3.110975	-1.064253	-1.048792
8	7	0	-4.244332	-0.381197	-0.790307
9	6	0	-5.431533	-1.071177	-0.434270
10	6	0	-5.654577	-2.348005	-0.943068
11	6	0	-6.353447	-0.472101	0.419091
12	6	0	-6.811397	-3.031820	-0.594628
13	1	0	-4.923995	-2.798247	-1.604089
14	6	0	-7.519735	-1.148256	0.755612
15	1	0	-6.161042	0.509738	0.837287
16	6	0	-7.736531	-2.423132	0.246556
17	1	0	-6.994727	-4.026623	-0.983478
18	1	0	-8.244382	-0.691279	1.419358
19	17	0	-9.200746	-3.280404	0.675366
20	5	0	-0.819966	2.437485	0.831867
21	6	0	-1.631358	1.220938	1.775645
22	1	0	-2.439038	1.762739	2.274008
23	1	0	-2.024293	0.489883	1.070713
24	6	0	-0.670506	0.641080	2.681669
25	6	0	0.233097	-0.308818	2.302563
26	1	0	-0.525114	1.120324	3.650200
27	1	0	0.980214	-0.678532	2.999053
28	1	0	0.066009	-0.897445	1.404020
29	6	0	-0.352846	3.688331	1.773193
30	1	0	-0.005402	3.373197	2.766919
31	6	0	0.698159	4.509364	1.100112
32	6	0	1.942008	4.703509	1.541419
33	1	0	0.420428	4.918529	0.129509
34	1	0	2.669980	5.271285	0.968533
35	1	0	2.272684	4.316917	2.504901
36	1	0	-1.254505	4.294170	1.934125
37	8	0	0.369197	1.646355	0.304756
38	6	0	1.313435	1.264107	1.059321
39	1	0	1.588402	1.850141	1.934801

40	6	0	2.353061	0.418464	0.482988
41	6	0	3.608434	0.148917	0.978224
42	1	0	4.127008	0.469049	1.867581
43	7	0	2.220014	-0.207062	-0.721423
44	7	0	3.311454	-0.839645	-0.988996
45	7	0	4.171007	-0.635839	0.036420
46	6	0	5.472996	-1.205419	0.006176
47	6	0	6.113735	-1.362643	-1.219222
48	6	0	6.085034	-1.595605	1.192928
49	6	0	7.386307	-1.916008	-1.256486
50	1	0	5.618087	-1.054845	-2.131994
51	6	0	7.364775	-2.134971	1.158013
52	1	0	5.566270	-1.498757	2.140410
53	6	0	8.002756	-2.290529	-0.067230
54	1	0	7.896987	-2.045262	-2.203419
55	1	0	7.853387	-2.443421	2.074697
56	17	0	9.611193	-2.973355	-0.113634
57	1	0	-2.548373	3.032104	-1.998650
58	6	0	-0.683476	2.058906	-2.380810
59	1	0	-0.083444	1.233181	-1.995047
60	1	0	-1.075439	1.745822	-3.358418
61	6	0	0.160253	3.287087	-2.547949
62	6	0	1.464598	3.343700	-2.295235
63	1	0	-0.353092	4.183151	-2.900364
64	1	0	2.035760	4.256523	-2.440425
65	1	0	2.004085	2.471394	-1.934106

TS2bS

Electronic Energies = -4307.072256
Sum of electronic and zero-point Energies = -4306.601986
Sum of electronic and thermal Enthalpies = -4306.561470
Sum of electronic and thermal Free Energies = -4306.680085

Standard orientation:

Center Number	Atomic Number	Atomic Type	Coordinates (Angstroms) X	Y	Z
1	8	0	-4.667337	-0.871113	0.536122
2	6	0	-4.782694	0.285982	-0.258016
3	6	0	-3.687844	1.270853	0.066620
4	6	0	-2.405066	1.286332	-0.411463
5	7	0	-3.795320	2.252728	1.008653
6	1	0	-1.866910	0.670343	-1.111625
7	7	0	-2.659158	2.858837	1.146974
8	7	0	-1.795677	2.271711	0.285365
9	6	0	-0.417254	2.583963	0.285710
10	6	0	0.176623	3.239596	-0.794931

11	6	0	0.399048	2.175578	1.344969
12	6	0	1.544221	3.477288	-0.835236
13	6	0	1.763997	2.423965	1.340505
14	6	0	2.317470	3.061739	0.239485
15	1	0	1.990275	3.976565	-1.686174
16	1	0	2.381535	2.107841	2.171259
17	17	0	4.039674	3.334429	0.202894
18	5	0	-3.845667	-1.994222	0.242597
19	6	0	-3.955727	-2.530011	-1.393886
20	1	0	-4.984203	-2.889580	-1.478924
21	1	0	-3.791583	-1.670905	-2.048413
22	6	0	-2.956478	-3.554582	-1.571279
23	6	0	-1.656392	-3.283324	-1.882101
24	1	0	-3.203089	-4.564822	-1.243626
25	1	0	-0.913278	-4.074020	-1.929773
26	1	0	-1.389330	-2.335962	-2.343223
27	6	0	-4.136209	-3.170328	1.329623
28	1	0	-4.107260	-2.708874	2.324842
29	6	0	-5.459522	-3.836744	1.114510
30	6	0	-6.582493	-3.584380	1.785781
31	1	0	-5.500215	-4.568632	0.304904
32	1	0	-7.516319	-4.085486	1.545205
33	1	0	-6.606302	-2.860046	2.597185
34	1	0	-3.351144	-3.940064	1.308968
35	8	0	-2.350343	-1.561281	0.272003
36	6	0	-1.369318	-2.359692	0.184113
37	1	0	-1.401651	-3.337086	0.665572
38	6	0	-0.043557	-1.788254	-0.059583
39	6	0	1.192662	-2.300468	0.254600
40	1	0	1.514364	-3.214244	0.729228
41	7	0	0.135755	-0.574256	-0.658041
42	7	0	1.392156	-0.306442	-0.721937
43	7	0	2.054563	-1.349148	-0.166347
44	6	0	3.470803	-1.335457	-0.122086
45	6	0	4.150798	-1.062096	1.067216
46	6	0	4.211224	-1.564994	-1.285088
47	6	0	5.537151	-1.008428	1.104245
48	6	0	5.597738	-1.510258	-1.273685
49	6	0	6.239138	-1.229614	-0.073924
50	1	0	6.054752	-0.793276	2.030991
51	1	0	6.161801	-1.685959	-2.181234
52	17	0	7.977823	-1.157397	-0.045551
53	1	0	-4.694825	0.038408	-1.324885
54	6	0	-6.184174	0.864266	-0.023046
55	1	0	-6.901136	0.062998	-0.226795
56	1	0	-6.266007	1.123917	1.038569
57	6	0	-6.480317	2.064836	-0.873899
58	6	0	-7.347022	2.076913	-1.883582
59	1	0	-5.925819	2.971797	-0.635856
60	1	0	-7.520168	2.971810	-2.475082
61	1	0	-7.921525	1.192017	-2.151150

62	17	0	3.259079	-0.785819	2.531450
63	17	0	3.394953	-1.939009	-2.770393
64	17	0	-0.803114	3.771770	-2.128555
65	17	0	-0.281312	1.294463	2.676387

TS2a’S

Electronic Energies = -2468.772851
Sum of electronic and zero-point Energies = -2468.263916
Sum of electronic and thermal Enthalpies = -2468.228202
Sum of electronic and thermal Free Energies = -2468.338620

Standard orientation:

Center Number	Atomic Number	Atomic Type	Coordinates (Angstroms) X	Y	Z
1	6	0	-4.047628	0.560098	0.283327
2	6	0	-3.187909	1.372708	-0.410583
3	7	0	-4.338700	0.288077	-1.900279
4	7	0	-4.742459	-0.098953	-0.674850
5	6	0	-5.763904	-1.072674	-0.523049
6	6	0	-6.774636	-1.155509	-1.477005
7	6	0	-5.742103	-1.930362	0.573088
8	6	0	-7.774833	-2.108027	-1.333266
9	6	0	-6.751608	-2.872653	0.727717
10	6	0	-7.756828	-2.952725	-0.228757
11	1	0	-8.565767	-2.182968	-2.070546
12	1	0	-6.744586	-3.544765	1.577929
13	6	0	-2.144256	2.327570	0.103548
14	8	0	-0.858158	1.801901	-0.099265
15	5	0	-0.143541	1.170173	0.979092
16	6	0	0.448351	2.427278	2.078925
17	1	0	-0.349691	3.167472	1.969550
18	1	0	0.492812	2.046099	3.099637
19	6	0	-0.915474	0.011346	1.814269
20	1	0	-1.372606	-0.672495	1.085171
21	1	0	-1.736260	0.466827	2.384840
22	6	0	-0.027891	-0.760020	2.736479
23	6	0	-0.031556	-0.692844	4.068336
24	1	0	0.707462	-1.405193	2.253956
25	1	0	0.669406	-1.262839	4.672314
26	1	0	-0.743238	-0.066419	4.603710
27	6	0	1.719153	2.867553	1.575895
28	6	0	1.914849	3.401922	0.331042
29	1	0	2.610221	2.521488	2.101572
30	1	0	2.908912	3.674519	-0.008208
31	1	0	1.078611	3.822885	-0.219864
32	6	0	3.917094	0.042206	-0.141314

33	6	0	3.115929	0.959030	-0.783221
34	6	0	1.738273	1.338288	-0.498208
35	8	0	1.116022	0.627114	0.377416
36	1	0	1.170021	1.831513	-1.283528
37	7	0	5.009331	0.994543	-1.827416
38	7	0	5.080465	0.093524	-0.821710
39	6	0	6.273247	-0.649133	-0.602274
40	6	0	7.068012	-0.995187	-1.691054
41	6	0	6.625485	-1.016231	0.692636
42	6	0	8.234597	-1.718322	-1.480309
43	6	0	7.784115	-1.753393	0.902968
44	6	0	8.577937	-2.094383	-0.186118
45	1	0	8.863154	-1.994391	-2.318963
46	1	0	8.069311	-2.045710	1.906825
47	1	0	-4.223420	0.418639	1.337570
48	1	0	3.740536	-0.629587	0.682940
49	7	0	-3.405233	1.174118	-1.741503
50	7	0	3.826207	1.513275	-1.805316
51	17	0	-9.026561	-4.143963	-0.040087
52	17	0	10.042154	-3.014999	0.077876
53	6	0	-2.218570	3.693587	-0.595015
54	1	0	-1.433330	4.325729	-0.166760
55	1	0	-1.981879	3.534250	-1.653516
56	6	0	-3.556505	4.359084	-0.456131
57	6	0	-3.784364	5.450041	0.270596
58	1	0	-4.384403	3.889875	-0.986844
59	1	0	-4.775563	5.888664	0.344545
60	1	0	-2.984162	5.949730	0.813321
61	1	0	-2.343872	2.482693	1.174490
62	1	0	-6.776545	-0.479297	-2.323496
63	1	0	-4.935751	-1.880436	1.296930
64	1	0	6.014017	-0.721044	1.538296
65	1	0	6.775174	-0.703429	-2.692568

TS2b'S

Electronic Energies = -4307.064083
Sum of electronic and zero-point Energies = -4306.594325
Sum of electronic and thermal Enthalpies = -4306.553585
Sum of electronic and thermal Free Energies = -4306.674624

Standard orientation:

Center Number	Atomic Number	Atomic Type	Coordinates (Angstroms) X	Y	Z
1	6	0	-4.218472	0.902267	0.378494
2	6	0	-3.144352	1.423863	-0.293107
3	7	0	-4.094666	0.034590	-1.669364

4	7	0	-4.781473	0.043991	-0.506356
5	6	0	-5.911813	-0.787793	-0.333217
6	6	0	-7.176393	-0.365308	-0.750395
7	6	0	-5.784413	-2.052236	0.248673
8	6	0	-8.293163	-1.175705	-0.598352
9	6	0	-6.885928	-2.881158	0.413540
10	6	0	-8.125748	-2.425805	-0.016346
11	1	0	-9.267714	-0.837319	-0.927708
12	1	0	-6.775764	-3.860119	0.863346
13	6	0	-2.109756	2.413508	0.174123
14	8	0	-0.809574	1.930789	0.001164
15	5	0	-0.233378	0.902167	0.830544
16	6	0	0.149298	1.592795	2.419544
17	1	0	-0.642318	2.343935	2.497860
18	1	0	0.043365	0.827783	3.189279
19	6	0	-1.044986	-0.490207	1.013700
20	1	0	-1.395228	-0.804342	0.020514
21	1	0	-1.940451	-0.309048	1.620910
22	6	0	-0.236918	-1.585974	1.629220
23	6	0	-0.421848	-2.105654	2.843734
24	1	0	0.590289	-1.962940	1.026450
25	1	0	0.223246	-2.886663	3.237550
26	1	0	-1.234114	-1.769889	3.486531
27	6	0	1.468338	2.151133	2.341507
28	6	0	1.834697	3.146681	1.475825
29	1	0	2.278530	1.584221	2.802194
30	1	0	2.860433	3.498132	1.432206
31	1	0	1.078815	3.792193	1.037484
32	6	0	3.971789	0.219127	-0.072469
33	6	0	3.253838	1.353381	-0.368295
34	6	0	1.837276	1.624786	-0.154122
35	8	0	1.121799	0.633320	0.248293
36	1	0	1.372202	2.415900	-0.737784
37	7	0	5.286020	1.771858	-0.989290
38	7	0	5.224467	0.518460	-0.475054
39	6	0	6.386761	-0.289071	-0.415588
40	6	0	6.789515	-1.035475	-1.525654
41	6	0	7.155972	-0.342635	0.749143
42	6	0	7.932203	-1.822433	-1.483217
43	6	0	8.301136	-1.123582	0.816842
44	6	0	8.669997	-1.852981	-0.306601
45	1	0	8.236375	-2.397698	-2.349009
46	1	0	8.891128	-1.157097	1.724186
47	1	0	-4.607913	1.050133	1.373326
48	1	0	3.703063	-0.730554	0.361273
49	7	0	-3.114527	0.869267	-1.538875
50	7	0	4.098634	2.269043	-0.924493
51	17	0	-4.225452	-2.602074	0.778404
52	17	0	-9.516442	-3.456706	0.182622
53	17	0	-7.363590	1.204798	-1.468750
54	17	0	5.848144	-0.987630	-2.982080

55	17	0	6.679940	0.582366	2.139687
56	17	0	10.103604	-2.838634	-0.237084
57	6	0	-2.211674	3.740729	-0.595247
58	1	0	-1.435828	4.409749	-0.208883
59	1	0	-1.977225	3.526053	-1.644959
60	6	0	-3.561760	4.386925	-0.486225
61	6	0	-3.804704	5.522468	0.163150
62	1	0	-4.386813	3.865550	-0.971165
63	1	0	-4.803980	5.945571	0.216165
64	1	0	-3.009726	6.075622	0.659530
65	1	0	-2.319292	2.620752	1.236800

TS2aR

Electronic Energy = -2468.759313
Sum of electronic and zero-point Energies = -2468.249501
Sum of electronic and thermal Enthalpies = -2468.214204
Sum of electronic and thermal Free Energies = -2468.320863

Standard orientation:

Center Number	Atomic Number	Atomic Type	Coordinates (Angstroms) X	Y	Z
1	8	0	-1.608372	3.236783	-0.195342
2	6	0	-1.876825	2.580345	-1.411897
3	6	0	-2.526617	1.234199	-1.219240
4	6	0	-3.856859	0.947603	-1.037636
5	7	0	-1.827842	0.071532	-1.082853
6	1	0	-4.742173	1.561376	-1.073921
7	7	0	-2.645133	-0.899550	-0.824055
8	7	0	-3.891425	-0.383636	-0.790629
9	6	0	-5.007571	-1.218345	-0.529025
10	6	0	-4.966117	-2.553710	-0.921673
11	6	0	-6.125550	-0.699887	0.117977
12	6	0	-6.053387	-3.377714	-0.664590
13	1	0	-4.086472	-2.939420	-1.422455
14	6	0	-7.221358	-1.518755	0.361075
15	1	0	-6.140160	0.332181	0.450432
16	6	0	-7.173834	-2.851119	-0.031190
17	1	0	-6.031619	-4.418742	-0.964959
18	1	0	-8.096722	-1.124883	0.864092
19	17	0	-8.549592	-3.887064	0.281318
20	5	0	-0.944550	2.657872	0.938996
21	6	0	-1.933807	1.835446	1.961425
22	1	0	-2.659269	2.561484	2.351531
23	1	0	-2.495376	1.117370	1.354945
24	6	0	-1.280782	1.103060	3.085343
25	6	0	-0.985982	-0.199308	3.077290

26	1	0	-0.994499	1.691043	3.960083
27	1	0	-0.485987	-0.679501	3.914426
28	1	0	-1.249101	-0.827539	2.228127
29	6	0	-0.233336	3.997515	1.832847
30	1	0	-0.253852	3.744014	2.896060
31	6	0	1.081555	4.141766	1.294168
32	6	0	2.170794	3.433047	1.740308
33	1	0	1.154261	4.605767	0.310675
34	1	0	3.115439	3.508310	1.210671
35	1	0	2.229899	3.122249	2.780409
36	1	0	-0.923475	4.805924	1.591766
37	8	0	0.201319	1.805436	0.457737
38	6	0	1.246676	1.535199	1.143663
39	1	0	1.135911	1.317403	2.205074
40	6	0	2.350418	0.864844	0.457033
41	6	0	3.315851	0.051593	1.002137
42	1	0	3.506035	-0.277154	2.010910
43	7	0	2.587835	0.974972	-0.880492
44	7	0	3.630634	0.278002	-1.184119
45	7	0	4.088569	-0.295251	-0.049078
46	6	0	5.235552	-1.133710	-0.076953
47	6	0	6.233201	-0.889332	-1.016154
48	6	0	5.345116	-2.182051	0.830901
49	6	0	7.356390	-1.704737	-1.045028
50	1	0	6.127149	-0.070430	-1.717382
51	6	0	6.476136	-2.988401	0.813390
52	1	0	4.548021	-2.385987	1.537349
53	6	0	7.470913	-2.742193	-0.125709
54	1	0	8.139943	-1.527017	-1.772196
55	1	0	6.573660	-3.808519	1.515011
56	17	0	8.892609	-3.759540	-0.153532
57	1	0	-2.617224	3.215546	-1.918529
58	6	0	-0.647125	2.486123	-2.331611
59	1	0	0.057586	1.744419	-1.951374
60	1	0	-1.000766	2.132864	-3.310647
61	6	0	0.032173	3.813060	-2.495415
62	6	0	1.335544	4.017429	-2.320023
63	1	0	-0.604802	4.649376	-2.788491
64	1	0	1.781880	4.996545	-2.475266
65	1	0	1.994129	3.204191	-2.022744

TS2bR

Electronic Energy = -4307.057457
Sum of electronic and zero-point Energies = -4306.587785
Sum of electronic and thermal Enthalpies = -4306.547081
Sum of electronic and thermal Free Energies = -4306.666720

Standard orientation:

Center Number	Atomic Number	Atomic Type	Coordinates (Angstroms) X	Y	Z
1	8	0	4.316302	-1.826968	-0.675116
2	6	0	4.670501	-0.466590	-0.672407
3	6	0	3.459904	0.426710	-0.732412
4	6	0	2.991884	1.319328	0.193467
5	7	0	2.571575	0.388679	-1.769620
6	1	0	3.372799	1.657995	1.143903
7	7	0	1.586423	1.185526	-1.532291
8	7	0	1.824719	1.770087	-0.332757
9	6	0	0.913447	2.711152	0.192843
10	6	0	0.158441	2.428665	1.335929
11	6	0	0.731721	3.951643	-0.429120
12	6	0	-0.762726	3.336520	1.840609
13	6	0	-0.193132	4.870884	0.048410
14	6	0	-0.930415	4.545082	1.178373
15	1	0	-1.339886	3.098976	2.725574
16	1	0	-0.326039	5.823309	-0.449295
17	17	0	-2.093269	5.688889	1.790927
18	5	0	3.457343	-2.366901	0.333575
19	6	0	3.824437	-2.017225	1.888905
20	1	0	3.698966	-0.932101	2.025582
21	1	0	3.114334	-2.502246	2.572812
22	6	0	5.215905	-2.412942	2.258357
23	6	0	5.552123	-3.378342	3.114810
24	1	0	6.015925	-1.886944	1.734914
25	1	0	6.589480	-3.642202	3.301491
26	1	0	4.796862	-3.939020	3.663447
27	6	0	3.450721	-4.114297	0.108633
28	1	0	3.814119	-4.492035	1.068575
29	6	0	2.117737	-4.527849	-0.208872
30	6	0	1.091366	-4.546176	0.701348
31	1	0	1.843165	-4.577864	-1.262516
32	1	0	0.091355	-4.835107	0.394316
33	1	0	1.313568	-4.627597	1.762925
34	1	0	4.171721	-4.292684	-0.688657
35	8	0	2.038182	-1.939976	0.028444
36	6	0	1.004006	-2.367460	0.649321
37	1	0	1.009093	-2.411041	1.739854
38	6	0	-0.288812	-2.097136	0.017695
39	6	0	-1.513155	-1.914732	0.612857
40	1	0	-1.848434	-1.936836	1.637661
41	7	0	-0.433902	-1.943726	-1.330758
42	7	0	-1.665122	-1.678332	-1.598846
43	7	0	-2.339773	-1.652672	-0.422120
44	6	0	-3.724831	-1.361216	-0.397093
45	6	0	-4.669014	-2.389680	-0.437305
46	6	0	-4.170188	-0.037690	-0.350282
47	6	0	-6.029614	-2.116088	-0.428587

48	6	0	-5.525549	0.261826	-0.341837
49	6	0	-6.434522	-0.788008	-0.381196
50	1	0	-6.753317	-2.921247	-0.461341
51	1	0	-5.861900	1.290631	-0.307989
52	17	0	-8.137996	-0.427418	-0.368866
53	1	0	5.217237	-0.204352	0.247542
54	6	0	5.607704	-0.240264	-1.866871
55	1	0	6.445596	-0.937510	-1.767837
56	1	0	5.051875	-0.509594	-2.772838
57	6	0	6.105100	1.172124	-1.964471
58	6	0	7.366727	1.545704	-1.766808
59	1	0	5.358693	1.929582	-2.203669
60	1	0	7.672103	2.585558	-1.844510
61	1	0	8.143495	0.821872	-1.527769
62	17	0	1.692560	4.383347	-1.808702
63	17	0	0.361236	0.914387	2.165601
64	17	0	-3.015595	1.256805	-0.297936
65	17	0	-4.138668	-4.041898	-0.499126

TS2a′R

Electronic Energy = -2468.772441
Sum of electronic and zero-point Energies = -2468.262906
Sum of electronic and thermal Enthalpies = -2468.227572
Sum of electronic and thermal Free Energies = -2468.333882

Standard orientation:

Center Number	Atomic Number	Atomic Type	Coordinates (Angstroms) X	Y	Z
1	8	0	-0.405018	-1.263875	-0.688183
2	6	0	0.056926	-0.068499	-0.110539
3	6	0	1.473561	0.234104	-0.521364
4	6	0	2.570082	0.578697	0.227023
5	7	0	1.864627	0.153845	-1.825192
6	1	0	2.721689	0.706748	1.286216
7	7	0	3.130386	0.417945	-1.914957
8	7	0	3.578313	0.680340	-0.671993
9	6	0	4.944802	0.987496	-0.444269
10	6	0	5.916447	0.418974	-1.263760
11	6	0	5.296997	1.844922	0.593943
12	6	0	7.255230	0.715847	-1.045100
13	1	0	5.624814	-0.250670	-2.063947
14	6	0	6.637213	2.129683	0.825168
15	1	0	4.534516	2.303302	1.214051
16	6	0	7.602649	1.564149	0.000586
17	1	0	8.019906	0.279834	-1.677487
18	1	0	6.921995	2.795311	1.631698

19	17	0	9.291920	1.928550	0.283700
20	5	0	0.030484	-2.604290	-0.409052
21	6	0	1.556928	-2.930490	0.060181
22	1	0	1.694469	-4.003917	-0.139291
23	1	0	2.274303	-2.413995	-0.589694
24	6	0	1.922779	-2.728271	1.500681
25	6	0	3.053951	-2.191654	1.959771
26	1	0	1.194629	-3.093116	2.226874
27	1	0	3.254985	-2.108355	3.024838
28	1	0	3.825065	-1.826375	1.284032
29	6	0	-0.352616	-3.537089	-1.792881
30	1	0	0.090589	-4.527741	-1.665782
31	6	0	-1.791719	-3.592353	-1.936016
32	6	0	-2.569656	-4.525095	-1.322131
33	1	0	-2.285912	-2.738388	-2.397803
34	1	0	-3.652808	-4.492725	-1.390383
35	1	0	-2.130332	-5.442463	-0.937828
36	1	0	0.145224	-2.991296	-2.598964
37	8	0	-0.887156	-3.311190	0.643992
38	6	0	-2.149722	-3.423262	0.678476
39	1	0	-2.531637	-4.303245	1.201222
40	6	0	-3.149222	-2.352496	0.604897
41	6	0	-3.190286	-1.095811	0.043460
42	1	0	-2.463658	-0.557415	-0.539082
43	7	0	-4.345224	-2.576194	1.227395
44	7	0	-5.100127	-1.538271	1.092044
45	7	0	-4.408714	-0.626084	0.370961
46	6	0	-4.972021	0.647151	0.079344
47	6	0	-5.894494	1.199911	0.962640
48	6	0	-4.584169	1.322396	-1.073526
49	6	0	-6.439749	2.446642	0.685602
50	1	0	-6.180999	0.661152	1.857779
51	6	0	-5.117777	2.576149	-1.343615
52	1	0	-3.880184	0.876582	-1.767289
53	6	0	-6.042177	3.124383	-0.461758
54	1	0	-7.159470	2.888756	1.364503
55	1	0	-4.822295	3.112451	-2.237748
56	17	0	-6.720331	4.700973	-0.802541
57	6	0	-0.156288	-0.002473	1.415552
58	1	0	-1.103800	-0.503092	1.634122
59	1	0	0.632728	-0.571696	1.919155
60	6	0	-0.211781	1.406991	1.932840
61	6	0	-1.309653	1.980515	2.419144
62	1	0	0.706385	1.991512	1.877995
63	1	0	-1.308975	3.009239	2.768013
64	1	0	-2.250643	1.438315	2.491255
65	1	0	-0.574863	0.717197	-0.551281

TS2b′R

Electronic Energy = -4307.061772
Sum of electronic and zero-point Energies = -4306.592224
Sum of electronic and thermal Enthalpies = -4306.551352
Sum of electronic and thermal Free Energies = -4306.674159

Standard orientation:

Center Number	Atomic Number	Atomic Type	Coordinates (Angstroms) X	Y	Z
1	8	0	1.392081	0.209822	1.366208
2	6	0	1.373566	1.023378	0.219842
3	6	0	2.792025	1.108914	-0.275082
4	6	0	3.876919	0.428274	0.215916
5	7	0	3.208202	1.866885	-1.328650
6	1	0	3.992241	-0.276201	1.022777
7	7	0	4.479704	1.697365	-1.515113
8	7	0	4.901655	0.821263	-0.579594
9	6	0	6.257844	0.424557	-0.530960
10	6	0	7.194424	1.172587	0.186851
11	6	0	6.691220	-0.717833	-1.208750
12	6	0	8.531377	0.801115	0.231891
13	6	0	8.021641	-1.113003	-1.178002
14	6	0	8.922063	-0.340700	-0.455554
15	1	0	9.248363	1.390502	0.789804
16	1	0	8.346136	-1.999802	-1.708210
17	17	0	10.596651	-0.820795	-0.410270
18	5	0	0.507542	-0.904659	1.439951
19	6	0	0.660227	-1.683789	2.859879
20	1	0	0.317017	-0.993836	3.643972
21	1	0	0.008040	-2.567401	2.893017
22	6	0	2.073007	-2.084569	3.129692
23	6	0	2.552055	-3.329466	3.112371
24	1	0	2.766010	-1.262088	3.308813
25	1	0	3.605794	-3.539231	3.275139
26	1	0	1.902615	-4.186913	2.943127
27	6	0	0.785955	-2.036931	0.119163
28	1	0	1.108595	-2.930978	0.659464
29	6	0	-0.424568	-2.213143	-0.616153
30	6	0	-1.522844	-2.872672	-0.117799
31	1	0	-0.581400	-1.586319	-1.494468
32	1	0	-2.430101	-2.949473	-0.708102
33	1	0	-1.394226	-3.610844	0.670253
34	1	0	1.604844	-1.624544	-0.471874
35	8	0	-0.905422	-0.411612	1.219812
36	6	0	-1.939621	-1.175177	1.186790
37	1	0	-2.087692	-1.901704	1.987287
38	6	0	-3.150242	-0.608027	0.588263
39	6	0	-4.464053	-0.919438	0.842142
40	1	0	-4.942035	-1.614250	1.514671

41	7	0	-3.113628	0.362151	-0.371597
42	7	0	-4.317305	0.666352	-0.717675
43	7	0	-5.155385	-0.106338	0.014453
44	6	0	-6.557764	0.005045	-0.150310
45	6	0	-7.229238	-0.794760	-1.077910
46	6	0	-7.289251	0.929981	0.598456
47	6	0	-8.600942	-0.685900	-1.258165
48	6	0	-8.661489	1.059393	0.434915
49	6	0	-9.295504	0.243709	-0.493964
50	1	0	-9.111847	-1.310272	-1.980626
51	1	0	-9.218728	1.782194	1.018125
52	17	0	-11.017260	0.389627	-0.706497
53	1	0	0.757970	0.572650	-0.573320
54	6	0	0.735671	2.385335	0.555736
55	1	0	-0.212598	2.156314	1.050525
56	1	0	1.378780	2.900849	1.278532
57	6	0	0.481755	3.246207	-0.650017
58	6	0	-0.710083	3.365380	-1.230588
59	1	0	1.339859	3.761465	-1.076216
60	1	0	-0.851070	3.978335	-2.116797
61	1	0	-1.584305	2.842298	-0.847269
62	17	0	5.549778	-1.666470	-2.110640
63	17	0	6.683263	2.592685	1.044871
64	17	0	-6.475229	1.935264	1.754957
65	17	0	-6.339606	-1.945469	-2.026022

8a

Electronic Energies = -2468.8395229
Sum of electronic and zero-point Energies = -2468.326347
Sum of electronic and thermal Enthalpies = -2468.290088
Sum of electronic and thermal Free Energies = -2468.400546

Standard orientation:

Center Number	Atomic Number	Atomic Type	Coordinates (Angstroms) X	Y	Z
1	8	0	0.120097	-0.532629	0.042991
2	6	0	0.322571	0.875588	0.026289
3	6	0	1.789660	1.197148	0.112128
4	6	0	2.837744	0.572490	-0.518886
5	7	0	2.298542	2.176615	0.905993
6	1	0	2.888170	-0.268612	-1.190793
7	7	0	3.591254	2.195328	0.795255
8	7	0	3.935776	1.221527	-0.065670
9	6	0	5.301432	0.982230	-0.375090
10	6	0	6.271138	1.225389	0.593346
11	6	0	5.650476	0.509085	-1.636010

12	6	0	7.606830	0.991298	0.295731
13	1	0	5.978534	1.594530	1.568901
14	6	0	6.985033	0.258965	-1.929699
15	1	0	4.892382	0.350832	-2.395098
16	6	0	7.950961	0.504132	-0.960794
17	1	0	8.371831	1.176809	1.040436
18	1	0	7.269542	-0.110268	-2.907883
19	17	0	9.632148	0.196066	-1.331179
20	5	0	0.353026	-1.370564	1.103645
21	6	0	1.137297	-1.042332	2.444779
22	1	0	1.865504	-1.855138	2.579844
23	1	0	1.701816	-0.106831	2.405184
24	6	0	0.202072	-1.034870	3.624249
25	6	0	0.050732	-0.020296	4.472385
26	1	0	-0.397181	-1.934560	3.759995
27	1	0	-0.653800	-0.071785	5.297304
28	1	0	0.631064	0.895379	4.377051
29	8	0	-0.142692	-2.639471	1.001901
30	6	0	-0.980665	-3.040092	-0.085235
31	6	0	-2.317180	-2.361370	0.004183
32	6	0	-2.744869	-1.216002	-0.617246
33	1	0	-2.271032	-0.563944	-1.331250
34	7	0	-3.326021	-2.808349	0.803306
35	7	0	-4.337456	-2.004232	0.712537
36	7	0	-4.000250	-1.024444	-0.151512
37	6	0	-4.903923	0.030309	-0.441969
38	6	0	-6.273699	-0.188769	-0.324659
39	6	0	-4.411274	1.271800	-0.833986
40	6	0	-7.157978	0.845695	-0.600304
41	1	0	-6.639760	-1.160379	-0.016080
42	6	0	-5.295221	2.304031	-1.122023
43	1	0	-3.343810	1.454178	-0.896663
44	6	0	-6.661637	2.081488	-1.001276
45	1	0	-8.226320	0.687052	-0.510508
46	1	0	-4.918582	3.274333	-1.424049
47	17	0	-7.776564	3.381707	-1.356204
48	6	0	-0.536848	1.580520	1.086551
49	1	0	-1.499440	1.058431	1.123225
50	1	0	-0.068706	1.463116	2.069482
51	6	0	-1.084589	-4.565682	-0.023271
52	1	0	-0.067659	-4.969188	-0.041660
53	1	0	-1.535089	-4.831108	0.939441
54	1	0	-0.039728	1.188110	-0.960706
55	6	0	-1.897860	-5.136915	-1.148834
56	6	0	-1.398912	-5.864331	-2.143975
57	1	0	-2.965020	-4.920433	-1.126517
58	1	0	-2.031150	-6.254029	-2.936503
59	1	0	-0.339349	-6.106119	-2.197606
60	6	0	-0.753833	3.038751	0.796966
61	6	0	-1.949976	3.617196	0.727844
62	1	0	0.144453	3.638035	0.660839

63	1	0	-2.053508	4.681215	0.535708
64	1	0	-2.868019	3.056476	0.890592
65	1	0	-0.512223	-2.746492	-1.031084

8b

Electronic Energies = -4307.138073
Sum of electronic and zero-point Energies = -4306.664871
Sum of electronic and thermal Enthalpies = -4306.623552
Sum of electronic and thermal Free Energies = -4306.744284

Standard orientation:

Center Number	Atomic Number	Atomic Type	Coordinates (Angstroms) X	Y	Z
1	6	0	-0.796424	2.322211	-1.299428
2	6	0	0.366258	2.784635	-0.736208
3	1	0	0.702892	3.757689	-0.418082
4	7	0	1.138406	1.675009	-0.599560
5	7	0	-0.661189	0.976638	-1.477910
6	7	0	0.490534	0.584506	-1.049795
7	6	0	2.429632	1.521580	-0.041617
8	6	0	3.481848	1.034660	-0.826165
9	6	0	2.672423	1.786305	1.310741
10	6	0	4.731384	0.781634	-0.276642
11	6	0	3.916363	1.544632	1.878674
12	6	0	4.926142	1.032297	1.074857
13	1	0	5.534236	0.401878	-0.895884
14	1	0	4.087128	1.746579	2.928798
15	17	0	3.257480	0.757679	-2.524103
16	17	0	6.483966	0.704667	1.779439
17	17	0	1.417283	2.430557	2.324493
18	6	0	-2.103072	2.994749	-1.650459
19	8	0	-3.173512	2.082920	-1.479654
20	5	0	-3.365840	1.260750	-0.397467
21	6	0	-2.831292	1.543559	1.077528
22	1	0	-2.563485	0.611857	1.585355
23	1	0	-1.925741	2.158541	1.051834
24	6	0	-3.901032	2.257335	1.861383
25	6	0	-4.536905	1.760707	2.921219
26	1	0	-4.176750	3.249871	1.502442
27	1	0	-5.315275	2.321797	3.430469
28	1	0	-4.295064	0.777767	3.320767
29	6	0	-3.199599	-1.693947	0.374233
30	6	0	-2.043922	-1.636154	-0.359790
31	1	0	-1.733569	-1.035252	-1.200758
32	6	0	0.170062	-2.675721	0.020094
33	6	0	1.104526	-2.017103	0.824332

34	6	0	0.633044	-3.432904	-1.056525
35	6	0	2.465319	-2.102971	0.569204
36	6	0	1.989658	-3.531462	-1.338090
37	6	0	2.884990	-2.860248	-0.516413
38	1	0	3.177429	-1.582654	1.198511
39	1	0	2.334695	-4.119460	-2.179379
40	7	0	-1.821388	-3.084966	1.320221
41	7	0	-3.018985	-2.592955	1.383979
42	7	0	-1.214570	-2.508530	0.261310
43	6	0	-4.444751	-0.864290	0.203865
44	17	0	4.591502	-2.970749	-0.855890
45	17	0	-0.500082	-4.269913	-2.072662
46	17	0	0.564105	-1.038871	2.149371
47	8	0	-4.169544	0.194268	-0.702617
48	6	0	-2.354330	4.323099	-0.914302
49	1	0	-3.422737	4.534913	-1.019552
50	1	0	-2.144739	4.199361	0.154780
51	6	0	-1.563266	5.473644	-1.474239
52	6	0	-2.098649	6.475930	-2.165580
53	1	0	-0.487112	5.472909	-1.307063
54	1	0	-1.488778	7.283701	-2.559710
55	1	0	-3.168112	6.526941	-2.358623
56	1	0	-2.096299	3.215863	-2.723924
57	6	0	-5.630751	-1.645207	-0.379405
58	1	0	-6.436330	-0.926850	-0.561938
59	1	0	-5.317874	-2.048672	-1.350537
60	6	0	-6.116326	-2.750545	0.513110
61	6	0	-7.330589	-2.793102	1.054506
62	1	0	-5.410830	-3.552695	0.722511
63	1	0	-7.638013	-3.615887	1.693762
64	1	0	-8.063069	-2.008388	0.875771
65	1	0	-4.735365	-0.466194	1.183921

9a

Electronic Energy = -2468.860997
Sum of electronic and zero-point Energies = -2468.347260
Sum of electronic and thermal Enthalpies = -2468.311590
Sum of electronic and thermal Free Energies = -2468.416800

Standard orientation:

Center Number	Atomic Number	Atomic Type	Coordinates (Angstroms) X	Y	Z
1	8	0	3.840080	1.422005	-1.160194
2	6	0	3.399300	0.507281	-2.160083
3	6	0	1.902279	0.387735	-2.069334
4	6	0	1.027670	1.303972	-1.539520

5	7	0	1.188449	-0.690535	-2.484491
6	1	0	1.174149	2.276901	-1.097149
7	7	0	-0.074355	-0.492072	-2.234719
8	7	0	-0.183882	0.716558	-1.654297
9	6	0	-1.434766	1.191700	-1.179323
10	6	0	-2.604303	0.821154	-1.834951
11	6	0	-1.469287	1.989386	-0.039358
12	6	0	-3.827358	1.257123	-1.344200
13	1	0	-2.554673	0.187775	-2.712270
14	6	0	-2.691466	2.440041	0.442556
15	1	0	-0.554767	2.237472	0.489551
16	6	0	-3.857885	2.066655	-0.213854
17	1	0	-4.748049	0.966671	-1.836414
18	1	0	-2.732887	3.053970	1.334504
19	17	0	-5.401295	2.599599	0.413689
20	5	0	3.667346	1.219164	0.180445
21	6	0	3.835569	2.492743	1.118009
22	1	0	4.378973	2.247535	2.038636
23	1	0	4.418551	3.244805	0.578047
24	6	0	2.474402	3.043067	1.459870
25	6	0	1.888577	4.072977	0.848039
26	1	0	1.924996	2.527739	2.248776
27	1	0	0.898095	4.413780	1.136265
28	1	0	2.389189	4.625136	0.055393
29	6	0	4.359701	-3.487876	1.052082
30	1	0	5.402107	-3.216157	1.201828
31	6	0	3.369327	-2.781611	1.589677
32	6	0	3.558024	-1.562101	2.442901
33	1	0	2.341906	-3.104916	1.427620
34	1	0	3.180019	-1.752432	3.455498
35	1	0	4.624243	-1.327505	2.530215
36	1	0	4.166916	-4.372455	0.452603
37	8	0	3.295054	-0.020777	0.616236
38	6	0	2.838864	-0.306996	1.926172
39	1	0	3.073195	0.511983	2.614940
40	6	0	1.344395	-0.442992	1.896044
41	6	0	0.534590	-1.066254	0.979546
42	1	0	0.756623	-1.560391	0.047977
43	7	0	0.556183	0.084625	2.870747
44	7	0	-0.685375	-0.188996	2.612559
45	7	0	-0.719083	-0.890656	1.464881
46	6	0	-1.962381	-1.334954	0.933530
47	6	0	-3.147305	-0.873494	1.501708
48	6	0	-1.987442	-2.206565	-0.151314
49	6	0	-4.365331	-1.279397	0.974875
50	1	0	-3.113520	-0.193841	2.343198
51	6	0	-3.206221	-2.605431	-0.684769
52	1	0	-1.074259	-2.567839	-0.606289
53	6	0	-4.384505	-2.135937	-0.119362
54	1	0	-5.291195	-0.917305	1.406104
55	1	0	-3.231314	-3.274302	-1.536874

56	17	0	-5.920311	-2.620514	-0.804808
57	1	0	3.655218	0.998262	-3.106904
58	6	0	4.111714	-0.851449	-2.126449
59	1	0	3.807332	-1.408778	-1.238313
60	1	0	3.768784	-1.410437	-3.005123
61	6	0	5.602501	-0.689290	-2.158823
62	6	0	6.407683	-1.008681	-1.150325
63	1	0	6.025852	-0.259008	-3.066942
64	1	0	7.482909	-0.866995	-1.209737
65	1	0	6.009488	-1.427064	-0.229494

9b

Electronic Energy = -4307.132949
Sum of electronic and zero-point Energies = -4306.659975
Sum of electronic and thermal Enthalpies = -4306.618303
Sum of electronic and thermal Free Energies = -4306.742787

Standard orientation:

Center Number	Atomic Number	Atomic Type	Coordinates (Angstroms) X	Y	Z
1	6	0	2.749300	1.617302	-0.763969
2	6	0	3.918791	1.524033	-0.053117
3	1	0	4.323856	2.066613	0.786465
4	7	0	4.576918	0.488136	-0.627975
5	7	0	2.761910	0.643409	-1.720122
6	7	0	3.856293	-0.039683	-1.637917
7	6	0	5.833980	-0.060480	-0.278570
8	6	0	7.007031	0.409122	-0.874175
9	6	0	5.922490	-1.090643	0.661102
10	6	0	8.244569	-0.127071	-0.545494
11	6	0	7.147784	-1.643073	1.007779
12	6	0	8.292157	-1.148726	0.394676
13	1	0	9.147407	0.243556	-1.014990
14	1	0	7.204821	-2.441435	1.737226
15	17	0	6.922860	1.687068	-2.046243
16	17	0	9.837002	-1.832196	0.819017
17	17	0	4.479335	-1.694309	1.413256
18	6	0	1.553494	2.519135	-0.599753
19	8	0	0.351219	1.767450	-0.639097
20	5	0	0.088537	0.614273	0.050002
21	6	0	0.870934	0.093741	1.339012
22	1	0	1.089864	-0.975587	1.248704
23	1	0	1.835955	0.597533	1.437332
24	6	0	0.047917	0.356177	2.572799
25	6	0	-0.449866	-0.573561	3.385817
26	1	0	-0.161005	1.404755	2.790148

27	1	0	-1.051456	-0.308419	4.250478
28	1	0	-0.271175	-1.633708	3.217554
29	6	0	-2.871065	-1.386045	0.052910
30	6	0	-3.798403	-0.415888	-0.221629
31	1	0	-3.721373	0.595571	-0.584911
32	6	0	-6.286184	-0.460140	-0.012535
33	6	0	-7.039001	-0.607069	-1.180277
34	6	0	-6.851041	0.222767	1.067406
35	6	0	-8.322619	-0.087766	-1.279547
36	6	0	-8.132236	0.752039	0.994188
37	6	0	-8.848265	0.585856	-0.184425
38	1	0	-8.896461	-0.209553	-2.189824
39	1	0	-8.558679	1.278229	1.839130
40	7	0	-4.801948	-2.267781	0.512702
41	7	0	-3.526488	-2.494657	0.495063
42	7	0	-4.983376	-1.004035	0.078598
43	6	0	-1.380593	-1.345530	-0.090117
44	1	0	-0.939844	-1.632336	0.869618
45	6	0	-0.881379	-2.323841	-1.167986
46	1	0	-1.177135	-3.334907	-0.869081
47	1	0	-1.398057	-2.085363	-2.105358
48	17	0	-10.458465	1.242833	-0.292754
49	17	0	-5.940347	0.417509	2.532713
50	17	0	-6.363118	-1.452960	-2.537116
51	6	0	0.606002	-2.236229	-1.365214
52	6	0	1.478750	-3.169130	-0.992588
53	1	0	0.980213	-1.327261	-1.835739
54	1	0	2.544456	-3.035107	-1.155919
55	1	0	1.160397	-4.097450	-0.521906
56	8	0	-1.017847	-0.024808	-0.443959
57	6	0	1.616696	3.429173	0.637231
58	1	0	0.610621	3.834478	0.781264
59	1	0	1.858567	2.831485	1.525295
60	6	0	2.596350	4.560499	0.487659
61	6	0	2.250700	5.844045	0.445825
62	1	0	3.649534	4.298032	0.395602
63	1	0	2.993162	6.628361	0.329987
64	1	0	1.212183	6.157342	0.529292
65	1	0	1.500018	3.167685	-1.481256

10a

Electronic Energy = -2845.545226
Sum of electronic and zero-point Energies = -2844.812724
Sum of electronic and thermal Enthalpies = -2844.762475
Sum of electronic and thermal Free Energies = -2844.904002

Standard orientation:

Center Number	Atomic Number	Atomic Type	Coordinates (Angstroms) X	Y	Z
8	-0.219713000	0.175780000	0.622324000		
6	-0.883481000	0.828217000	1.704764000		
6	-2.328096000	1.021804000	1.356089000		
6	-3.355449000	0.118275000	1.446877000		
7	-2.815468000	2.156270000	0.785685000		
1	-3.406438000	-0.890186000	1.823055000		
7	-4.073237000	2.000313000	0.516531000		
7	-4.417203000	0.760904000	0.912847000		
6	-5.735692000	0.274510000	0.717061000		
6	-6.805721000	1.163607000	0.745984000		
6	-5.936135000	-1.083961000	0.487651000		
6	-8.094215000	0.686546000	0.546066000		
1	-6.626780000	2.217661000	0.920957000		
6	-7.226600000	-1.564203000	0.303283000		
1	-5.091896000	-1.764354000	0.435847000		
6	-8.293258000	-0.673407000	0.332916000		
1	-8.937157000	1.367453000	0.564739000		
1	-7.396702000	-2.619340000	0.123890000		
17	-9.918364000	-1.274460000	0.092878000		
5	-0.102565000	-1.176259000	0.483100000		
6	-0.630474000	-2.274616000	1.510321000		
1	0.222811000	-2.924860000	1.755896000		
1	-0.963397000	-1.852556000	2.463069000		
6	-1.728277000	-3.126369000	0.926122000		
6	-2.860414000	-3.455302000	1.546360000		
1	-1.567130000	-3.505360000	-0.083447000		
1	-3.602346000	-4.089979000	1.070729000		
1	-3.072122000	-3.129983000	2.563374000		
8	0.545352000	-1.528718000	-0.673411000		
6	1.218441000	-2.757541000	-0.878213000		
6	2.618399000	-2.619244000	-0.359154000		
6	3.310034000	-1.476323000	-0.050078000		
1	3.025269000	-0.437449000	-0.023008000		
7	3.456856000	-3.680141000	-0.218587000		
7	4.624490000	-3.255745000	0.159210000		
7	4.551288000	-1.916400000	0.265247000		
6	5.689538000	-1.151107000	0.629755000		
6	6.611697000	-1.681478000	1.526757000		
6	5.867467000	0.115763000	0.081274000		
6	7.725431000	-0.932983000	1.883291000		
1	6.455964000	-2.671558000	1.938265000		
6	6.972899000	0.872288000	0.450273000		
1	5.161639000	0.506789000	-0.644172000		
6	7.891528000	0.339654000	1.347339000		
1	8.451942000	-1.333581000	2.580504000		
1	7.122913000	1.860197000	0.030826000		
17	9.287593000	1.287837000	1.809103000		
6	-0.136971000	2.136174000	1.971089000		

1	0.914992000	1.884410000	2.137924000
1	-0.196871000	2.749603000	1.067217000
6	1.207872000	-3.090286000	-2.380565000
1	1.746392000	-4.035600000	-2.502675000
1	0.166595000	-3.256894000	-2.677917000
1	-0.822900000	0.206930000	2.604363000
6	1.829712000	-2.016194000	-3.228554000
6	3.097745000	-2.020119000	-3.630152000
1	1.191936000	-1.174084000	-3.487270000
1	3.510240000	-1.204433000	-4.217074000
1	3.770406000	-2.838673000	-3.382619000
6	-0.689171000	2.893533000	3.144168000
6	-0.057800000	3.038174000	4.305474000
1	-1.673680000	3.336933000	3.006246000
1	-0.499304000	3.594819000	5.126856000
1	0.929411000	2.613153000	4.475122000
1	0.722984000	-3.580719000	-0.350518000
5	-0.019312000	1.821343000	-1.985551000
6	1.531769000	1.512346000	-1.821869000
1	1.658772000	0.461021000	-1.547054000
1	2.001096000	2.145977000	-1.061067000
6	-0.931125000	0.712667000	-2.660210000
1	-0.522067000	-0.285983000	-2.481114000
1	-0.794305000	0.916005000	-3.739902000
6	-0.630247000	3.254113000	-1.696328000
1	-1.196297000	3.559255000	-2.589140000
1	-1.398102000	3.113679000	-0.917616000
6	0.322783000	4.337799000	-1.296437000
6	0.658766000	5.389290000	-2.040494000
1	0.789280000	4.232687000	-0.315757000
1	1.371712000	6.131002000	-1.691579000
1	0.232623000	5.544928000	-3.029484000
6	-2.397702000	0.723021000	-2.356653000
6	-3.088153000	-0.335498000	-1.936133000
1	-2.924049000	1.668492000	-2.486621000
1	-4.153966000	-0.275472000	-1.736802000
1	-2.610661000	-1.302575000	-1.787434000
6	2.176671000	1.759103000	-3.160602000
6	2.914505000	2.819276000	-3.486785000
1	1.980705000	1.006941000	-3.925910000
1	3.326689000	2.936546000	-4.484983000
1	3.124430000	3.608236000	-2.769100000

10b

Electronic Energy = -4683.828364
Sum of electronic and zero-point Energies = -4683.135924
Sum of electronic and thermal Enthalpies = -4683.080224
Sum of electronic and thermal Free Energies = -4683.237169

Standard orientation:

Center Number	Atomic Number	Atomic Type	Coordinates (Angstroms) X	Y	Z
1	6	0	-2.904213	1.080160	-1.177692
2	6	0	-3.719472	0.019952	-1.481853
3	1	0	-3.766135	-0.664807	-2.314067
4	7	0	-4.572862	-0.055238	-0.432632
5	7	0	-3.301545	1.583216	0.027425
6	7	0	-4.302539	0.902791	0.478827
7	6	0	-5.614774	-0.986094	-0.208374
8	6	0	-6.901788	-0.746693	-0.695027
9	6	0	-5.374563	-2.157414	0.514327
10	6	0	-7.932323	-1.650019	-0.474968
11	6	0	-6.388206	-3.076133	0.749219
12	6	0	-7.654544	-2.804295	0.246541
13	1	0	-8.926886	-1.454121	-0.856044
14	1	0	-6.191596	-3.981030	1.310764
15	17	0	-7.224213	0.704917	-1.590776
16	17	0	-8.936496	-3.948710	0.529970
17	17	0	-3.782855	-2.476693	1.129873
18	6	0	-1.707819	1.645846	-1.889543
19	8	0	-0.555544	1.539615	-1.066565
20	5	0	-0.142450	0.469528	-0.312607
21	6	0	-0.273478	-1.035270	-0.805451
22	1	0	-0.145593	-1.769235	-0.007029
23	1	0	-1.293463	-1.167838	-1.191841
24	6	0	0.703875	-1.338255	-1.915756
25	6	0	1.444880	-2.440680	-1.997231
26	1	0	0.791924	-0.591956	-2.705115
27	1	0	2.124698	-2.603485	-2.828566
28	1	0	1.402430	-3.214847	-1.236189
29	6	0	2.251762	-0.674344	1.285843
30	6	0	3.440792	-0.196541	0.794608
31	1	0	3.844442	0.798825	0.687264
32	6	0	5.414485	-1.400792	-0.132910
33	6	0	5.689900	-0.947149	-1.426607
34	6	0	6.460016	-1.945086	0.620365
35	6	0	6.964805	-1.044104	-1.967552
36	6	0	7.739850	-2.063373	0.095617
37	6	0	7.970798	-1.610135	-1.196303
38	1	0	7.161702	-0.689261	-2.971571
39	1	0	8.538083	-2.492433	0.688510
40	7	0	3.385700	-2.418747	0.660785
41	7	0	2.268945	-2.032627	1.185621
42	7	0	4.111928	-1.306181	0.412051
43	6	0	1.106386	0.118797	1.843314
44	17	0	9.572974	-1.746620	-1.866393
45	17	0	6.181051	-2.459467	2.255079

46	17	0	4.431042	-0.242097	-2.392582
47	8	0	0.460617	0.899899	0.840328
48	6	0	-1.488171	1.065976	-3.292560
49	1	0	-0.501425	1.400562	-3.627018
50	1	0	-1.463606	-0.029188	-3.241933
51	6	0	-2.536305	1.514197	-4.273106
52	6	0	-2.296700	2.316651	-5.306123
53	1	0	-3.552817	1.160843	-4.103379
54	1	0	-3.088784	2.620557	-5.984346
55	1	0	-1.298681	2.697722	-5.511782
56	1	0	-1.864257	2.725318	-1.997599
57	6	0	0.094503	-0.691264	2.662055
58	1	0	-0.747516	-0.024316	2.871971
59	1	0	-0.280175	-1.531501	2.069686
60	6	0	0.689448	-1.195978	3.945857
61	6	0	0.368876	-0.725876	5.148391
62	1	0	1.437214	-1.982437	3.854534
63	1	0	0.832915	-1.112314	6.051467
64	1	0	-0.376807	0.056769	5.273726
65	1	0	1.537968	0.864514	2.516384
66	5	0	0.384541	3.519697	1.637026
67	6	0	-0.215433	3.140301	3.068523
68	1	0	-1.290673	2.937393	3.004838
69	1	0	0.276946	2.256693	3.492865
70	6	0	1.964877	3.531859	1.456714
71	1	0	2.420883	2.860789	2.200015
72	1	0	2.276174	4.546889	1.755318
73	6	0	-0.648831	4.163492	0.624400
74	1	0	-1.429231	3.404310	0.469791
75	1	0	-1.143357	4.969220	1.190560
76	6	0	2.533276	3.220036	0.101364
77	6	0	3.447712	3.940080	-0.546156
78	1	0	2.158443	2.311658	-0.367855
79	1	0	3.815284	3.640702	-1.524288
80	1	0	3.848876	4.862791	-0.131339
81	6	0	-0.162696	4.682748	-0.690919
82	6	0	-0.167960	5.960136	-1.069356
83	1	0	0.231411	3.932137	-1.374183
84	1	0	0.214036	6.269123	-2.038811
85	1	0	-0.549093	6.745870	-0.419129
86	6	0	0.035498	4.316438	3.973695
87	6	0	-0.872315	5.225740	4.327607
88	1	0	1.061644	4.439662	4.322357
89	1	0	-0.613507	6.071874	4.958015
90	1	0	-1.908354	5.149736	4.003679

TS3a

Electronic energy = -2845.527085

Sum of electronic and zero-point Energies = -2844.792129
Sum of electronic and thermal Enthalpies = -2844.744856
Sum of electronic and thermal Free Energies = -2844.87396

Standard orientation:

Center Number	Atomic Number	Atomic Type	Coordinates (Angstroms) X	Y	Z
1	8	0	-3.668867	-0.266020	-0.119952
2	6	0	-2.995108	-1.399687	-0.746056
3	6	0	-1.528196	-1.208612	-1.018405
4	6	0	-0.469572	-1.662116	-0.269445
5	7	0	-1.023809	-0.690450	-2.173176
6	1	0	-0.415195	-2.160230	0.683957
7	7	0	0.267813	-0.785101	-2.167025
8	7	0	0.622452	-1.380918	-1.011766
9	6	0	1.981417	-1.699893	-0.747498
10	6	0	2.832838	-1.975691	-1.814036
11	6	0	2.439543	-1.742468	0.565262
12	6	0	4.160840	-2.294201	-1.563476
13	1	0	2.453949	-1.943955	-2.828516
14	6	0	3.765798	-2.070401	0.816603
15	1	0	1.784177	-1.478662	1.387295
16	6	0	4.614188	-2.342873	-0.249640
17	1	0	4.834537	-2.511979	-2.384020
18	1	0	4.136843	-2.093535	1.834211
19	17	0	6.284489	-2.749994	0.065766
20	5	0	-3.431296	1.216676	-0.684453
21	6	0	-3.585543	1.293226	-2.304768
22	1	0	-2.762752	0.749294	-2.782141
23	1	0	-4.520544	0.782768	-2.564668
24	6	0	-3.628889	2.677485	-2.878799
25	6	0	-4.722292	3.328569	-3.275939
26	1	0	-2.672714	3.190813	-2.982285
27	1	0	-4.671230	4.336222	-3.678378
28	1	0	-5.708626	2.871000	-3.226095
29	8	0	-2.207723	1.586179	-0.054167
30	6	0	-1.194541	2.342838	-0.641042
31	6	0	0.124089	1.904224	-0.065562
32	6	0	1.371638	1.988212	-0.628936
33	1	0	1.711554	2.331202	-1.592197
34	7	0	0.262984	1.403837	1.192618
35	7	0	1.518067	1.176537	1.429589
36	7	0	2.211149	1.531855	0.331735
37	6	0	3.620824	1.387616	0.290962
38	6	0	4.344878	1.435139	1.479518
39	6	0	4.261937	1.182601	-0.926371
40	6	0	5.723600	1.278274	1.448425
41	1	0	3.827778	1.591986	2.418253
42	6	0	5.643811	1.041533	-0.960637

43	1	0	3.691179	1.105812	-1.844680
44	6	0	6.362563	1.091088	0.227266
45	1	0	6.297440	1.312959	2.367042
46	1	0	6.152374	0.878066	-1.903557
47	17	0	8.102570	0.918919	0.186673
48	6	0	-3.776456	-1.883208	-1.976157
49	1	0	-4.840982	-1.864744	-1.723165
50	1	0	-3.617578	-1.205578	-2.816821
51	6	0	-1.419895	3.853363	-0.406655
52	1	0	-0.633855	4.419203	-0.920050
53	1	0	-2.372750	4.118251	-0.882306
54	1	0	-3.054969	-2.198649	-0.008669
55	6	0	-1.458700	4.216371	1.047545
56	6	0	-0.703493	5.151081	1.615893
57	1	0	-2.155636	3.645730	1.659071
58	1	0	-0.772050	5.370292	2.677379
59	1	0	0.015012	5.731299	1.040550
60	6	0	-3.357020	-3.269816	-2.375772
61	6	0	-4.158489	-4.330955	-2.372433
62	1	0	-2.320170	-3.384188	-2.689653
63	1	0	-3.804110	-5.311057	-2.677675
64	1	0	-5.203064	-4.257496	-2.077328
65	1	0	-1.147959	2.165298	-1.724331
66	5	0	-3.936618	-0.518496	1.429741
67	6	0	-2.554374	-0.889774	2.205695
68	1	0	-1.768049	-0.211182	1.849821
69	1	0	-2.273970	-1.916981	1.935289
70	6	0	-4.408071	1.045910	2.158129
71	1	0	-3.495837	1.618732	2.313345
72	1	0	-4.900911	0.705686	3.066026
73	6	0	-5.159545	-1.583082	1.642430
74	1	0	-5.373037	-1.570931	2.719524
75	1	0	-6.070264	-1.235948	1.136906
76	6	0	-4.909753	-3.004681	1.233389
77	6	0	-4.499669	-3.987825	2.034757
78	1	0	-5.066743	-3.244648	0.181647
79	1	0	-4.322348	-4.990794	1.656750
80	1	0	-4.337942	-3.821609	3.097287
81	6	0	-5.215647	1.429565	1.090903
82	6	0	-4.744203	2.158888	-0.030406
83	1	0	-6.197388	0.961078	1.021239
84	1	0	-5.504629	2.361603	-0.781476
85	1	0	-4.194409	3.063403	0.234089
86	6	0	-2.626376	-0.815648	3.698701
87	6	0	-2.573430	-1.849065	4.538509
88	1	0	-2.728269	0.183105	4.125232
89	1	0	-2.635238	-1.713441	5.614680
90	1	0	-2.452147	-2.868335	4.177911

TS3b

Electronic energy = -4683.810783
Sum of electronic and zero-point Energies = -4683.115548
Sum of electronic and thermal Enthalpies = -4683.063128
Sum of electronic and thermal Free Energies = -4683.204954

Standard orientation:

Center Number	Atomic Number	Atomic Type	Coordinates (Angstroms) X	Y	Z
1	8	0	3.977626	-0.214923	0.001152
2	6	0	3.590067	1.145586	-0.334232
3	6	0	2.127018	1.361584	-0.593603
4	6	0	1.319642	2.203087	0.127311
5	7	0	1.396408	0.875394	-1.637432
6	1	0	1.493479	2.800237	1.008519
7	7	0	0.194014	1.346057	-1.587738
8	7	0	0.132899	2.169202	-0.515908
9	6	0	-1.049084	2.881073	-0.206808
10	6	0	-1.484780	3.925824	-1.027175
11	6	0	-1.804834	2.554739	0.923550
12	6	0	-2.658968	4.614032	-0.752842
13	6	0	-2.974275	3.239772	1.225555
14	6	0	-3.386813	4.255520	0.374287
15	1	0	-2.989112	5.416830	-1.400069
16	1	0	-3.554353	2.972960	2.100053
17	5	0	3.587493	-1.445913	-0.928670
18	6	0	3.823343	-1.135287	-2.509519
19	1	0	3.091831	-0.384579	-2.830688
20	1	0	4.823996	-0.700412	-2.610330
21	6	0	3.728663	-2.310630	-3.435433
22	6	0	4.757577	-2.979127	-3.957896
23	1	0	2.724919	-2.635852	-3.708604
24	1	0	4.608002	-3.825606	-4.622399
25	1	0	5.787805	-2.696120	-3.749169
26	8	0	2.306989	-1.821679	-0.440759
27	6	0	1.289343	-2.386720	-1.211042
28	6	0	-0.032886	-2.111614	-0.550404
29	6	0	-1.271091	-2.065581	-1.133192
30	1	0	-1.601747	-2.116902	-2.158122
31	7	0	-0.196313	-2.006780	0.799350
32	7	0	-1.453268	-1.893001	1.079391
33	7	0	-2.128765	-1.931121	-0.090225
34	6	0	-3.540880	-1.887744	-0.099841
35	6	0	-4.284983	-2.934973	0.455807
36	6	0	-4.232299	-0.808033	-0.659042
37	6	0	-5.672405	-2.893777	0.494254
38	6	0	-5.619387	-0.749507	-0.641566
39	6	0	-6.318826	-1.793739	-0.052557
40	1	0	-6.232565	-3.709878	0.933499

41	1	0	-6.137667	0.096582	-1.075585
42	6	0	4.503173	1.747195	-1.415148
43	1	0	5.527807	1.424831	-1.204423
44	1	0	4.226671	1.377981	-2.404592
45	6	0	1.466065	-3.914835	-1.390503
46	1	0	0.663095	-4.278067	-2.042138
47	1	0	2.411074	-4.087207	-1.918871
48	1	0	3.792036	1.714936	0.571031
49	6	0	1.453606	-4.663877	-0.091606
50	6	0	0.527969	-5.552939	0.257251
51	1	0	2.252860	-4.426566	0.609523
52	1	0	0.554878	-6.055603	1.219800
53	1	0	-0.296210	-5.805992	-0.406889
54	6	0	4.419808	3.248329	-1.410023
55	6	0	5.437191	4.058565	-1.130854
56	1	0	3.448460	3.676740	-1.655873
57	1	0	5.323146	5.138660	-1.146143
58	1	0	6.425108	3.672037	-0.888963
59	1	0	1.255598	-1.926606	-2.207364
60	5	0	4.093213	-0.424464	1.593935
61	6	0	2.682220	-0.047977	2.312065
62	1	0	1.869871	-0.475309	1.711551
63	1	0	2.561233	1.043352	2.317395
64	6	0	4.318352	-2.145652	1.857635
65	1	0	3.346007	-2.631571	1.794001
66	1	0	4.766210	-2.158251	2.850215
67	6	0	5.398943	0.362359	2.199288
68	1	0	5.404728	0.090671	3.263671
69	1	0	6.328936	-0.033325	1.771118
70	6	0	5.442040	1.857746	2.099167
71	6	0	5.004552	2.719331	3.019118
72	1	0	5.857851	2.270339	1.178568
73	1	0	5.054496	3.793156	2.859762
74	1	0	4.591141	2.376198	3.965117
75	6	0	5.187826	-2.358560	0.776255
76	6	0	4.764785	-2.711736	-0.519213
77	1	0	6.211924	-2.004471	0.895080
78	1	0	5.559401	-2.783647	-1.258128
79	1	0	4.083103	-3.559445	-0.563363
80	6	0	2.560048	-0.537279	3.720021
81	6	0	2.555541	0.221208	4.816859
82	1	0	2.471920	-1.617498	3.844849
83	1	0	2.470919	-0.211056	5.810357
84	1	0	2.620346	1.306090	4.757225
85	17	0	-4.862179	5.110575	0.733577
86	17	0	-1.303978	1.255377	1.951553
87	17	0	-3.480995	-4.341494	1.081045
88	17	0	-3.357084	0.490783	-1.408176
89	17	0	-0.538776	4.403450	-2.403801
90	17	0	-8.059932	-1.728066	-0.011061
